# Supplementary material for: The environmentally-regulated interplay between local three-dimensional chromatin organisation and transcription of proVWX in E. coli
Source: Nat Commun. 2023 Nov 17;14:7478. doi: 10.1038/s41467-023-43322-y (PMC10656529; doi:10.1038/s41467-023-43322-y)
Supplement: Supplementary file 1 — Supplementary Information [file 41467_2023_43322_MOESM1_ESM.pdf]

Supplementary figures and tables:

The environmentally-regulated interplay between local three-dimensional chromatin organisation and transcription of *proVWX* in *E. coli*

Fatema-Zahra M. Rashid[1,2,3], Frédéric G.E. Crémazy[1,4], Andreas Hofmann[5], David Forrest[6], David C. Grainger[6], Dieter W. Heermann[5], Remus T. Dame[1,2,3*]

[1] Macromolecular Biochemistry, Leiden Institute of Chemistry, Leiden University, Leiden 2333CC, The Netherlands.
[2] Centre for Microbial Cell Biology, Leiden University, Leiden 2333CC, The Netherlands.
[3] Centre for Interdisciplinary Genome Research, Leiden University, Leiden 2333CC, The Netherlands.
[4] Laboratoire Infection et Inflammation, INSERM, UVSQ, Université Paris-Saclay, Versailles, 78180, France.
[5] Statistical Physics and Theoretical Biophysics, Heidelberg University, Heidelberg D-69120, Germany.
[6] School of Biosciences, University of Birmingham, Edgbaston B15 2TT, UK.

[*]To whom correspondence should be addressed. Tel: +31 71 527 5605; E-mail: rtdame@chem.leidenuniv.nl

Supplementary figures:

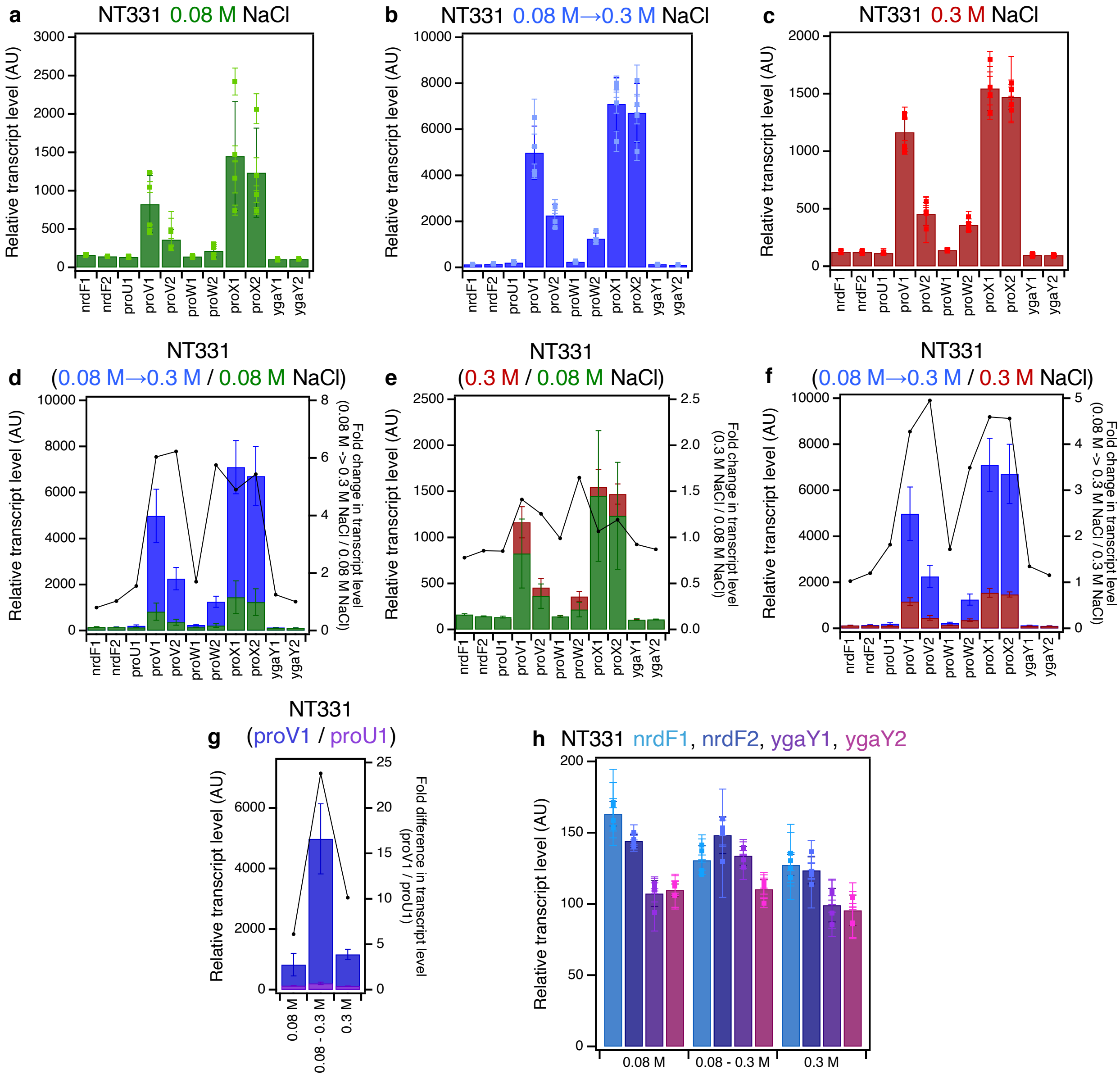

Supplementary Fig. 1: The RT-qPCR profile of *proVWX* and its flanking regions in NT331 during (a) exponential growth in M9 medium with 0.08 M NaCl, (b) hyperosmotic shock in M9 medium from 0.08 M to 0.3 M NaCl, and (c) exponential growth in M9 medium with 0.3 M NaCl. The fold changes in transcript levels of *proVWX* and its flanking regions between (d) a hyperosmotic shock and exponential growth at 0.08 M NaCl, (e) exponential growth at 0.3 M NaCl and 0.08 M NaCl, and (f) a hyperosmotic shock and exponential growth at 0.3 M NaCl. (g) The difference in the transcript level of the *proV*1 amplicon compared to the *proU*1 amplicon during exponential growth at 0.08 M NaCl, following a hyperosmotic shock, and during exponential growth at 0.3 M NaCl. (h) The transcript levels of amplicons flanking *proVWX* during exponential growth at 0.08 M NaCl, following a hyperosmotic shock, and during exponential growth at 0.3 M NaCl. Y-axes: All bar graphs and data points with error bars show relative transcript levels in arbitrary units and are plotted on the left y-axis. Plots without error bars show fold changes in transcript levels and correspond to the right y-axis. Internal control: *hcaT*. See also Fig. 1. Data (Supplementary Fig. 1a-h) are presented as mean values +/- standard devation. Dot plots (Supplementary Fig. 1a-c, and 1h): n=3 technical replicates of a biologically independent culture. Bar graphs (Supplementary Fig. 1a-h): n=4 biologically independent cultures. Source data are provided as a Source Data file.

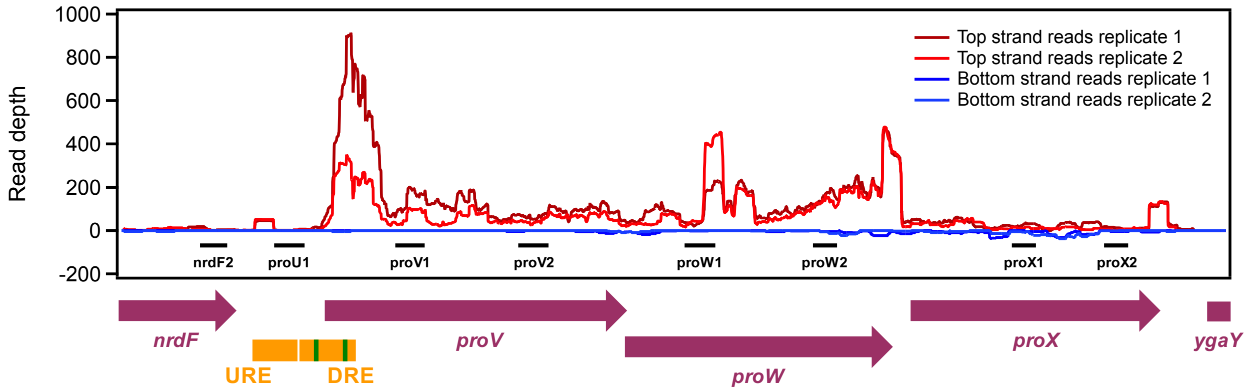

Supplementary Fig. 2: The Term-seq profile of the *proVWX* operon shows the presence of transcription termination sites downstream of *proW*1, and between *proW*2 and *proX*1. Source data are provided as a Source Data file.

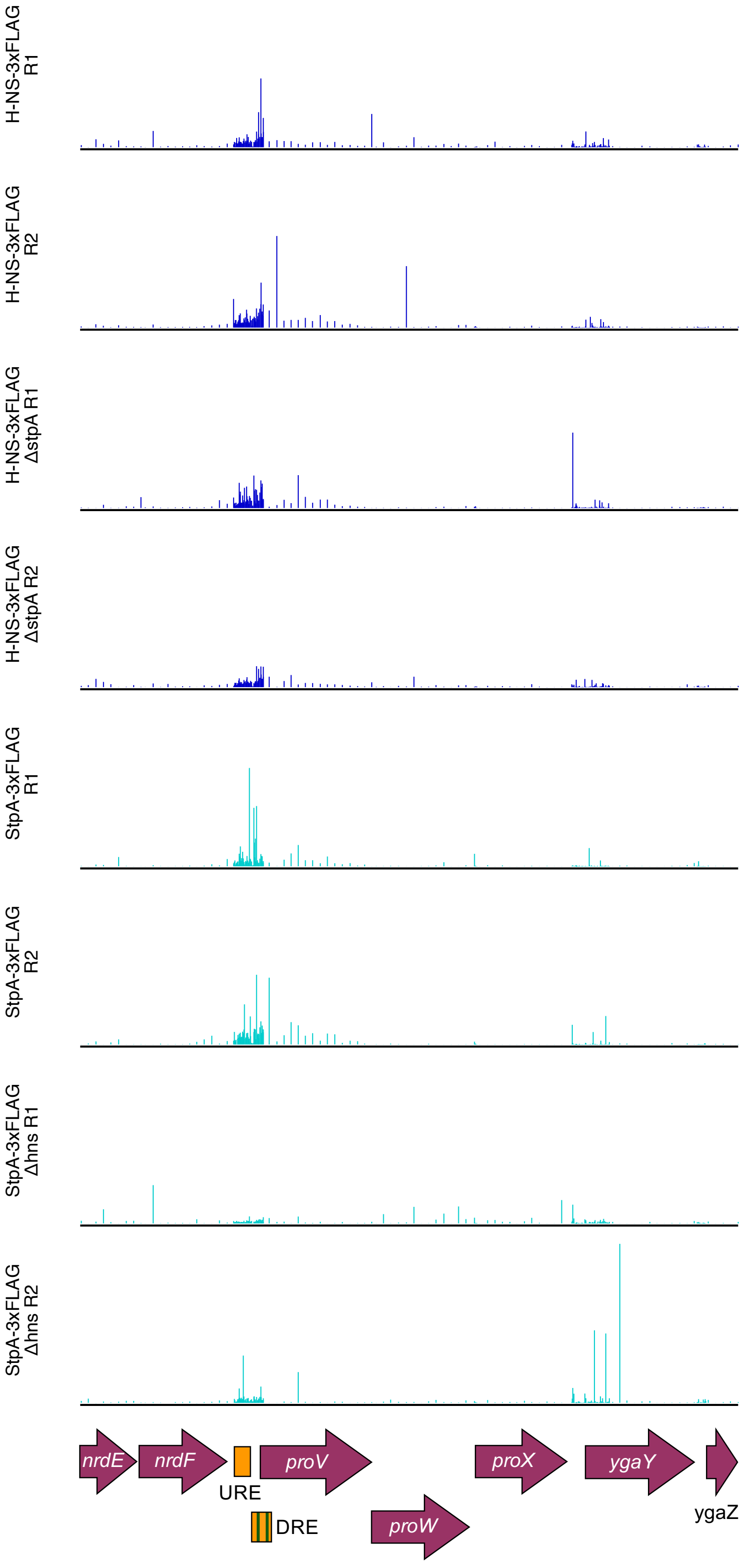

Supplementary Fig. 3: The chromatin immunoprecipitation (ChIP) profiles of H-NS-3xFLAG and StpA-3XFLAG at *proVWX* [1]. H-NS-3xFLAG: H-NS occupies the regulatory region of *proVWX* and the *proV* ORF. H-NS also binds the cryptic *ygaY* gene positioned downstream of *proVWX*. H-NS-3xFLAG Δ*stpA*: The occupancy and distribution of H-NS on *proVWX* is not affected by the absence of StpA within the H-NS—DNA nucleoprotein structure. StpA-3xFLAG: The occupancy of StpA on *proVWX* overlaps with the distribution of H-NS. The H-NS—DNA nucleoprotein at *proVWX* is interspersed with StpA. StpA-3xFLAG Δ*hns*: The distribution of StpA on *proVWX* is affected by the absence of H-NS. In a Δ*hns* background, StpA occupies the regulatory elements upstream of the *proV* ORF, but the nucleoprotein structure on the *proV* ORF is lost. R1 and R2 are biological replicates. The purple arrows represent ORFs. The orange bars mark the *proVWX* upstream and downstream regulatory elements (URE and DRE). The green bars within the DRE designate high-affinity H-NS binding sites. Source data are provided as a Source Data file.

```
                   H-NS protected     DNaseI sensitive          P2
                      regions              regions

NT331:  -40     AGGGGTTGCCTCAGATTCTCAGTATGTTAGGGTAGAAAAAAGTGACTATTTCCATTGGGTAATATATCGA     +30
NT644:  -40     AGGGGTTGCCTCAGATTCTCAGTATGTTAGGGTAGAAGCAAGTGACGCTTTCCATTGGGTAGTACATCGA     +30
                    -35                    -10   IHF binding site        High-affinity H-NS binding site

        +31     CATAGACAAATAAAGGAATCTTTCTATTGCATGGCAATTAAATTAGAAATTAAAAATCTTTATAAAATAT     +100
        +31     CATAGACAAATAAAGGAATCTTTCTATTGCATGGCAATCAAACTCGAGATCAAGAACCTCTACAAGATCT     +100
                     Shine-Dalgarno sequence

        +101    TTGGCGAGCATCCACAGCGAGCGTTCAAATATATCGAACAAGGACTTTCAAAAGAACAAATTCTGGAAAA     +170
        +101    TCGGCGAGCATCCACAGCGAGCGTTCAAGTACATCGAGCAGGGGCTCTCGAAGGAGCAGATCCTGGAGAA     +170
                               High-affinity H-NS binding site

        +171    AACTGGG     +178
        +171    GACGGGG     +178
```

Supplementary Fig. 4: A comparison between the wild-type *proVWX* DRE in NT331 and the mutant DRE in NT644. H-NS protected and DNaseI sensitive regions of the DRE as determined in REF[2] are represented in black and grey, respectively. Point mutations to the DRE are marked in red. The P2 -35 sequence, -10 sequence, and TSS are in blue. The Shine-Dalgarno sequence is coloured yellow. The pair of high-affinity H-NS binding sites of the DRE[3] are highlighted in green, and the IHF binding site encompassing P2 is in purple.

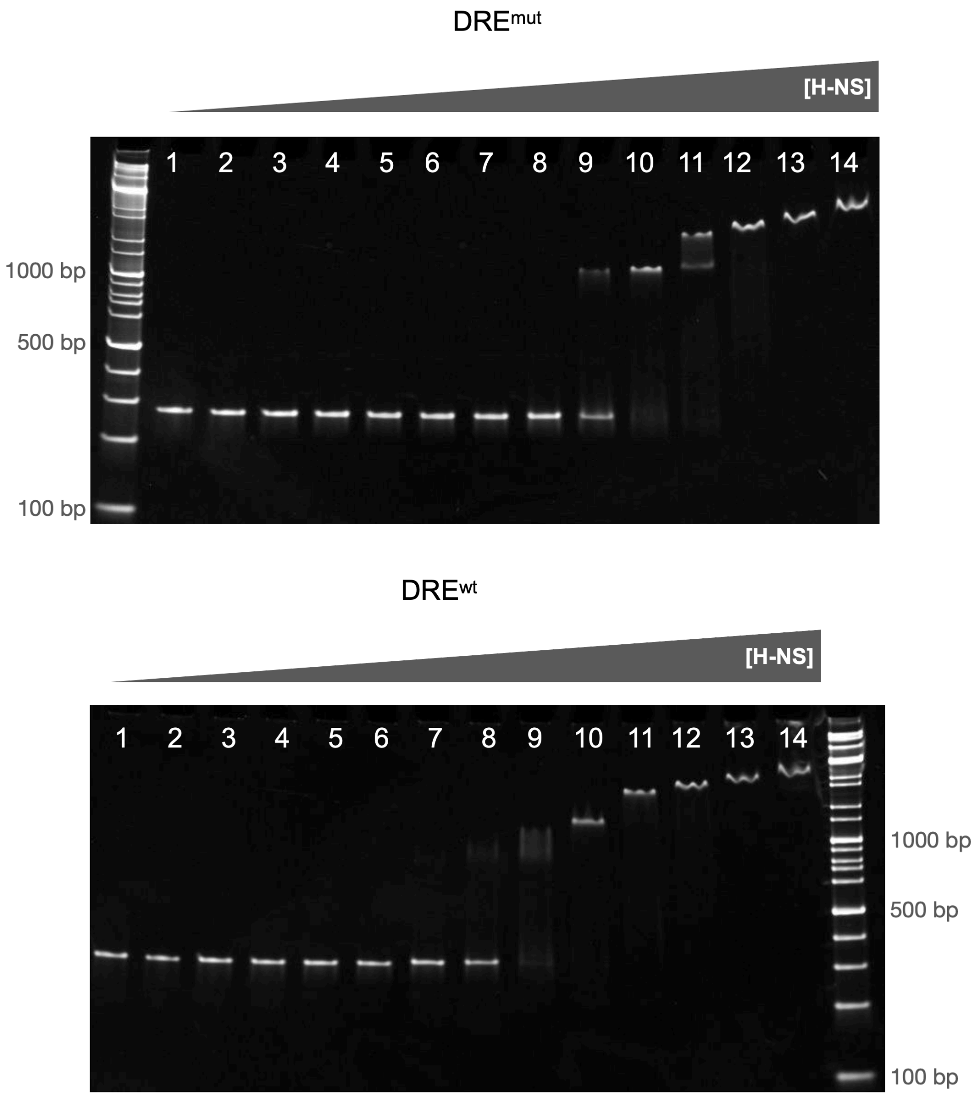

Lane	[H-NS] (nM)
1	31.7
2	52.8
3	88.0
4	146.6
5	244.4
6	407.3
7	678.8
8	1131
9	1885
10	3142
11	5238
12	8730
13	14550
14	24250

Supplementary Fig. 5: The mutated DRE (top) has a lower affinity for H-NS than the wild-type DRE (bottom). The EMSA gels shown here are representative of an experiment performed four times for DRE^{mut} and three times for DRE^{wt}. Images of all the EMSA gels are provided in the Source Data at the end of this document.

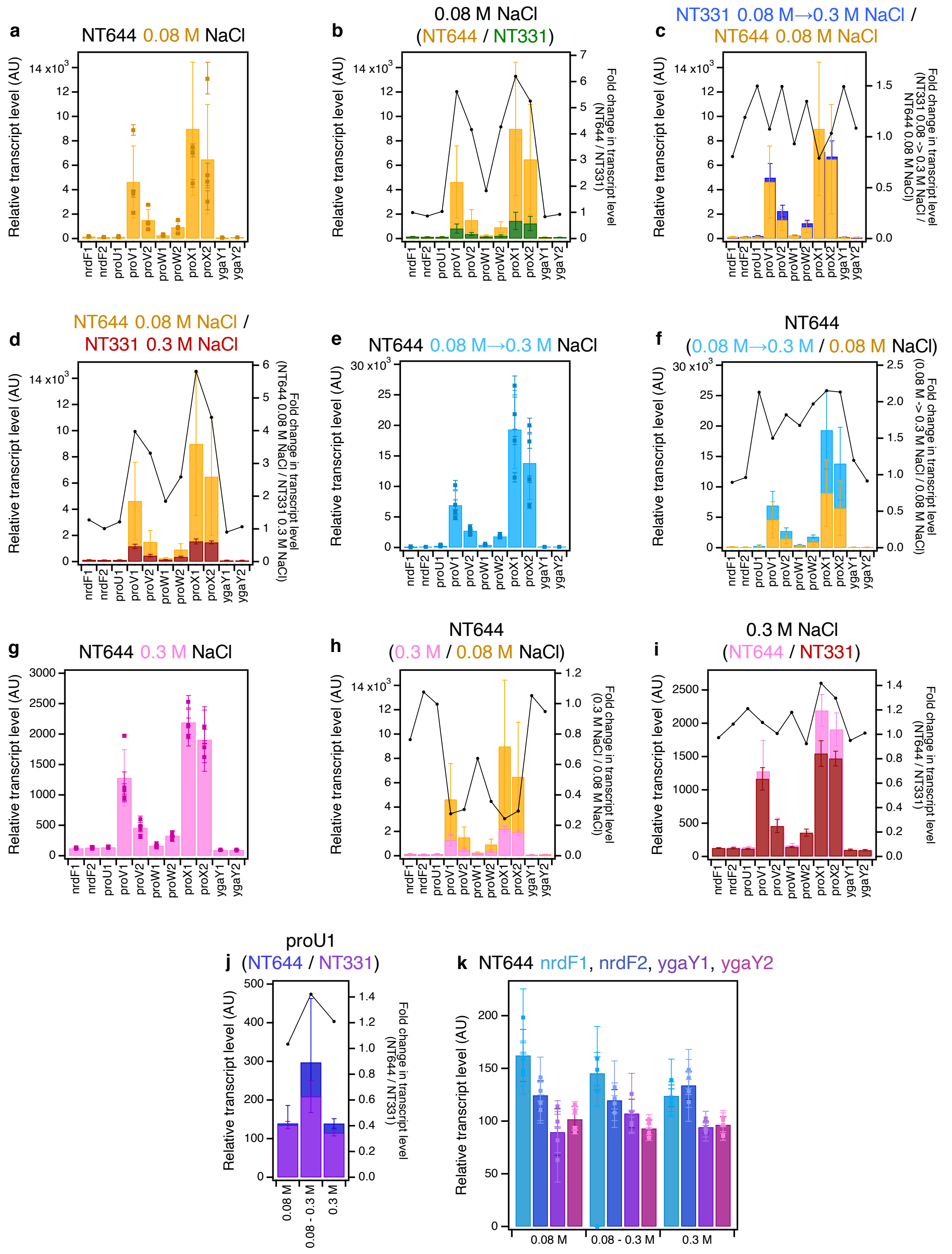

Supplementary Fig. 6: The RT-qPCR profile of *proVWX* and its flanking regions in NT644 (a) during exponential growth in M9 medium with 0.08 M NaCl. A comparison of transcript levels of *proVWX* and its flanking regions (b) between NT644 and NT331 during exponential growth in M9 medium with 0.08 M NaCl, (c) between NT644 growing exponentially at 0.08 M NaCl and NT331 subjected to a hyperosmotic shock from 0.08 M to 0.3 M NaCl, and (d) between NT644 at 0.08 M NaCl and NT331 at 0.3 M NaCl. The RT-qPCR profile of *proVWX* and its flanking regions in NT644 (e) after a hyperosmotic shock from 0.08 M to 0.3 M NaCl, and (f) the fold change in transcript levels compared to exponential growth at 0.08 M NaCl. The RT-qPCR profile of *proVWX* and its flanking regions (g) during exponential growth in M9 medium with 0.3 M NaCl, and a comparison of this profile with that of (h) exponential growth of NT644 in M9 medium with 0.08 M NaCl, and (i) exponential growth of NT331 in M9 medium with 0.3 M NaCl. (j) The fold change in transcript level of the *proU*1 amplicon between NT644 and NT331 during exponential growth at 0.08 M NaCl, following a hyperosmotic shock, and during exponential growth at 0.3 M NaCl. (k) The relative transcript level in NT644 at amplicons flanking *proVWX* during exponential growth at 0.08 M NaCl, following a hyperosmotic shock, and during exponential growth at 0.3 M NaCl. Y-axes: All bar graphs and data points with error bars show relative expression levels in arbitrary units and are plotted on the left y-axis. Plots without error bars show fold-change in expression level and correspond to the right y-axis. Internal control: *hcaT*. See also Fig. 2. Data (Supplementary Fig. 6a-k) are presented as mean values +/- standard devation. Dot plots (Supplementary Fig. 6a, 6e, 6g, and 6k): n=3 technical replicates of a biologically independent culture. Bar graphs (Supplementary Fig. 6a-k): n=4 biologically independent cultures. Source data are provided as a Source Data file.

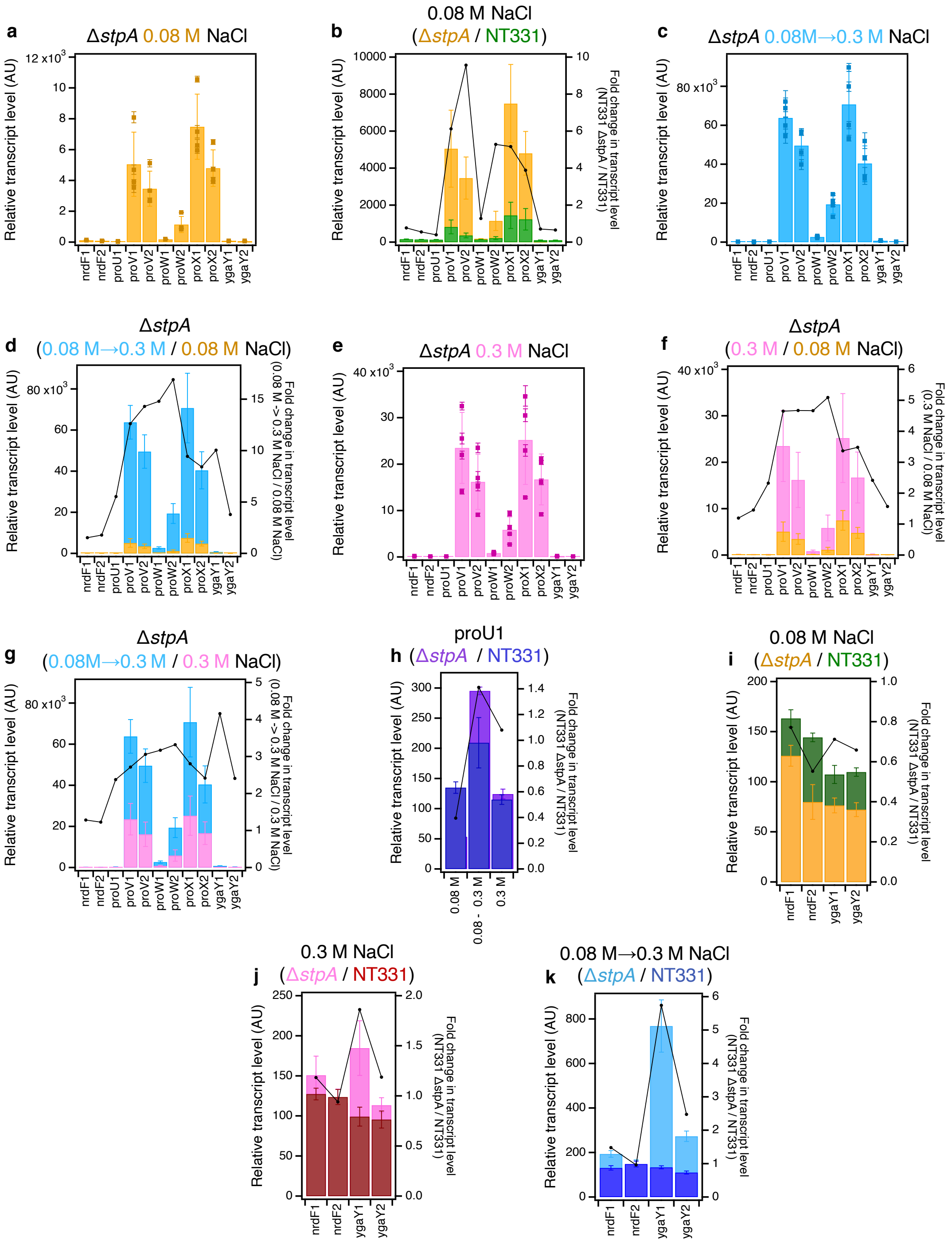

Supplementary Fig. 7: The RT-qPCR profile of the *proVWX* operon and its flanking regions in NT331 Δ*stpA* (a) during exponential growth at 0.08 M NaCl and (b) the fold-change in the transcript levels of the amplicons compared to NT331. (c) The RT-qPCR profile of the *proVWX* operon and its flanking regions in NT331 Δ*stpA* upon a hyperosmotic shock from 0.08 M to 0.3 M NaCl, and (d) the fold-change in transcript levels of the amplicons in comparison to exponential growth at 0.08 M NaCl. (e) The RT-qPCR profile of the *proVWX* operon and its flanking regions in NT331 Δ*stpA* during exponential growth at 0.3 M NaCl, and the fold-change in transcript levels of the amplicons with respect to (f) exponential growth at 0.08 M NaCl, and (g) a hyperosmotic shock. (h) The fold difference in transcript level of amplicon *proU*1 between NT331 Δ*stpA* and NT331. The fold change in transcript levels of the *nrdF* and *ygaY* amplicons between NT331 Δ*stpA* and NT331 (i) during exponential growth at 0.08 M NaCl, (j) exponential growth at 0.3 M NaCl, and (k) following a hyperosmotic shock. Y-axes: All bar graphs and data points with error bars show relative transcript levels in arbitrary units and are plotted on the left y-axis. Plots without error bars show fold-change in transcript level and correspond to the right y-axis. Internal control: *hcaT*. See also Fig. 3. Data (Supplementary Fig. 7a-k) are presented as mean values +/- standard devation. Dot plots (Supplementary Fig. 7a, 7c, and 7e): n=3 technical replicates of a biologically independent culture. Bar graphs (Supplementary Fig. 7a-k): n=4 biologically independent cultures. Source data are provided as a Source Data file.

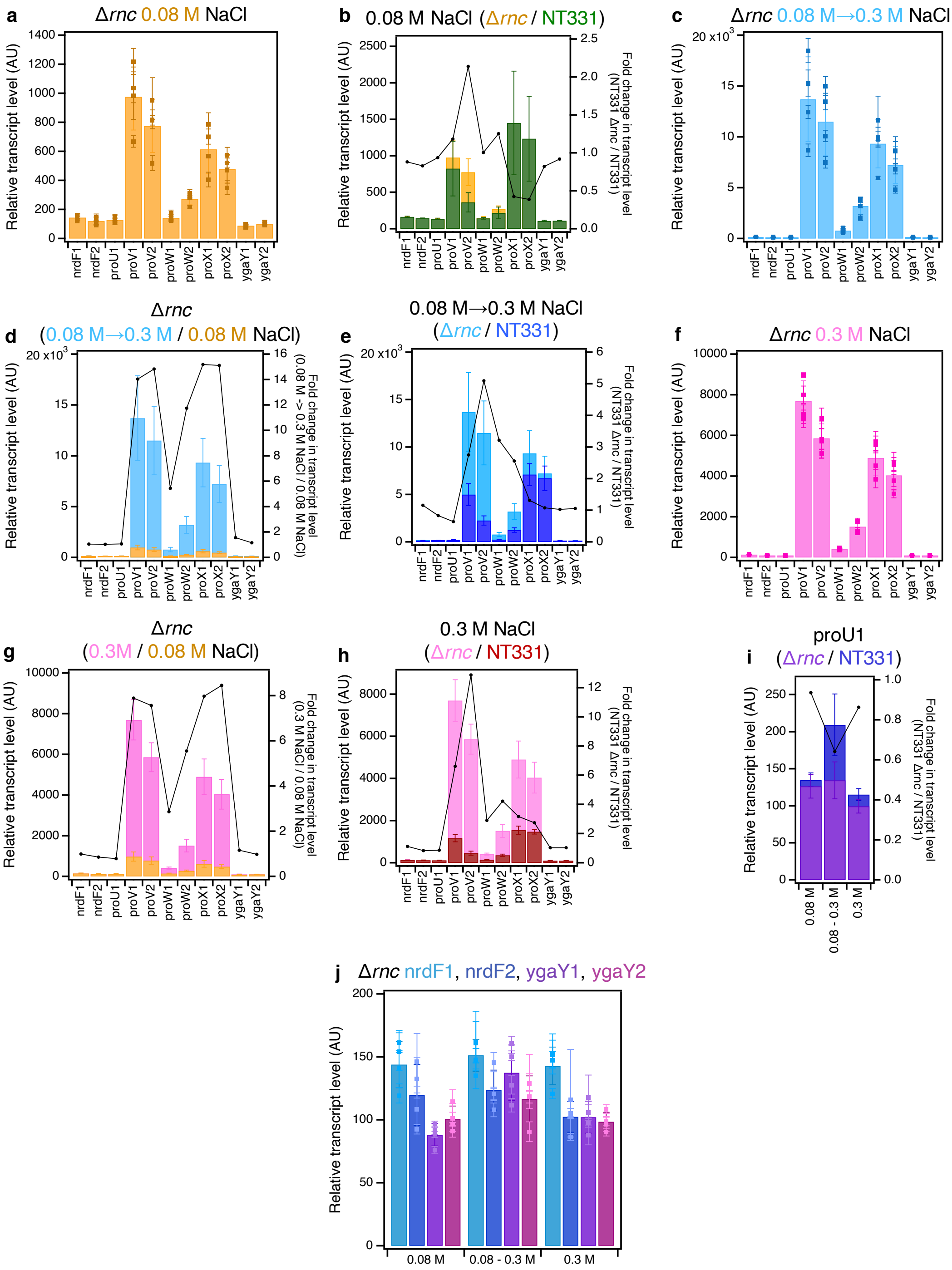

Supplementary Fig. 8: The RT-qPCR profile of *proVWX* and its flanking regions in NT331 Δ*rnc* during (a) exponential growth at 0.08 M NaCl, and the fold change in transcript levels of the amplicons (b) compared to NT331 growing exponentially at 0.08 M NaCl. The RT-qPCR profile of *proVWX* and its flanking regions in NT331 Δ*rnc* upon (c) a hyperosmotic shock from 0.08 M NaCl to 0.3 M NaCl, and the fold change in transcript levels of the amplicons in comparison to (d) NT331 Δ*rnc* growing exponentially at 0.08 M NaCl, and (e) NT331 following a hyperosmotic shock. The RT-qPCR profile of *proVWX* and its flanking regions in NT331 Δ*rnc* during (f) exponential growth at 0.3 M NaCl, and the fold change in transcript leve;s of the amplicons relative to (g) NT331 Δ*rnc* growing exponentially at 0.08 M NaCl, and (h) NT331 growing exponentially at 0.3 M NaCl. (i) The fold change in the relative transcript levels of the *proU*1 amplicon between NT331 Δ*rnc* and NT331. (j) The relative transcript level in NT331 Δ*rnc* at amplicons flanking *proVWX* during exponential growth at 0.08 M NaCl, following a hyperosmotic shock, and during exponential growth at 0.3 M NaCl. Y-axes: All bar graphs and data points with error bars show relative transcript levels in arbitrary units and are plotted on the left y-axis. Plots without error bars show fold-change in transcript level and correspond to the right y-axis. Internal control: *hcaT*. See also Fig. 4. Data (Supplementary Fig. 8a-j) are presented as mean values +/- standard devation. Dot plots (Supplementary Fig. 8a, 8c, 8f, and 8j): n=3 technical replicates of a biologically independent culture. Bar graphs (Supplementary Fig. 8a-j): n=4 biologically independent cultures. Source data are provided as a Source Data file.

Escherichia coli **cells show global differences in the chromosome contact profiles during growth at different osmolarity conditions.**

The binding of NAPs to DNA is sensitive to environmental conditions such as pH, temperature, and osmolarity [19–28]. Consequently, changes to the ambient growth conditions of bacteria are reflected in an altered NAP binding profile of the chromosome, and hence, in the three-dimensional chromosome organization. We first used Hi-C, a high-throughput chromosome conformation capture technique, to examine the global differences in the chromosome contact profiles of MG1655 Δ*endA* (NT331) (Supplementary Fig. 10 and 11) during growth in a low-salt (0.08 M NaCl) medium, following a hyperosmotic shock (0.08 M -> 0.3 M NaCl), and in a high-salt (0.3 M NaCl) medium (Supplementary Fig. 9). To improve the signal-to-noise ratio of the chromosome contact maps, *E. coli* cells were permeabilised with methanol prior to formaldehyde fixation, and the proximity ligation step of Hi-C was performed using the insoluble fraction of digested, cross-linked chromatin (Supplementary Fig. 14 and 15) [29].

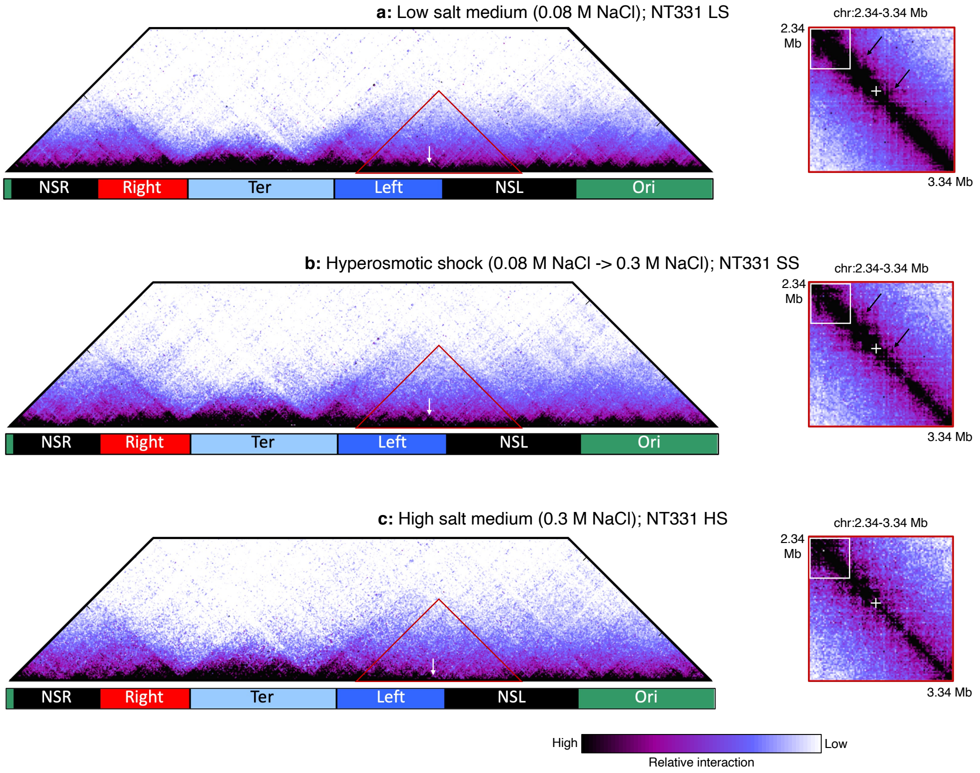

Supplementary Fig. 9: *Escherichia coli* cells show global differences in chromosome contact profiles during growth at different osmolarity conditions. (a) & (b) The chromosome around *proVWX* (marked with a red triangle in the left panels, shown in the right panels) decompacts locally when *E. coli* cells growing exponentially in a low salt medium (a) are subjected to a hyperosmotic shock (b). The position of *proVWX* is marked with white arrows in the left panels and with white '+' marks in the right panels. The local chromosome maintains features of the finer chromosome organization after a hyperosmotic shock, such as loops (black arrows, right panels) and the arrow-like structure at 2.37 Mb (white squares, right panels). (c) Upon adaptation to hyperosmotic stress represented as exponential growth in a high salt medium, loci either show decompaction compared to growth in a low salt medium such as the chromatin encompassing, and positioned locally downstream of *proVWX*, or show a stronger compaction, for example, the region encompassing the arrow-like structure at 2.37 Mb (white square, right panels). Organism: *Escherichia coli* MG1655 Δ*endA* (NT331); 3C-based study: Hi-C; Resolution: 10 kb; Fixation conditions: 80% cold methanol for 10 minutes followed by 3% formaldehyde for 1 hour (Supplementary Fig. 14 and 15); Restriction enzyme: PsuI (ThermoFisher Scientific); Fractionation: Yes.

The NT331 chromosome contact maps show global chromosomal rearrangements in response to osmolarity. The rearrangements are also observed in the vicinity of the osmosensitive *proVWX* operon (Supplementary Fig. 9). When *E. coli* cells in a low-salt medium are subjected to a hyperosmotic shock, the local chromosome at *proVWX* decompacts while maintaining features of the finer chromosome organization, such as loops (marked with black arrows, Supplementary Fig. 9a-b, right panels) and the arrow-like structure at 2.37 Mb (marked with white squares, Supplementary Fig. 9a-b, right panels). In a high salt environment – a condition that reflects the adaptation of *E. coli* to higher osmolarity following a hyperosmotic shock – loci either

decompact further such as the chromatin encompassing, and positioned locally downstream of *proVWX*, or show a stronger compaction compared to growth in a low-salt medium, for instance, the region encompassing the arrow-like structure at 2.37 Mb (marked with a white square, Supplementary Fig. 9c, right panel).

Degradation of *Escherichia coli* chromatin during 3C-based library preparation is overcome by the deletion of *endA*.

The *Escherichia coli* K-12 MG1655 strain closely resembles the genetic make-up of archetype *E. coli* and is used as a reference for genome-wide studies of NAP-binding profiles and transcription. The strain is, therefore, the optimal choice to study the interplay between three-dimensional chromatin organisation, NAP distribution, and gene expression. However, chromatin extracted from MG1655 underwent considerable degradation during the initial steps of 3-C and Hi-C library preparation. The degradation was not observed during the lysis and solubilisation steps [4] that were carried out in a buffer with 1.0 mM EDTA but occurred extensively once the cell lysate was diluted in a restriction digestion mix with a final EDTA concentration of 0.1 mM (Supplementary Fig. 10a). The dependence of chromatin degradation on the concentration of EDTA, and hence, the availability of divalent ions implied that the degradation was enzymatic.

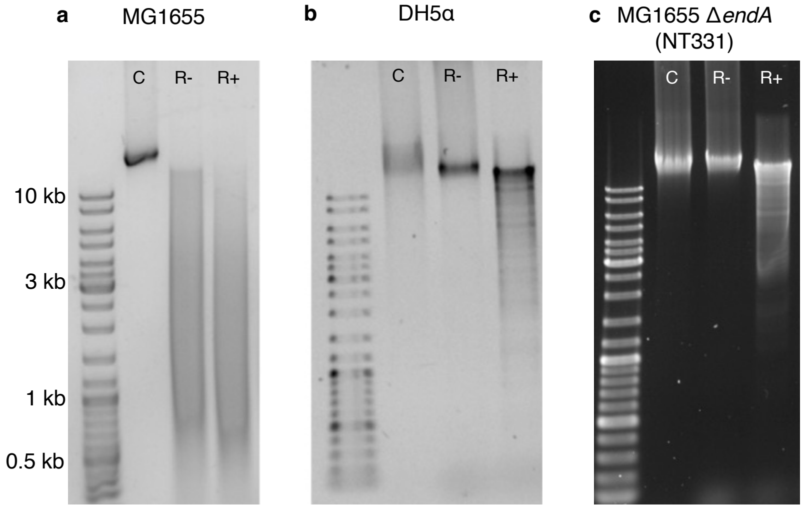

Supplementary Fig. 10: Degradation of *Escherichia coli* chromatin during 3C-based library preparation is overcome by *endA* deletion. Lane C: Chromatin preparation in 1X TE (EDTA concentration: 1.0 mM); Lane R-: Chromatin preparation after a 3-hour incubation in 1X restriction digestion buffer (EDTA concentration: 0.1 mM); Lane R+: Chromatin preparation after a 3-hour treatment with 0.4 U/µL of BglII in 1X restriction digestion buffer (EDTA concentration: 0.1 mM). (a) MG1655 chromatin undergoes extensive degradation in a restriction digestion buffer with a final EDTA concentration of 0.1 mM. The degradation is observed as a smear in lanes R- and R+. Similar degradation is not observed in *endA* knock-out strains (b) DH5α and (c) NT331, where extracted chromatin (lane C) still runs as a heavy >10 kb band after a 3-hour incubation in a buffer with 0.1 mM EDTA (lane R-). Fixed chromatin extracted from the *endA*$^-$ strains can be digested by restriction enzymes (shown: BglII).

Endonuclease-I is a DNA-specific nuclease localised in the periplasm [5] that digests dsDNA in a sequence independent manner. It is responsible for the low quality of plasmid DNA preparations from *endA*$^+$ *E. coli* strains [6,7]. To investigate whether the enzyme also contributes to the degradation of chromatin in lysates of formaldehyde-treated cells, the stability of fixed chromatin extracted from DH5α – an *endA*$^-$ strain of *E. coli* [7] – during the initial steps of chromosome conformation capture was tested. Agarose gel electrophoresis showed that DH5α chromatin preparations do not degrade in the restriction digestion buffer with 0.1 mM EDTA (Supplementary Fig. 10b). Attempts to thermally denature endonuclease-I and overcome chromatin degradation were not pursued extensively since the conditions that reliably decreased degradation also promote reverse cross-linking of the chromatin and thereby interfere with proximity ligation in later steps of 3C-based protocols (Supplementary Fig. 11).

Therefore, MG1655 Δ*endA* (henceforth referred to as NT331) was generated using the λ-red recombinase mediated gene replacement strategy [8,9]. Chromatin preparations from fixed NT331 do not degrade when incubated in a buffer with a low concentration of EDTA (Supplementary Fig. 10c). Thus, all 3C-based experiments, and the associated RT-qPCR studies, were carried out in a Δ*endA* background.

Using higher concentrations of formaldehyde for fixation, for instance, 7%, also reduces chromatin degradation [10,11]. Nevertheless, we preferred to use *endA-* strains for our 3C-based studies since this reduces the potential for introducing artefacts that are associated with using high concentrations of fixatives. It also provides a wider window to finetune fixation conditions.

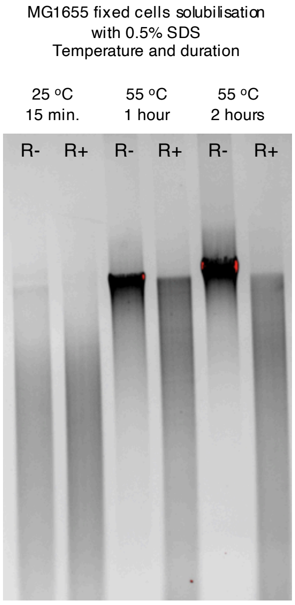

Supplementary Fig. 11: Cell solubilisation conditions that reliably decrease degradation of MG1655 chromatin in low [EDTA] buffers also promote de-crosslinking of formaldehyde-fixed chromatin. Raising the temperature and increasing the duration of 0.5% SDS treatment during lysis and solubilisation of fixed cells increases the stability of the extracted chromatin in low [EDTA] buffer.

RNA preparations do not show detectable genomic DNA contamination.

In compliance with MIQE guidelines [12], all RNA preparations were tested for genomic DNA contamination. ~100 ng of RNA with and without RNase treatment (RNase+ and RNase-, respectively) were visualised on a 1.2% agarose gel pre-stained with 1X GelRed (Sigma-Aldrich). The absence of a nucleic acid signal in the RNase+ wells shows the absence of detectable genomic DNA contamination (Supplementary Fig. 12).

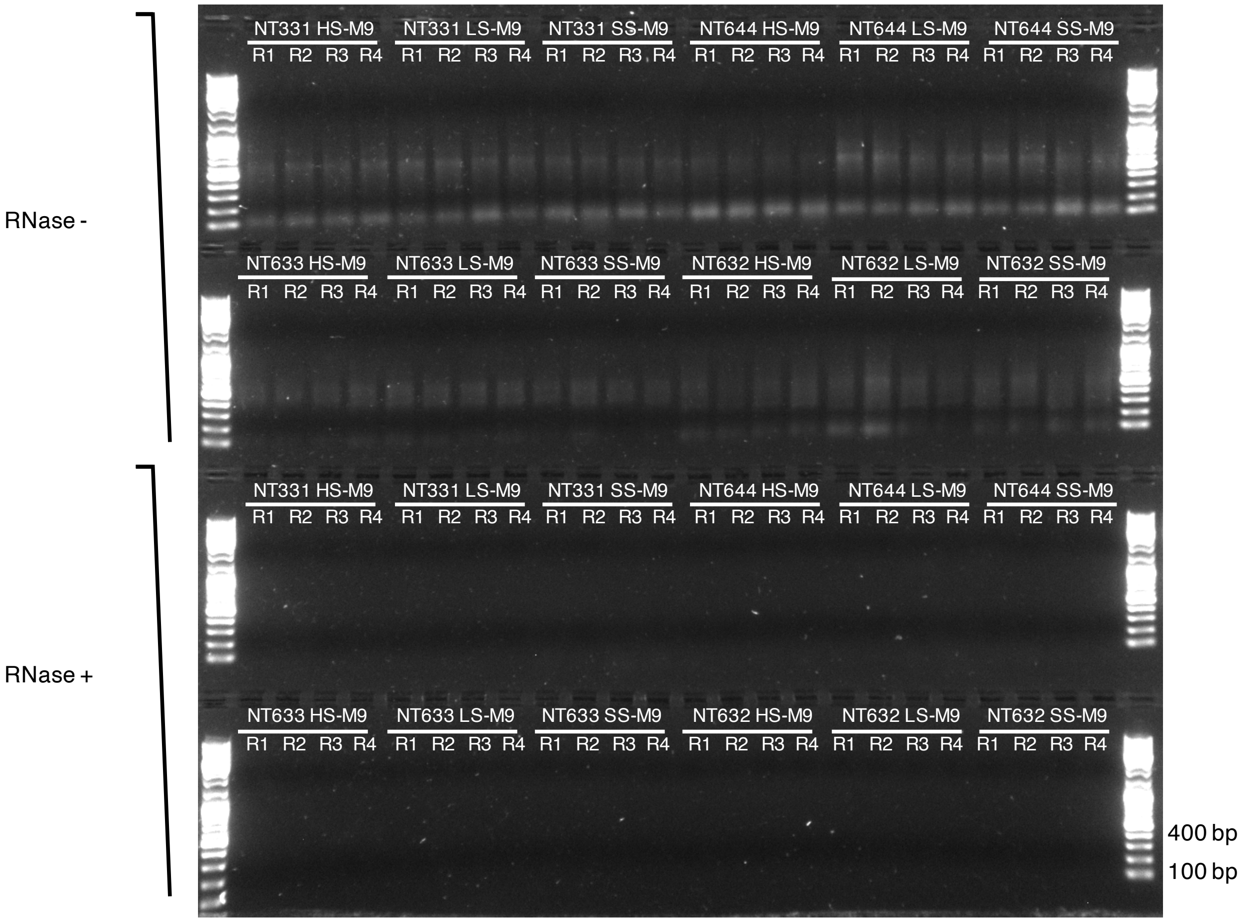

Supplementary Fig. 12: DNA contamination assessment of RNA preparations. RNA preparations of NT331, NT644, NT633, and NT632 (Table 1) show no nucleic acid signal upon treatment with RNase. An uncropped scan of the gel has been provided in the Source Data at the end of this document.

The specificity of primer pairs used for RT-qPCR studies was determined with melting curve analysis and Sanger sequencing of the amplified product.

In compliance with MIQE guidelines [12], the specificity of primer pairs was experimentally determined by Sanger sequencing (BaseClear B.V., Leiden, The Netherlands) of the amplified products (Supplementary Data 1B and 2). The melting curve profiles and the melting temperature (Tm) of the sequenced amplicons (Supplementary Fig. 13) were used to gauge the specificity of amplification in RT-qPCR experiments.

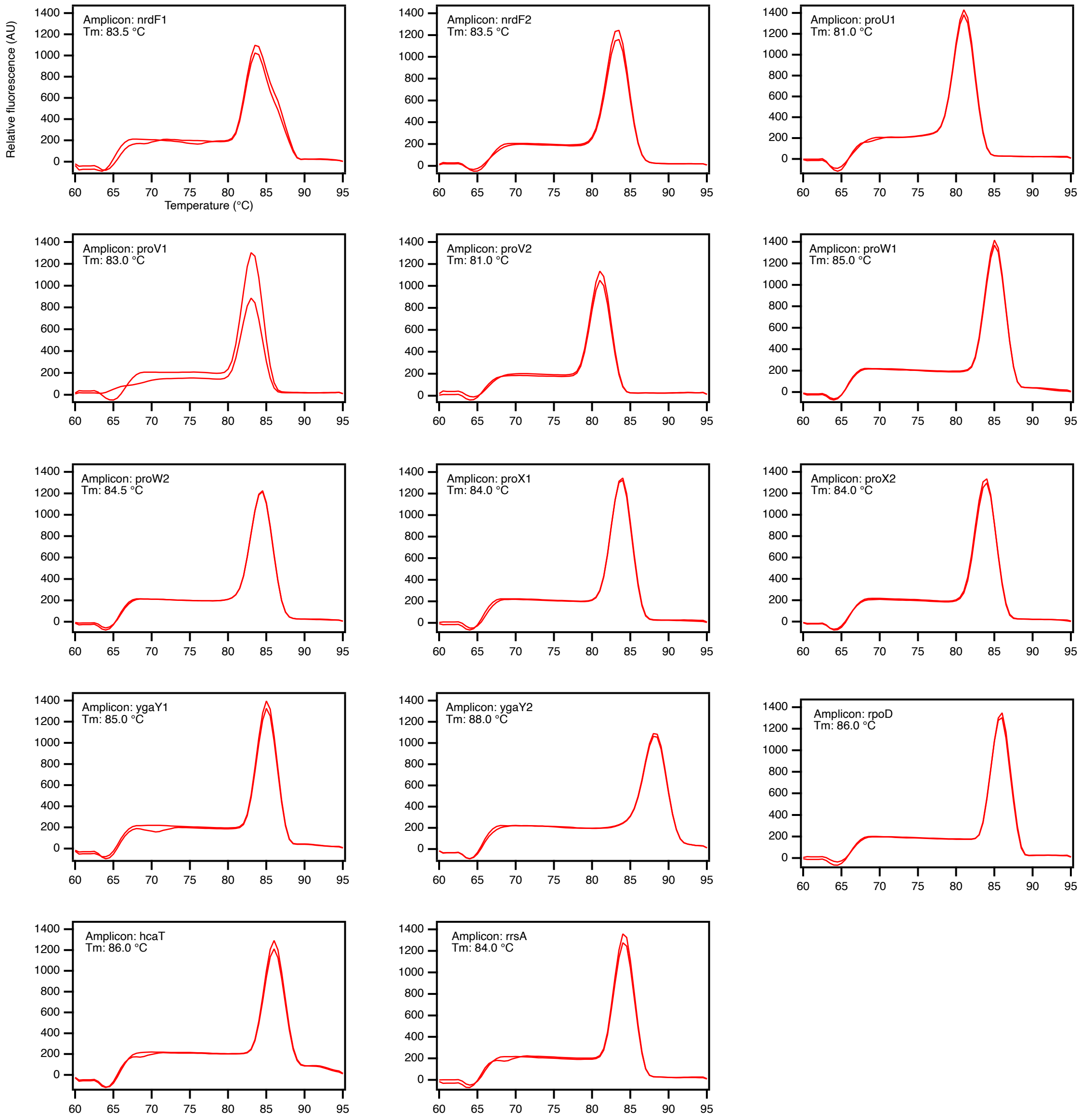

Supplementary Fig. 13: RT-qPCR amplicon melting curves. The specificity of amplification in RT-qPCR reactions was determined from the melting curve of the fragment amplified in each well. The melting curve and the melting temperature (Tm) of the amplicons reported on in this manuscript are shown here. The sequences of the fragments used for this experiment were verified with Sanger sequencing (Supplementary Data 1B and 2). Source data are provided as a Source Data file.

Proximity ligation with the insoluble fraction of digested, cross-linked chromatin, and methanol permeabilization of *Escherichia coli* prior to formaldehyde fixation improves the signal-to-noise ratio in chromosome contact maps.

Hi-C libraries of NT331 fixed with 3% formaldehyde have a low signal-to-noise ratio (Supplementary Fig. 14a), indicating inefficient formaldehyde-mediated cross-linking. Taking an earlier report of 3C-based studies in *E. coli* [10] into account, we raised the concentration of formaldehyde for fixation from 3% to 7%. However, the change did not contribute to a significant improvement in chromosome contact maps (Supplementary Fig. 15).

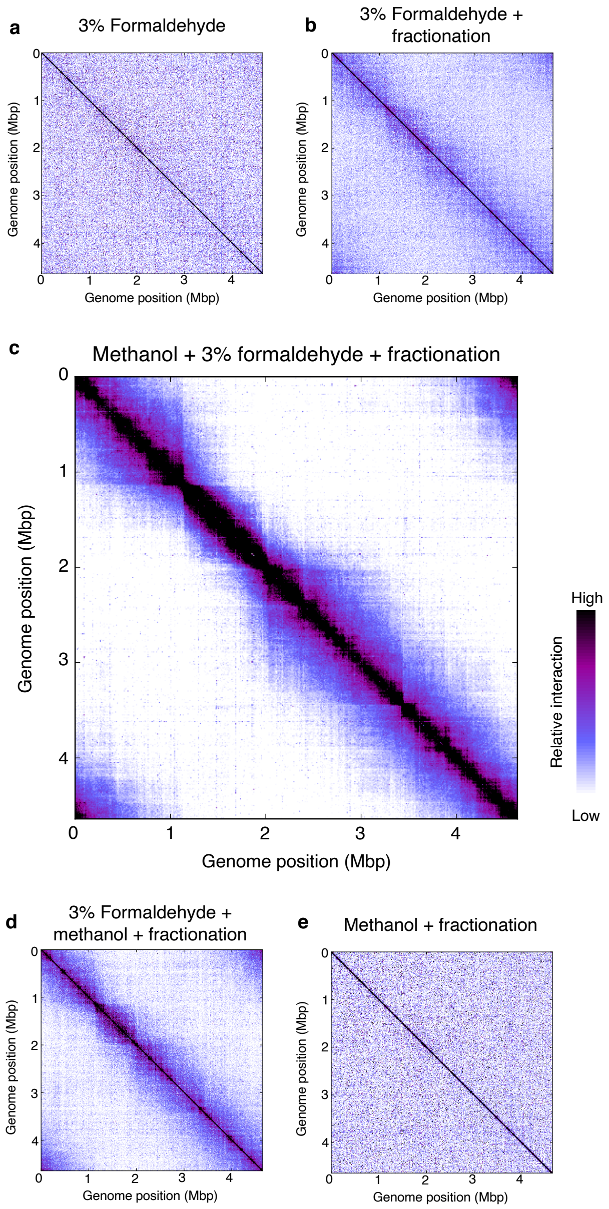

Supplementary Fig. 14: Proximity ligation with the insoluble fraction of digested, cross-linked chromatin, and methanol permeabilization of *Escherichia coli* prior to formaldehyde fixation improves the signal-to-noise ratio in chromosome contact maps. (a) Hi-C libraries prepared from cells fixed with 3% formaldehyde have a low signal-to-noise ratio. (b) The signal-to-noise ratio is improved by proximity ligation with the insoluble fraction of digested, cross-linked chromatin and (c) further improved by methanol permeabilization of *E. coli* cells prior to formaldehyde fixation. (d) Only a marginal improvement in the signal-to-noise ratio of chromosome contact maps is observed when methanol treatment is performed after formaldehyde fixation. (e) *E. coli* cells permeabilised with methanol but not fixed with formaldehyde cannot be used to map chromosome structure. Organism: *Escherichia coli* MG1655 Δ*endA* (NT331); 3C-based study: Hi-C; Resolution: 10 kb; Growth conditions: LB medium, 37 °C, exponential phase; Fixation conditions: a and b: 3% formaldehyde, 1 hour, c: 80% cold methanol for 10 minutes followed by 3% formaldehyde for 1 hour, d: 3% formaldehyde for 1 hour followed by 80% cold methanol for 10 minutes, e: 80% cold methanol for 10 minutes; Restriction enzyme: PsuI (ThermoFisher Scientific); Fractionation: a: No, b-e: Yes.

A low signal-to-noise ratio in chromosome contact maps may arise from ligation between freely moving, non-crosslinked DNA molecules [13]. This effect can be overcome by fractionating the digested, cross-linked chromatin into its supernatant, and pellet fractions by centrifugation [13,14]. Cross-linked DNA-protein complexes are enriched in the pellet and freely moving DNA molecules in the supernatant. This allows contact maps with a high signal-to-noise ratio to be generated when proximity ligation is carried out with only the pellet fraction [13,14]. In agreement with previous observations in E14.5 mouse embryos [14], *Saccharomyces cerevisiae* [13], and *S. pombe* [13], incorporating fractionation and using only the pellet fraction for proximity ligation improved the signal-to-noise ratio of the *Escherichia coli* NT331 contact map (Supplementary Fig. 14b). Fractionation was also incorporated in 3C-based studies of *Bacillus subtilis* [15].

Formaldehyde is a standard fixative in histology and (immuno-)histochemical studies where it is used either alone, or in combination with methanol [16,17]. Methanol dissolves lipids from cell membranes and coagulates proteins, thus, simultaneously permeabilizing and fixing histological preparations [17,18]. We extrapolated this to *E. coli*, and permeabilized the cells with 80% methanol prior to formaldehyde fixation. This treatment led to a significant improvement in the signal-to-noise ratio of chromosome contact maps (Supplementary Fig. 14c). Contact maps of chromosomes fixed in this manner are qualitatively indistinguishable from those fixed with 7% formaldehyde as in REF[10]. In comparison, only a weak improvement in the signal-to-noise ratio of chromosome contact maps was observed when methanol treatment was performed after formaldehyde fixation (Supplementary Fig. 14d). Methanol treatment alone could not be used to study chromosome conformation (Supplementary Fig. 14e).

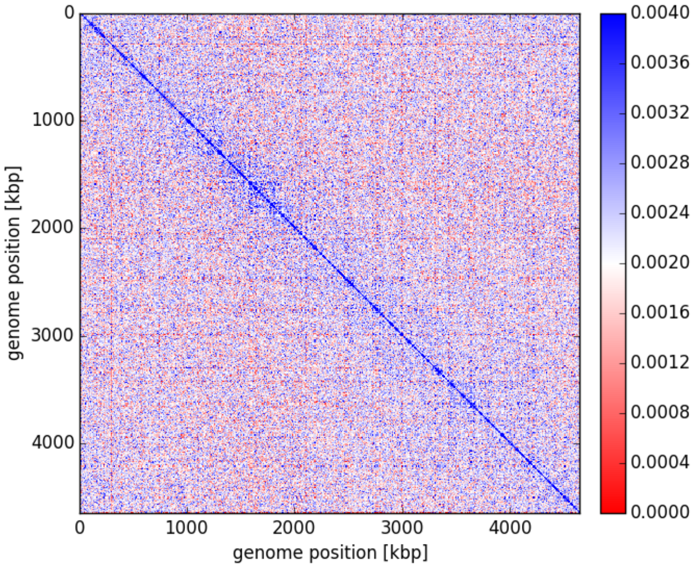

Supplementary Fig. 15: Fixation of *E. coli* cells with higher concentrations of formaldehyde does not significantly improve the signal-to-noise ratio in chromosome contact maps. Chromosome contact maps of *E. coli* cells fixed using 7% formaldehyde do not show a significant improvement in the signal-to-noise ratio compared to maps generated from *E. coli* cells fixed with 3% formaldehyde (Supplementary Fig. 14a). Organism: *Escherichia coli* MG1655 Δ*endA* (NT331); 3C-based study: 3C-Seq; Growth conditions: LB medium, 37 °C, exponential phase; Fixation conditions: 7% formaldehyde, 1 hour; Restriction enzyme: HpaII (NEB); Fractionation: Not performed.

Supplementary tables

Supplementary Table 1: Relative transcript levels of amplicons within and flanking the *proVWX* operon in NT331. Internal control: *rpoD*

Amplicon	NT331 0.08 M NaCl	NT331 Hyperosmotic shock	NT331 0.3 M NaCl
nrdF1	81.76±8.58	80.31±6.67	85.26±11.58
nrdF2	72.65±11.31	90.84±2.98	82.50±9.49
proU1	67.60±7.90	128.77±27.04	76.72±7.43
proV1	402.16±150.56	3047.84±637.44	773.91±89.45
proV2	177.21±50.60	1376.57±238.83	304.88±73.74
proW1	71.52±11.27	148.65±13.91	94.40±12.63
proW2	106.71±31.29	766.58±137.55	237.86±27.42
proX1	699.64±279.30	4365.00±726.11	1025.76±84.89
proX2	598.77±226.02	4125.85±795.50	979.43±65.30
ygaY1	53.92±8.70	82.41±8.39	65.80±4.53
ygaY2	54.98±5.93	67.00±7.56	63.53±6.03

Supplementary Table 2: Relative transcript levels of amplicons within and flanking the *proVWX* operon in NT331. Internal control: *hcaT*

Amplicon	NT331 0.08 M NaCl	NT331 Hyperosmotic shock	NT331 0.3 M NaCl
nrdF1	163.32±8.61	130.70±10.10	127.36±7.29
nrdF2	144.37±4.35	148.28±12.99	123.56±9.44
proU1	135.01±9.47	209.23±41.67	115.02±7.97
proV1	825.06±374.20	4982.62±1159.38	1165.23±168.78
proV2	362.27±132.65	2255.64±482.54	455.49±98.55
proW1	142.41±11.69	242.24±25.71	141.03±7.61
proW2	217.29±79.75	1250.22±241.01	358.28±53.15
proX1	1449.73±710.92	7101.04±1153.99	1545.50±192.71
proX2	1234.74±581.37	6713.35±1288.12	1471.58±109.99
ygaY1	107.28±9.05	133.79±6.59	99.17±11.73
ygaY2	109.74±4.21	110.35±6.71	95.51±10.66

Supplementary Table 3: Relative transcript levels of amplicons within and flanking the *proVWX* operon in NT644. Internal control: *rpoD*

Amplicon	NT644 0.08 M NaCl	NT644 Hyperosmotic shock	NT644 0.3 M NaCl
nrdF1	88.27±5.12	91.17±17.10	72.81±4.94
nrdF2	68.34±8.97	71.09±5.69	78.61±5.15
proU1	74.79±15.20	169.18±76.33	81.69±3.77
proV1	2428.61±1177.42	4032.33±868.78	734.54±175.17
proV2	796.10±341.97	1618.03±183.18	264.97±35.89
proW1	141.29±21.40	254.71±41.71	97.67±14.01
proW2	496.96±194.66	1081.64±127.27	193.62±19.97
proX1	4717.25±2145.92	11245.74±2835.34	1287.77±115.42
proX2	3378.13±1791.64	7965.02±2759.61	1128.00±210.66
ygaY1	48.54±9.77	63.73±8.35	55.60±5.71
ygaY2	55.56±4.41	55.20±3.46	56.70±3.78

Supplementary Table 4: Relative transcript levels of amplicons within and flanking the *proVWX* operon in NT644. Internal control: *hcaT*

Amplicon	NT644 0.08 M NaCl	NT644 Hyperosmotic shock	NT644 0.3 M NaCl
nrdF1	162.28±24.75	145.43±15.62	123.93±10.31
nrdF2	124.49±13.55	119.73±10.06	133.98±13.44
proU1	139.59±46.30	297.39±165.21	139.22±12.29
proV1	4632.45±2942.73	6948.43±2328.41	1279.27±465.74
proV2	1508.89±862.37	2748.93±522.37	458.92±116.22
proW1	260.22±58.78	436.10±120.87	166.54±27.97
proW2	927.22±448.16	1826.21±278.49	330.78±47.92
proX1	8984.84±5455.14	19333.58±6401.31	2193.00±238.20
proX2	6489.04±4488.92	13841.45±5963.81	1907.92±247.87
ygaY1	89.55±22.12	107.22±13.53	94.29±5.71
ygaY2	101.75±12.28	93.06±7.94	96.49±7.63

Supplementary Table 5: Relative transcript levels of amplicons within and flanking the *proVWX* operon in NT331 Δ*stpA* (NT633). Internal control: *rpoD*

Amplicon	NT633 0.08 M NaCl	NT633 Hyperosmotic shock	NT633 0.3 M NaCl
nrdF1	46.25±2.53	79.10±7.59	54.52±11.33
nrdF2	29.79±8.16	58.43±11.39	43.00±13.54
proU1	19.98±5.58	120.60±5.80	45.17±8.90
proV1	1808.97±533.26	26048.07±3254.38	8119.52±931.61
proV2	1248.25±267.61	20226.51±3164.81	5557.57±880.61
proW1	67.33±7.67	1100.01±205.74	296.37±28.02
proW2	410.52±136.89	7934.30±1992.17	1963.72±512.42
proX1	2708.60±468.16	28803.66±6405.98	8606.95±1628.96
proX2	1739.62±233.27	16464.36±3391.55	5773.60±938.28
ygaY1	28.40±5.41	313.55±46.54	65.85±7.87
ygaY2	26.85±5.16	111.52±9.33	41.30±8.68

Supplementary Table 6: Relative transcript levels of amplicons within and flanking the *proVWX* operon in NT331 Δ*stpA* (NT633). Internal control: *hcaT*

Amplicon	NT633 0.08 M NaCl	NT633 Hyperosmotic shock	NT633 0.3 M NaCl
nrdF1	125.97±10.43	193.56±14.99	150.77±23.88
nrdF2	79.80±17.35	142.70±24.42	116.26±17.29
proU1	53.39±10.33	295.26±6.41	124.22±8.26
proV1	5052.77±2083.77	63812.32±8172.40	23504.72±7658.21
proV2	3465.18±1140.43	49570.11±8135.08	16194.99±5930.20
proW1	182.25±3.84	2697.16±538.20	850.69±237.53
proW2	1147.82±517.95	19404.52±4834.93	5847.17±2784.05
proX1	7488.12±2115.74	70727.28±16904.09	25217.37±9585.28
proX2	4800.36±1181.15	40439.05±9029.73	16724.05±5495.00
ygaY1	76.42±7.41	768.07±117.33	184.66±34.12
ygaY2	72.25±6.94	273.21±23.27	113.45±9.30

Supplementary Table 7: Relative transcript levels of amplicons within and flanking the *proVWX* operon in NT331 Δ*rnc* (NT632). Internal control: *rpoD*

Amplicon	NT632 0.08 M NaCl	NT632 Hyperosmotic shock	NT632 0.3 M NaCl
nrdF1	83.09±6.94	90.11±13.79	82.23±7.34
nrdF2	69.94±18.13	73.93±16.09	59.03±7.40
proU1	73.48±12.17	80.54±21.69	57.72±10.85
proV1	559.26±96.36	8023.76±2095.41	4434.21±530.35
proV2	444.98±85.59	6715.55±1571.80	3394.43±544.03
proW1	83.09±14.12	453.45±83.01	239.58±53.78
proW2	157.33±19.22	1876.76±403.74	872.33±175.58
proX1	356.34±107.62	5511.92±1414.30	2818.13±460.38
proX2	277.14±63.50	4227.19±823.92	2319.72±359.24
ygaY1	51.17±6.31	80.78±7.12	59.70±13.03
ygaY2	58.37±4.84	68.84±9.64	57.59±11.98

Supplementary Table 8: Relative transcript levels of amplicons within and flanking the *proVWX* operon in NT331 Δ*rnc* (NT632). Internal control: *hcaT*

Amplicon	NT632 0.08 M NaCl	NT632 Hyperosmotic shock	NT632 0.3 M NaCl
nrdF1	143.94±18.40	151.32±12.66	142.88±14.94
nrdF2	119.79±24.13	123.63±15.83	102.38±12.24
proU1	126.33±15.92	134.26±24.93	99.27±9.15
proV1	975.93±228.81	13700.86±4167.98	7699.76±991.49
proV2	775.22±183.41	11494.71±3369.42	5862.75±710.33
proW1	143.06±22.42	779.19±221.60	409.41±48.64
proW2	272.42±40.24	3200.35±827.86	1512.10±312.42
proX1	614.23±166.78	9329.09±2384.86	4897.48±877.88
proX2	477.58±95.71	7217.89±1818.43	4037.97±734.01
ygaY1	88.16±8.82	137.41±22.07	102.15±12.71
ygaY2	100.83±10.11	116.59±18.27	98.49±7.54

Supplementary Table 9: Relative interaction frequency of proU3_NlaIII with fragments within and flanking the *proVWX* operon in NT331. Cross-linking control: proU3_NlaIII-proU6_NlaIII

Interaction fragment	NT331 0.08 M NaCl	NT331 Hyperosmotic shock	NT331 0.3 M NaCl
proU17_NlaIII	38.08±6.78	34.77±0.90	42.64±2.06
proU16_NlaIII	48.38±9.33	40.35±1.19	47.88±4.10
proU13_NlaIII	110.91±13.79	100.89±2.51	122.07±8.30
proU1_NlaIII	72.52±5.73	63.02±2.23	76.42±2.95
proU2_NlaIII	155.34±17.46	123.83±8.94	141.17±8.83
proU4_NlaIII	81.39±8.21	81.91±3.90	88.48±5.82
proU5_NlaIII	140.22±5.60	130.55±1.25	136.32±4.47
proU6_NlaIII	100.00	100.00	100.00
proU7_NlaIII	95.91±5.04	98.59±8.97	100.39±5.33
proU8_NlaIII	35.10±0.43	37.75±1.66	39.88±1.64
proU9_NlaIII	28.22±1.86	27.64±1.48	31.33±1.02
proU10_NlaIII	47.43±3.70	41.79±3.08	47.15±3.09
proU11_NlaIII	51.98±4.69	43.40±3.16	48.07±1.88
proU12_NlaIII	47.34±3.15	45.05±4.16	52.05±2.86
proU14_NlaIII	28.11±2.64	23.39±1.73	28.24±1.13
proU15_NlaIII	32.75±4.00	26.70±2.68	33.50±2.23

Supplementary Table 10: Relative interaction frequency of proU3_NlaIII with fragments within and flanking the *proVWX* operon in NT644. Cross-linking control: proU3_NlaIII-proU6_NlaIII

Interaction fragment	NT644 0.08 M NaCl	NT644 Hyperosmotic shock	NT644 0.3 M NaCl
proU17_NlaIII	28.37±3.12	34.64±4.01	38.61±2.76
proU16_NlaIII	39.09±3.45	45.97±7.36	43.15±2.83
proU13_NlaIII	86.13±8.13	103.83±15.69	106.67±5.20
proU1_NlaIII	65.71±8.06	66.14±4.66	71.05±4.12
proU2_NlaIII	127.74±6.05	133.70±20.05	126.79±12.71
proU4_NlaIII	101.16±4.66	108.39±18.94	107.01±8.58
proU5_NlaIII	132.61±3.97	139.70±6.27	136.66±5.42
proU6_NlaIII	100.00	100.00	100.00
proU7_NlaIII	89.51±4.09	100.21±3.76	101.13±10.40
proU8_NlaIII	34.05±2.66	38.65±3.39	38.61±1.17
proU9_NlaIII	27.78±1.36	27.27±0.51	28.85±2.32
proU10_NlaIII	39.96±1.85	43.84±1.30	40.87±2.19
proU11_NlaIII	32.25±3.46	35.52±1.18	35.34±2.77
proU12_NlaIII	39.88±3.71	43.69±1.12	45.18±2.85
proU14_NlaIII	25.45±1.86	23.33±2.09	24.59±2.06
proU15_NlaIII	31.44±2.07	30.54±2.76	30.73±2.91

Supplementary Table 11: Relative interaction frequency of proU3_NlaIII with fragments within and flanking the *proVWX* operon in NT331 treated with rifampicin. Cross-linking control: proU3_NlaIII-proU6_NlaIII

Interaction fragment	NT331 Rif 0.08 M NaCl	NT331 Rif Hyperosmotic shock	NT331 Rif 0.3 M NaCl
proU17_NlaIII	59.23±6.49	59.56±2.51	51.78±2.11
proU16_NlaIII	71.31±6.79	74.98±0.40	65.28±2.37
proU13_NlaIII	124.05±4.71	120.12±1.60	121.87±4.86
proU1_NlaIII	100.04±3.78	98.79±2.27	98.35±2.44
proU2_NlaIII	134.81±23.76	121.26±3.79	143.55±9.33
proU4_NlaIII	62.69±3.61	64.38±2.87	70.21±3.61
proU5_NlaIII	123.92±7.68	113.29±2.40	125.70±5.62
proU6_NlaIII	96.23±5.09	90.39±0.30	94.54±1.18
proU7_NlaIII	100.00	100.00	100.00
proU8_NlaIII	34.91±2.19	35.72±3.19	33.77±1.54
proU9_NlaIII	35.29±3.12	39.63±0.89	37.23±1.77
proU10_NlaIII	48.25±2.36	49.68±2.11	53.36±3.83
proU11_NlaIII	53.53±3.55	53.08±1.06	54.71±2.44
proU12_NlaIII	46.52±0.74	48.09±0.61	47.58±1.29

proU14_NlaIII	30.02±2.04	32.06±0.60	29.61±1.22
proU15_NlaIII	34.95±1.40	35.13±0.71	31.08±1.21

Supplementary Table 12: RT-qPCR reaction composition per well. Modified from the manufacturer's protocol provided with the Luna® Universal One-Step RT-qPCR Kit.

Component	Volume in a 10.0 µL reaction	Final concentration
‡2X Luna Universal One-Step Reaction Mix (NEB)	5.0 µL	1X
‡20X Luna WarmStart® RT Enzyme Mix (NEB)	0.5 µL	1X
‡100 µM forward primer (SI 1A and SI 1B)	0.04 µL	0.4 µM
‡100 µM reverse primer (SI 1A and SI 1B)	0.04 µL	0.4 µM
‡Nuclease free water (Gibco®, life Technologies™)	3.92 µL	N/A
~20 ng/µL RNA sample	0.5 µL	~10 ng

‡ These components were added to each reaction as a master mix.

Supplementary Table 13: RT-qPCR thermal cycling parameters. As per the manufacturer's instructions provided with the Luna® Universal One-Step RT-qPCR Kit.

No.	Step	Temperature	Duration (mm:ss)	Cycles
1	Reverse transcription	55 °C	10:00	1
2	Initial denaturation	95 °C	01:00	1
3	Denaturation	95 °C	00:10	
4	Annealing/extension	60 °C	00:30	45
5	Plate read (SYBR/FAM channel)	N/A	N/A	
6	Melt	60 °C	0:31	1
7	Ramp	60 °C	0:01	
		+ 0.5 °C/cycle	+ 0:01/0.5 °C	70
8	Plate read (SYBR/FAM channel)	N/A	N/A	

Supplementary Table 14: 3C-qPCR reaction composition per well. Modified from the manufacturer's protocol provided with the PrimeTime® Gene Expression Master Mix.

Component	Volume per reaction	Final concentration
‡2X PrimeTime® Gene Expression Master Mix (IDT)	5.0 µL	1X
‡100 µM TaqMan probe (IDT)	0.015 µL	0.15 µM
‡100 µM Constant primer (SI 1C)	0.05 µL	0.5 µM
‡100 µM Test primer (SI 1C)	0.05 µL	0.5 µM
~0.2 ng/µL 3C library	5.0 µL	~1 ng

‡ These components were added to each reaction as a master mix.

Supplementary Table 15: 3C-qPCR thermal cycling parameters. As per the manufacturer's instructions provided with the PrimeTime® Gene Expression Master Mix.

No.	Step	Temperature	Duration (mm:ss)	Cycles
1	Polymerase activation	95 °C	03:00	1
3	Denaturation	95 °C	00:15	
4	Annealing/extension	60 °C	01:00	45
5	Plate read (SYBR/FAM channel)	N/A	N/A	

Supplementary References:

1. Uyar, E. *et al.* Differential binding profiles of StpA in wild-type and h-ns mutant cells: a comparative analysis of cooperative partners by chromatin immunoprecipitation-microarray analysis. *J Bacteriol* **191**, 2388–91 (2009).
2. Lucht, J. M., Dersch, P., Kempf, B. & Bremer, E. Interactions of the nucleoid-associated DNA-binding protein H-NS with the regulatory region of the osmotically controlled proU operon of Escherichia coli. *J Biol Chem* **269**, 6578–8 (1994).
3. Bouffartigues, E., Buckle, M., Badaut, C., Travers, A. & Rimsky, S. H-NS cooperative binding to high-affinity sites in a regulatory element results in transcriptional silencing. *Nat Struct Mol Biol* **14**, 441–8 (2007).
4. Crémazy, F. G. *et al.* Determination of the 3D Genome Organization of Bacteria Using Hi-C. *Methods Mol Biol* **1837**, 3–18 (2018).
5. Cordonnier, C. & Bernardi, G. Localization of E. coli endonuclease I. *Biochem Biophys Res Commun* **20**, 555–9 (1965).
6. Taylor, R. G., Walker, D. C. & McInnes, R. R. E. coli host strains significantly affect the quality of small scale plasmid DNA preparations used for sequencing. *Nucleic Acids Res* **21**, 1677–8 (1993).
7. Hanahan, D. Techniques for transformation of Escherichia coli. *D.M. Glover (ed.), DNA cloning Vol. 1 A practical approach* **1.**, 109–135 (1995).
8. Datsenko, K. A. & Wanner, B. L. One-step inactivation of chromosomal genes in Escherichia coli K-12 using PCR products. *Proc Natl Acad Sci U S A* **97**, 6640–5 (2000).
9. Kolmsee, T. & Hengge, R. Rare codons play a positive role in the expression of the stationary phase sigma factor RpoS (σ(S)) in Escherichia coli. *RNA Biol* **8**, 913–21 (2011).
10. Lioy, V. S. *et al.* Multiscale Structuring of the E. coli Chromosome by Nucleoid-Associated and Condensin Proteins. *Cell* **172**, 771-783.e18 (2018).
11. Lioy, V. S. & Boccard, F. Conformational Studies of Bacterial Chromosomes by High-Throughput Sequencing Methods. *Methods Enzymol* **612**, 25–45 (2018).
12. Bustin, S. A. *et al.* The MIQE guidelines: minimum information for publication of quantitative real-time PCR experiments. *Clin Chem* **55**, 611–22 (2009).
13. Hsieh, T.-H. S., Fudenberg, G., Goloborodko, A. & Rando, O. J. Micro-C XL: assaying chromosome conformation from the nucleosome to the entire genome. *Nat Methods* **13**, 1009–1011 (2016).
14. Gavrilov, A. A. *et al.* Disclosure of a structural milieu for the proximity ligation reveals the elusive nature of an active chromatin hub. *Nucleic Acids Res* **41**, 3563–75 (2013).
15. Marbouty, M. *et al.* Condensin- and Replication-Mediated Bacterial Chromosome Folding and Origin Condensation Revealed by Hi-C and Super-resolution Imaging. *Mol Cell* **59**, 588–602 (2015).
16. Malatesta, M. Histological and Histochemical Methods - Theory and practice. *European Journal of Histochemistry* **60**, (2016).
17. Jamur, M. C. & Oliver, C. Cell fixatives for immunostaining. *Methods Mol Biol* **588**, 55–61 (2010).
18. Jamur, M. C. & Oliver, C. Permeabilization of cell membranes. *Methods Mol Biol* **588**, 63–6 (2010).
19. Dame, R. T. The role of nucleoid-associated proteins in the organization and compaction of bacterial chromatin. *Mol Microbiol* **56**, 858–870 (2005).
20. Dame, R. T., Kalmykowa, O. J. & Grainger, D. C. Chromosomal macrodomains and associated proteins: implications for DNA organization and replication in gram negative bacteria. *PLoS Genet* **7**, e1002123 (2011).
21. Dame, R. T. & Tark-Dame, M. Bacterial chromatin: converging views at different scales. *Curr Opin Cell Biol* **40**, 60–65 (2016).
22. Dillon, S. C. & Dorman, C. J. Bacterial nucleoid-associated proteins, nucleoid structure and gene expression. *Nat Rev Microbiol* **8**, 185–95 (2010).
23. Dorman, C. J. Genome architecture and global gene regulation in bacteria: making progress towards a unified model? *Nat Rev Microbiol* **11**, 349–55 (2013).
24. Rimsky, S. & Travers, A. Pervasive regulation of nucleoid structure and function by nucleoid-associated proteins. *Curr Opin Microbiol* **14**, 136–141 (2011).
25. Noom, M. C., Navarre, W. W., Oshima, T., Wuite, G. J. L. & Dame, R. T. H-NS promotes looped domain formation in the bacterial chromosome. *Curr Biol* **17**, R913-4 (2007).
26. van der Valk, R. A., Vreede, J., Crémazy, F. & Dame, R. T. Genomic looping: a key principle of chromatin organization. *J Mol Microbiol Biotechnol* **24**, 344–59 (2014).
27. Luijsterburg, M. S., White, M. F., van Driel, R. & Dame, R. T. The major architects of chromatin: architectural proteins in bacteria, archaea and eukaryotes. *Crit Rev Biochem Mol Biol* **43**, 393–418 (2008).
28. Dame, R. T., Rashid, F.-Z. M. & Grainger, D. C. Chromosome organization in bacteria: mechanistic insights into genome structure and function. *Nat Rev Genet* **21**, 227–242 (2020).
29. Rashid, F.-Z. M., Detmar, L. & Dame, R. T. Chromosome Conformation Capture in Bacteria and Archaea. *Methods Mol Biol* **2516**, 1–28 (2022).

Source Data:

Supplementary Fig. 5:

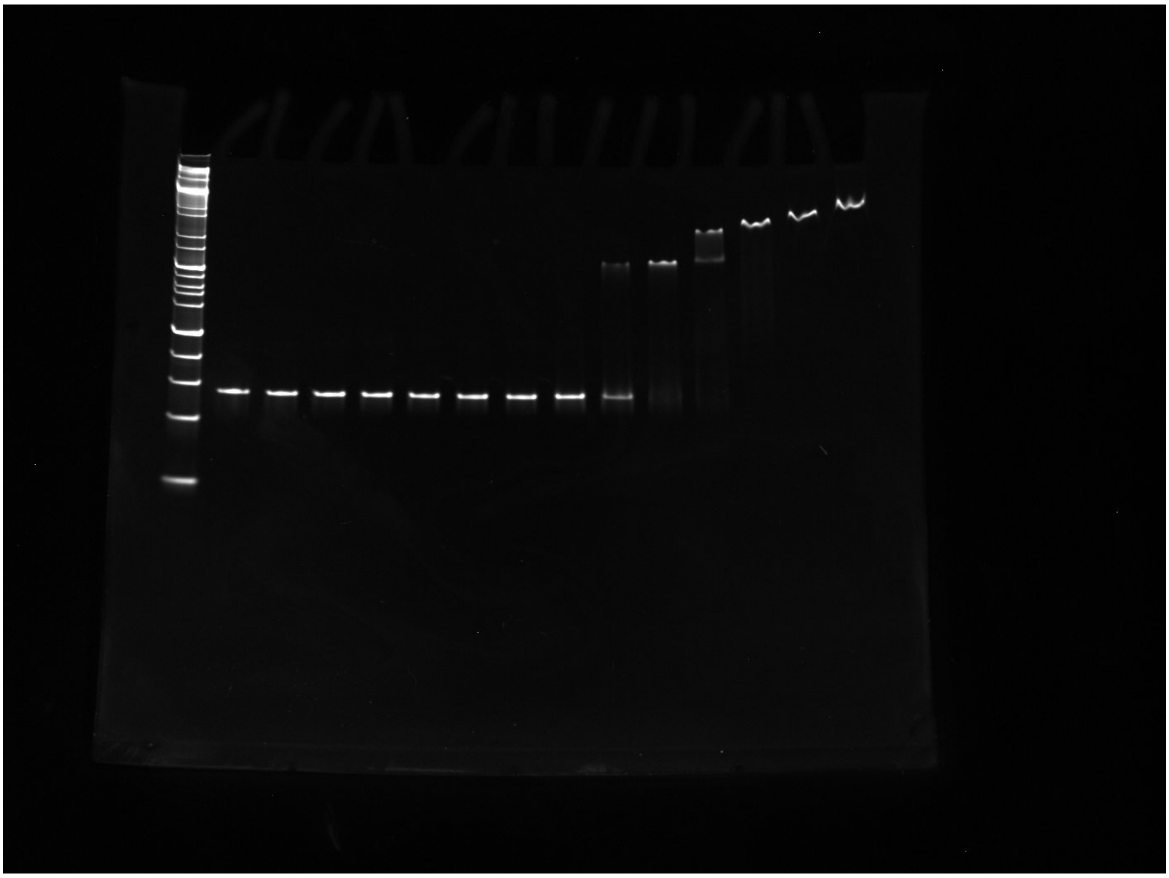

Source data for Supplementary Fig. 5 (top) – DRE^mut^

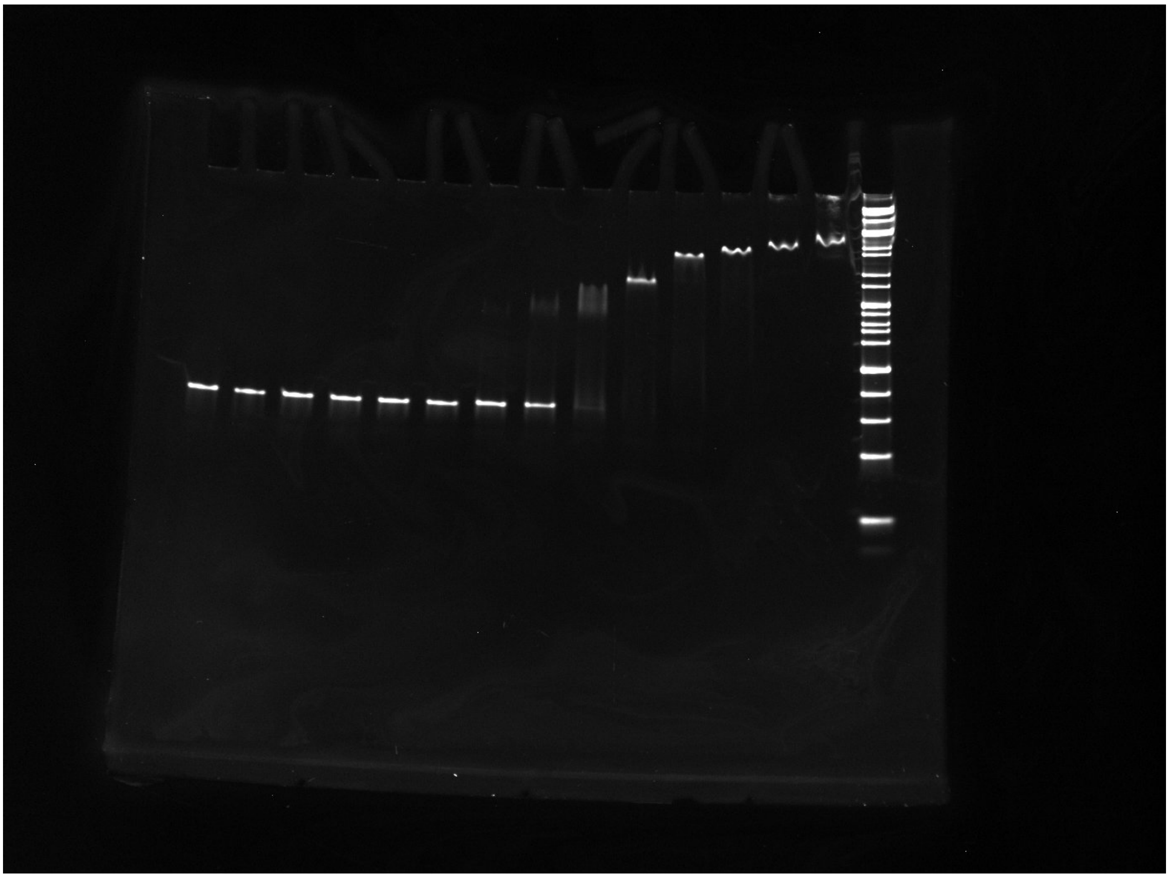

Source data for Supplementary Fig. 5 (bottom) – DRE^wt^

Supporting source data for Supplementary Fig. 5:
EMSA gels corresponding to DRE^{mut}

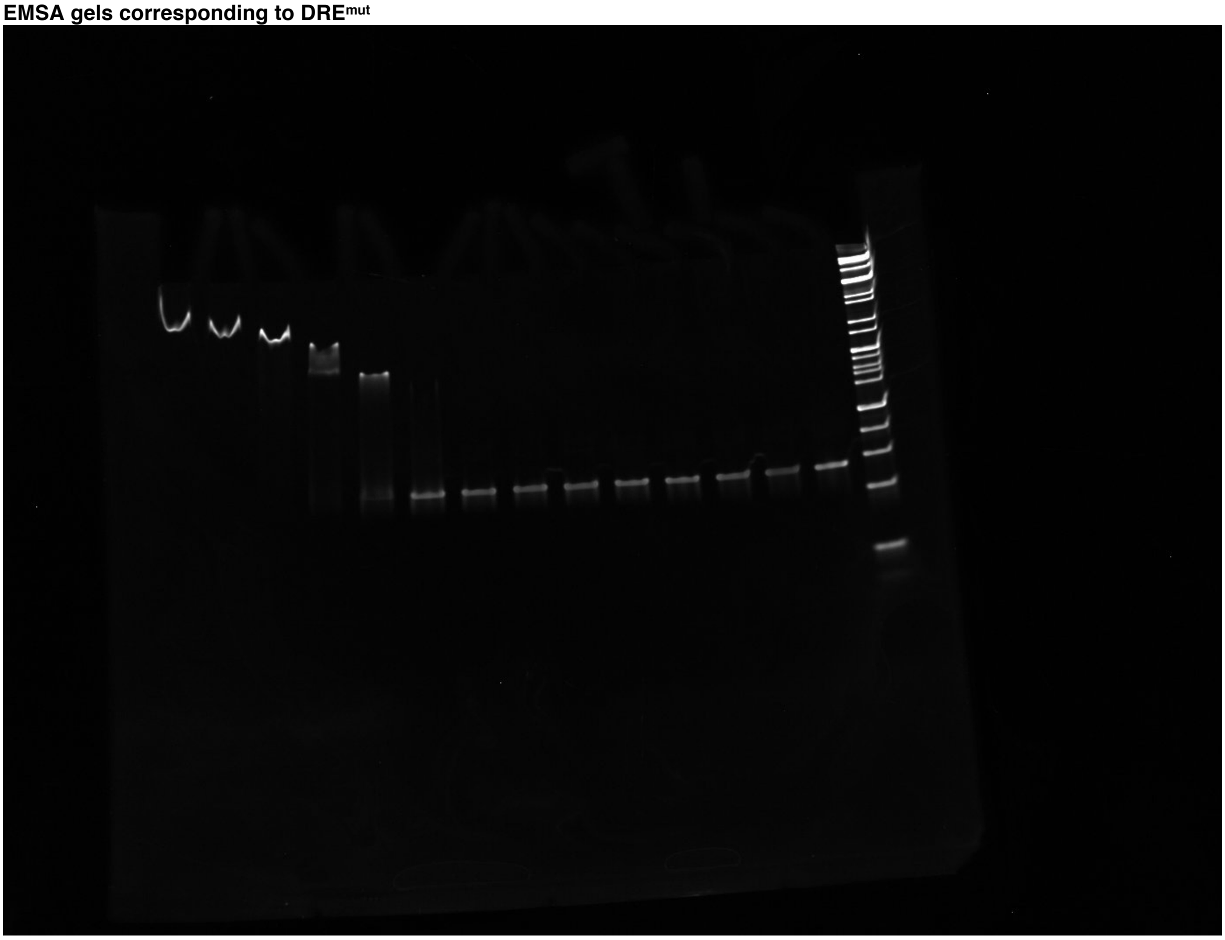

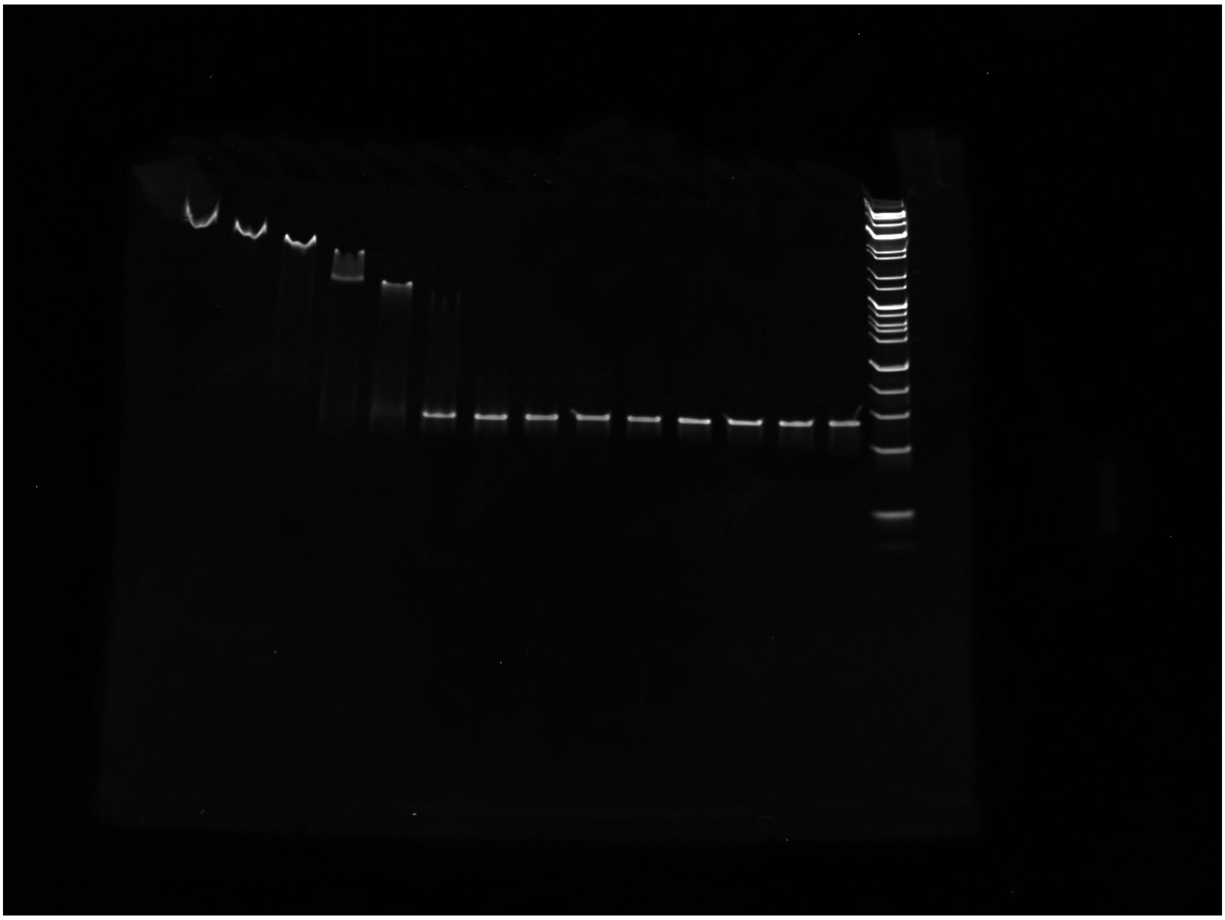

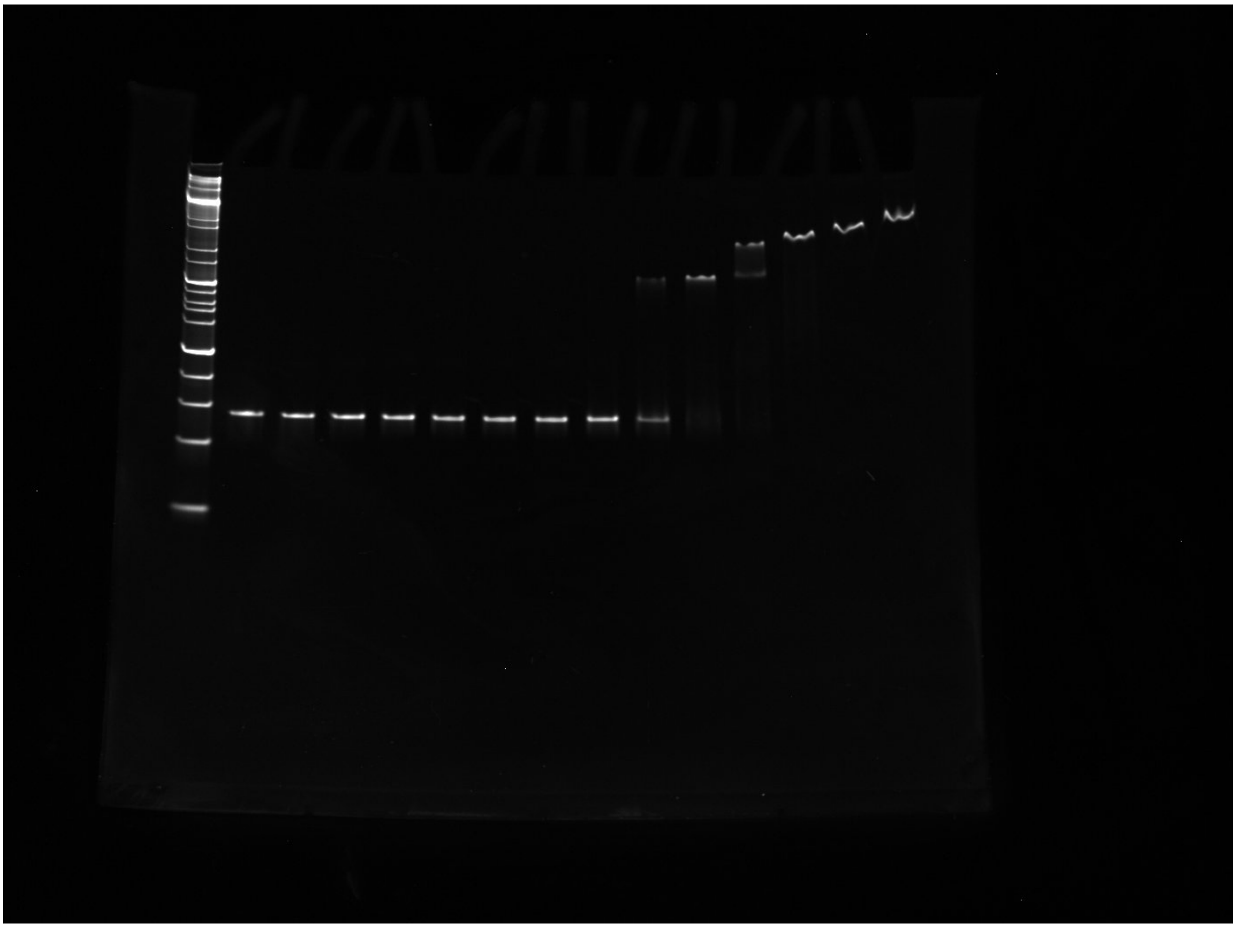

EMSA gels corresponding to DRE^{wt}

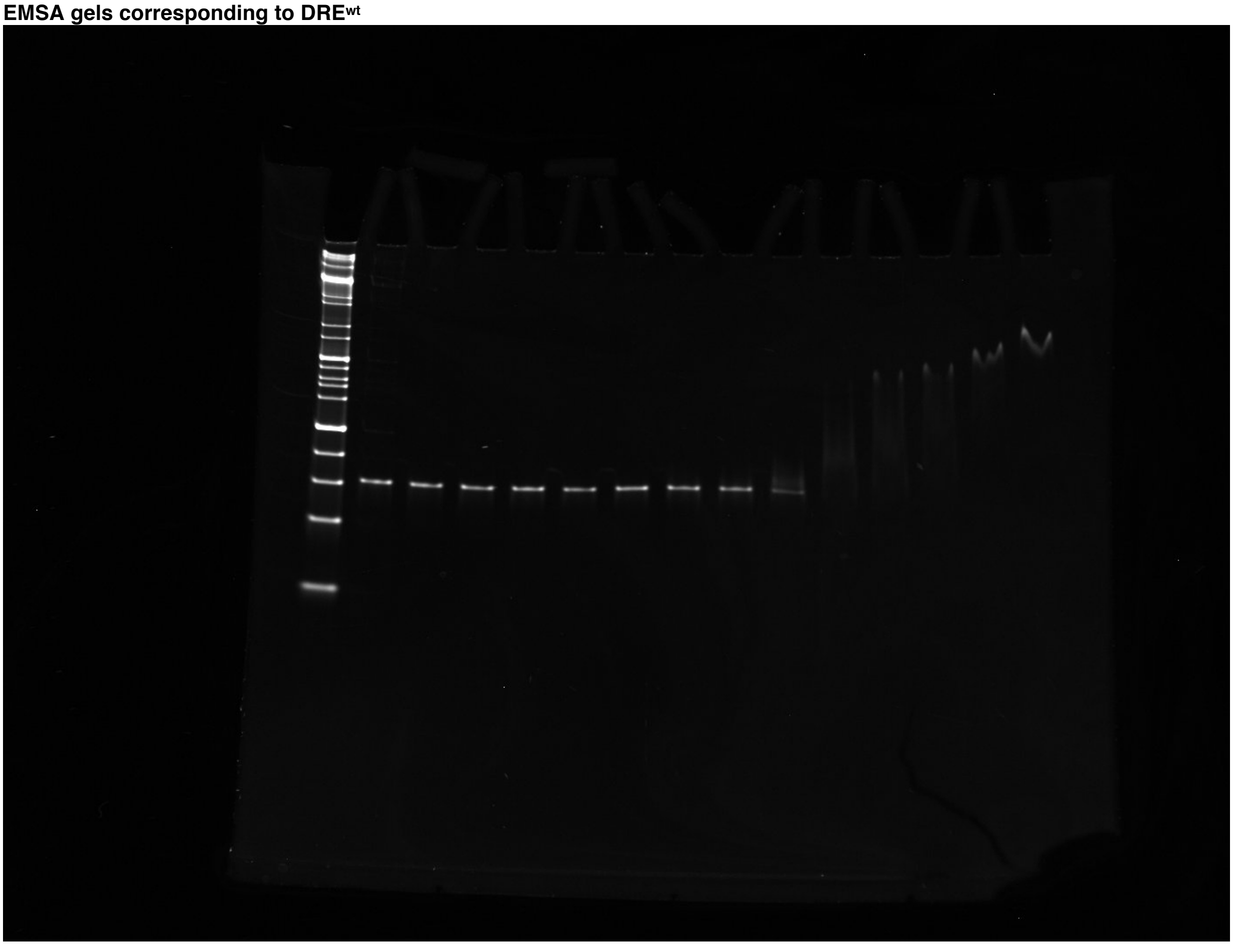

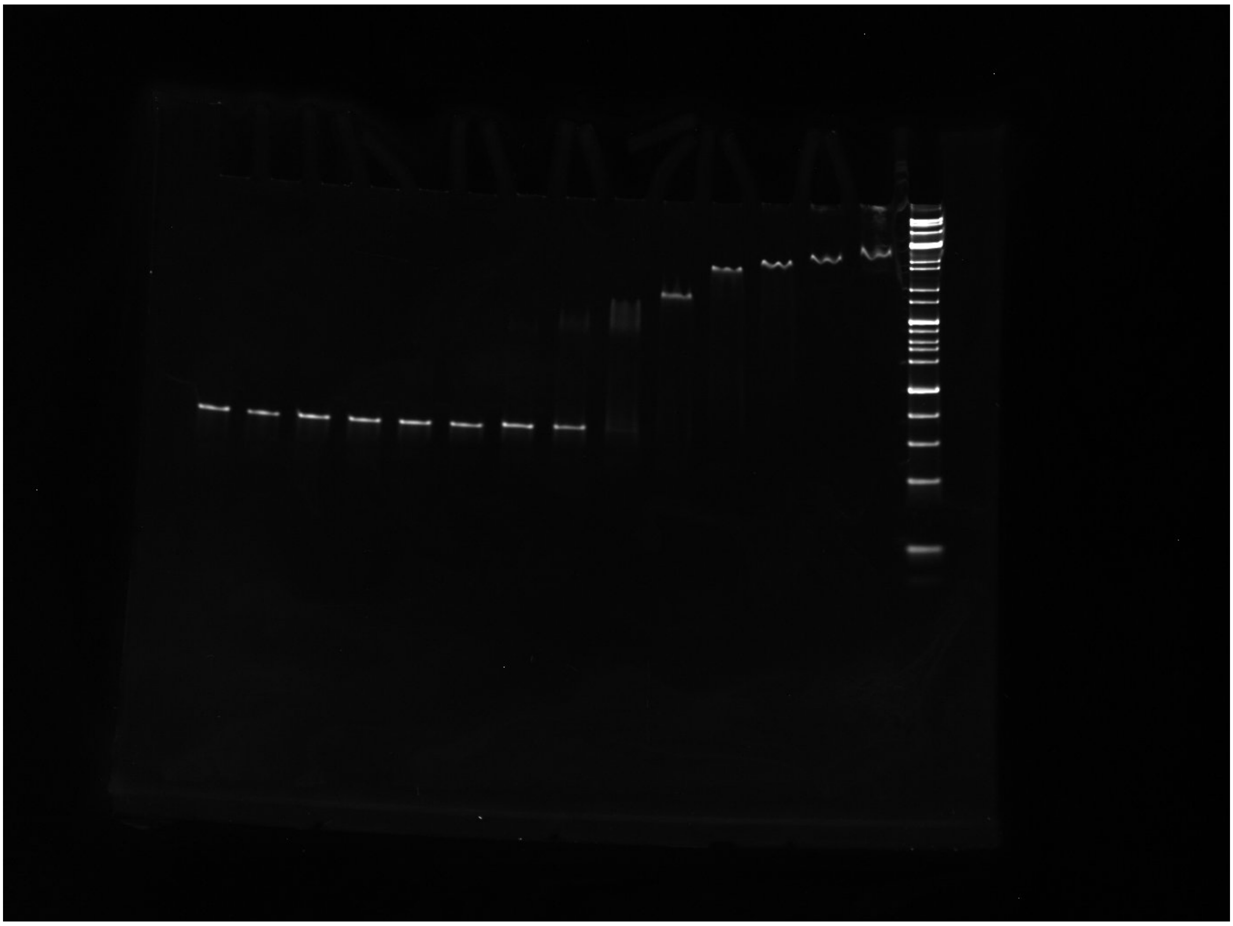

Supplementary Fig. 12:

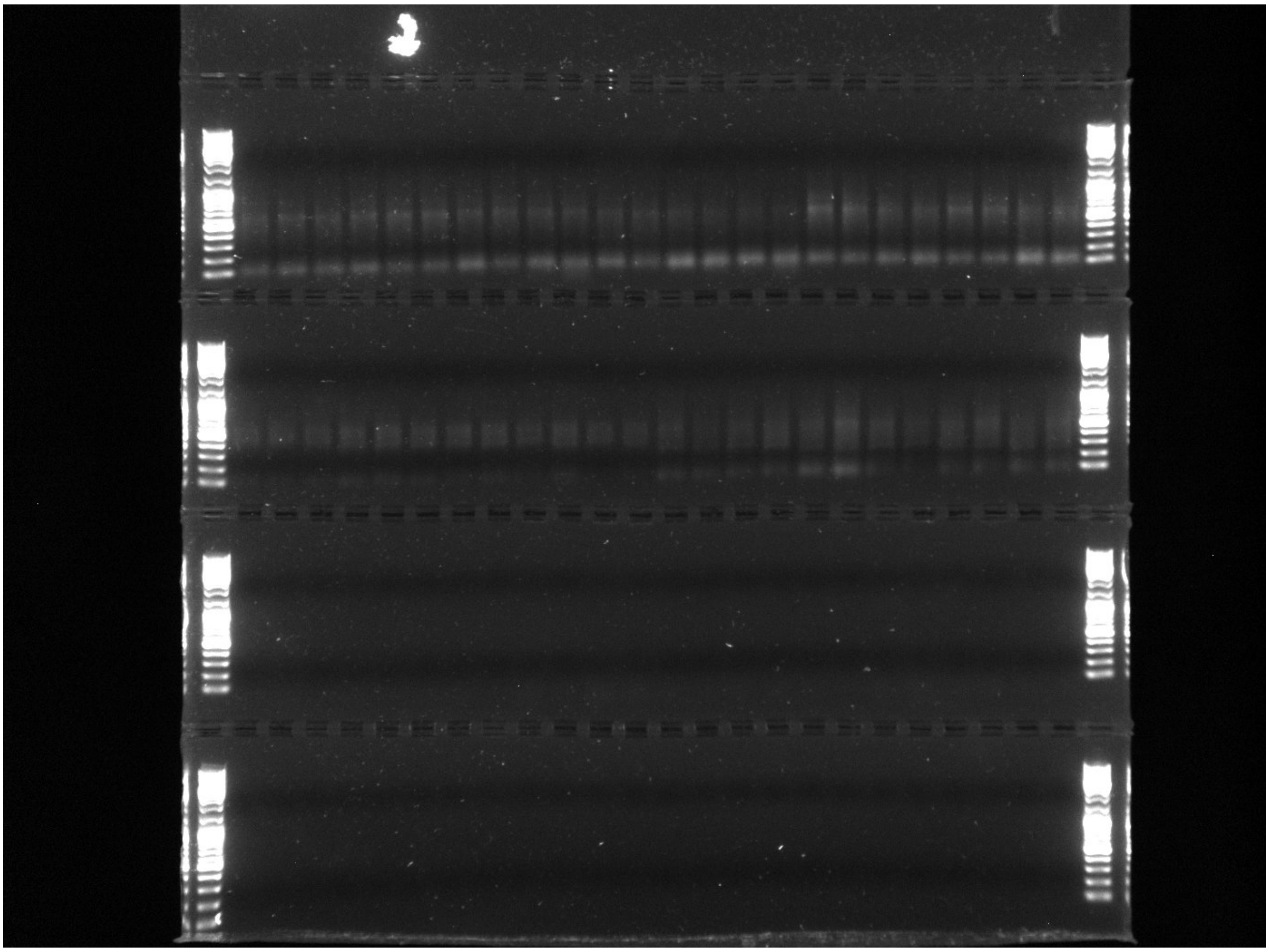

Source data for Supplementary Fig. 12
