# Supplementary material for: The environmentally-regulated interplay between local three-dimensional chromatin organisation and transcription of proVWX in E. coli
Source: Nat Commun. 2023 Nov 17;14:7478. doi: 10.1038/s41467-023-43322-y (PMC10656529; doi:10.1038/s41467-023-43322-y)
Supplement: Supplementary file 3 — Description of Additional Supplementary Files [file 41467_2023_43322_MOESM3_ESM.pdf]

Description of Additional Supplementary Files

File Name: Supplementary Data 1
Description: Analysed data files of the *proVWX* operon. A) Overview of the *proVWX* operon. B) Overview of RT-qPCR primers and amplicons, tests of RT-qPCR primer specificity, measurements of RNA yield and purity, and RNA inhibition testing. C) RT-qPCR analysis of NT331 (MG1655 Δ*endA*) and NT644 (NT331 *proVWX* DRE mutant). D) RT-qPCR analysis of NT331 Δ*stpA* and NT331 Δ*rnc*. E) NT331 3C libraries inhibition test, NT644 3C libraries inhibition test, Rifampicin-treated NT331 (NT331-Rif) 3C libraries inhibition test, measurements of 3C library yields. F) Overview of 3C-qPCR primers, TaqMan probes, and amplicons. G) 3C-qPCR analysis of NT331 and NT644. H) 3C-qPCR analysis of NT331-Rif.

File Name: Supplementary Data 2
Description: Sanger sequencing files of RT-qPCR amplicons.

File Name: Supplementary Data 3
Description: RT-qPCR raw data files and exported *.csv or *.xls or *.xlsx files for NT331 and NT644.

File Name: Supplementary Data 4
Description: RT-qPCR raw data files and exported *.csv or *.xls or *.xlsx files for NT331 Δ*stpA* and NT331 Δ*rnc*.

File Name: Supplementary Data 5
Description: 3C-qPCR raw data files and exported *.csv or *.xls or *.xlsx files for NT331 and NT644.

File Name: Supplementary Data 6
Description: 3C-qPCR raw data files and exported *.csv or *.xls or *.xlsx files for NT331-Rif

File Name: Supplementary Data 7
Description: 3C-qPCR No Template Control (NTC) raw data files and exported *.csv, *.xls, or *.xlsx files.
