# Supplementary material for: The environmentally-regulated interplay between local three-dimensional chromatin organisation and transcription of proVWX in E. coli
Source: Nat Commun. 2023 Nov 17;14:7478. doi: 10.1038/s41467-023-43322-y (PMC10656529; doi:10.1038/s41467-023-43322-y)
Supplement: Supplementary file 4 — Supplementary Data 1 [file 41467_2023_43322_MOESM4_ESM.zip › Supplementary Data 1/Supplementary Data 1A.pdf]

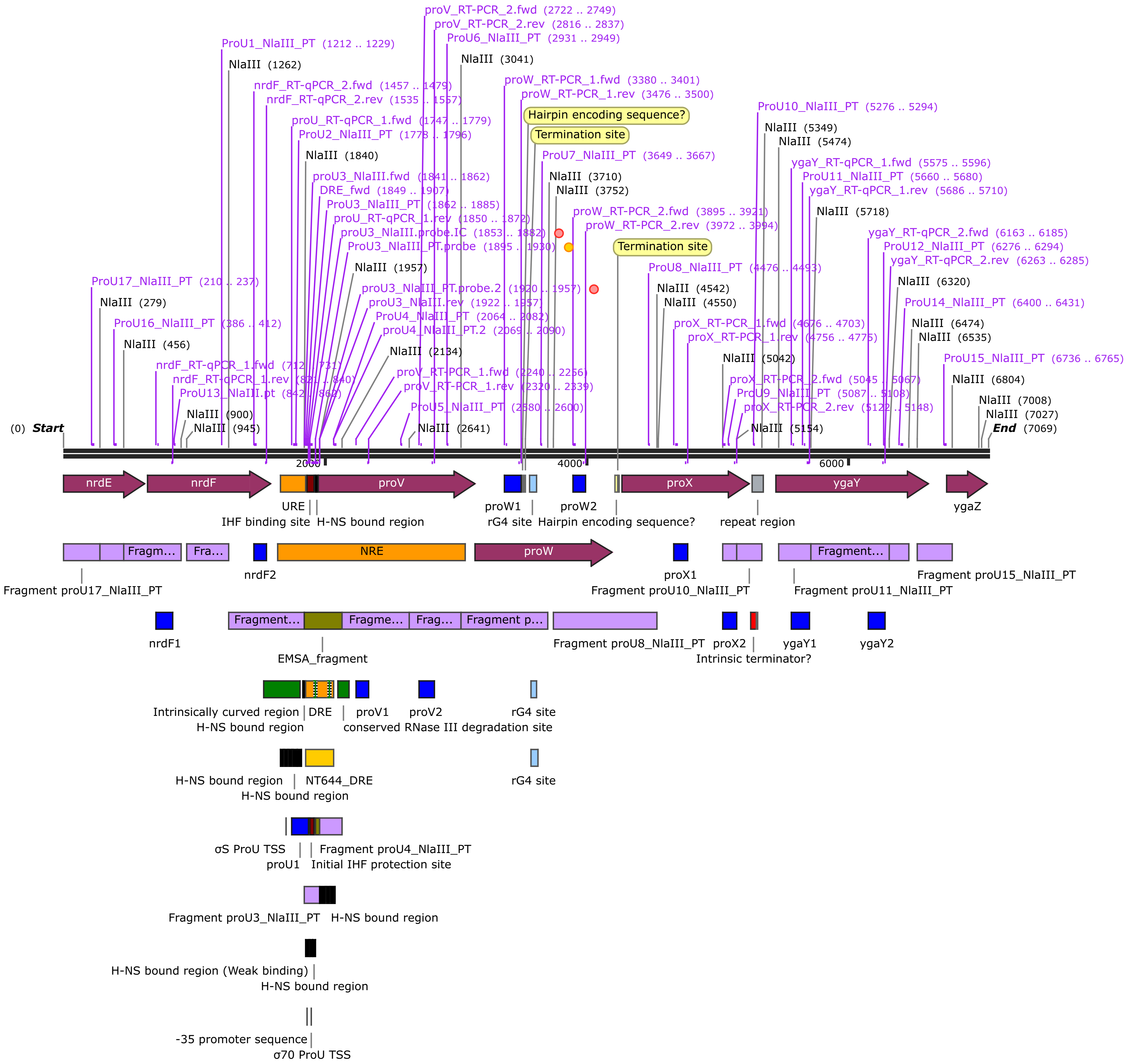
proV_RT-PCR_2.fwd (2722 .. 2749)
proV_RT-PCR_2.rev (2816 .. 2837)
ProU6_NlaIII_PT (2931 .. 2949)
ProU1_NlaIII_PT (1212 .. 1229)
NlaIII (1262)
NlaIII (3041)
proW_RT-PCR_1.fwd (3380 .. 3401)
proW_RT-PCR_1.rev (3476 .. 3500)
nrdF_RT-qPCR_2.fwd (1457 .. 1479)
nrdF_RT-qPCR_2.rev (1535 .. 1557)
ProU10_NlaIII_PT (5276 .. 5294)
Hairpin encoding sequence?
proU_RT-qPCR_1.fwd (1747 .. 1779)
ProU2_NlaIII_PT (1778 .. 1796)
Termination site
NlaIII (5349)
NlaIII (5474)
NlaIII (1840)
ProU7_NlaIII_PT (3649 .. 3667)
ygaY_RT-qPCR_1.fwd (5575 .. 5596)
proU3_NlaIII.fwd (1841 .. 1862)
NlaIII (3710)
ProU11_NlaIII_PT (5660 .. 5680)
DRE_fwd (1849 .. 1907)
NlaIII (3752)
ygaY_RT-qPCR_1.rev (5686 .. 5710)
ProU3_NlaIII_PT (1862 .. 1885)
proW_RT-PCR_2.fwd (3895 .. 3921)
NlaIII (5718)
proU_RT-qPCR_1.rev (1850 .. 1872)
proW_RT-PCR_2.rev (3972 .. 3994)
proU3_NlaIII.probe.IC (1853 .. 1882)
ygaY_RT-qPCR_2.fwd (6163 .. 6185)
ProU3_NlaIII_PT.probe (1895 .. 1930)
Termination site
ProU12_NlaIII_PT (6276 .. 6294)
ygaY_RT-qPCR_2.rev (6263 .. 6285)
NlaIII (1957)
ProU8_NlaIII_PT (4476 .. 4493)
ProU17_NlaIII_PT (210 .. 237)
proU3_NlaIII_PT.probe.2 (1920 .. 1957)
NlaIII (4542)
NlaIII (6320)
NlaIII (279)
proU3_NlaIII.rev (1922 .. 1957)
NlaIII (4550)
ProU14_NlaIII_PT (6400 .. 6431)
ProU4_NlaIII_PT (2064 .. 2082)
ProU16_NlaIII_PT (386 .. 412)
proU4_NlaIII_PT.2 (2069 .. 2090)
proX_RT-PCR_1.fwd (4676 .. 4703)
NlaIII (6474)
proX_RT-PCR_1.rev (4756 .. 4775)
NlaIII (6535)
NlaIII (456)
NlaIII (2134)
NlaIII (5042)
ProU15_NlaIII_PT (6736 .. 6765)
nrdF_RT-qPCR_1.fwd (712 .. 731)
proV_RT-PCR_1.fwd (2240 .. 2256)
proX_RT-PCR_2.fwd (5045 .. 5067)
NlaIII (6804)
nrdF_RT-qPCR_1.rev (821 .. 840)
proV_RT-PCR_1.rev (2320 .. 2339)
ProU9_NlaIII_PT (5087 .. 5108)
ProU13_NlaIII.pt (842 .. 860)
NlaIII (7008)
ProU5_NlaIII_PT (2580 .. 2600)
proX_RT-PCR_2.rev (5122 .. 5148)
NlaIII (900)
NlaIII (7027)
(0) Start
NlaIII (945)
NlaIII (2641)
NlaIII (5154)
End (7069)
2000
4000
6000
nrdE
nrdF
proV
proX
ygaY
ygaZ
URE
IHF binding site
H-NS bound region
proW1
rG4 site
proW2
Hairpin encoding sequence?
repeat region
Fragm…
Fra…
NRE
proW
Fragment…
Fragment proU17_NlaIII_PT
nrdF2
proX1
Fragment proU10_NlaIII_PT
Fragment proU11_NlaIII_PT
Fragment proU15_NlaIII_PT
Fragment…
Fragme…
Frag…
Fragment p…
nrdF1
EMSA_fragment
Fragment proU8_NlaIII_PT
proX2
Intrinsic terminator?
ygaY1
ygaY2
Intrinsically curved region
DRE
proV1
proV2
rG4 site
H-NS bound region
conserved RNase III degradation site
H-NS bound region
NT644_DRE
rG4 site
H-NS bound region
σS ProU TSS
proU1
Fragment proU4_NlaIII_PT
Initial IHF protection site
Fragment proU3_NlaIII_PT
H-NS bound region
H-NS bound region (Weak binding)
H-NS bound region
-35 promoter sequence
σ70 ProU TSS

SI_1A
7069 bp

Start (0)

5′ CACTGCGTACCTCGATGTTGCTGGCACGCGAACGCGGTGAAACCTTCGCCGGGTTCAAACAGTCACGCTATGCCAGTGGTGAATA 85
3′ GTGACGCATGGAGCTACAACGACCGTGCGCTTGCGCCACTTTGGAAGCGGCCCAAGTTTGTCAGTGCGATACGGTCACCACTTAT

510 515 520 525 530
L R T S M L L A R E R G E T F A G F K Q S R Y A S G E Y
nrdE
Fragment proU17_NlaIII_PT

TTTTAGCCAATATCTGCAAGGGAACTGGCAGCCGAAAACGGCGAAAGTTGGCGAACTGTTTACCCGTAGCGGTATTACGTTACCT 170
AAAATCGGTTATAGACGTTCCCTTGACCGTCGGCTTTTGCCGCTTTCAACCGCTTGACAAATGGGCATCGCCATAATGCAATGGA

535 540 545 550 555 560
F S Q Y L Q G N W Q P K T A K V G E L F T R S G I T L P
nrdE
Fragment proU17_NlaIII_PT

ProU17_NlaIII_PT
CGCTACGGCATATACAACCAGAATCTTC

ACCCGTGAGATGTGGGCGCAGCTGCGCGACGACGTGATGCGCTACGGCATATACAACCAGAATCTTCAGGCGGTGCCGCCAACCG 255
TGGGCACTCTACACCCGCGTCGACGCGCTGCTGCACTACGCGATGCCGTATATGTTGGTCTTAGAAGTCCGCCACGGCGGTTGGC

565 570 575 580 585
T R E M W A Q L R D D V M R Y G I Y N Q N L Q A V P P T
nrdE
Fragment proU17_NlaIII_PT

NlaIII
GTTCTATCTCTTATATCAACCATGCTACGTCGAGTATTCATCCGATTGTGGCGAAAGTAGAGATACGCAAAGAGGGCAAAACAGG 340
CAAGATAGAGAATATAGTTGGTACGATGCAGCTCATAAGTAGGCTAACACCGCTTTCATCTCTATGCGTTTCTCCCGTTTTGTCC

590 595 600 605 610 615
G S I S Y I N H A T S S I H P I V A K V E I R K E G K T G
nrdE
Fragment proU17_NlaIII_PT | Fragment proU16_NlaIII_PT

ProU16_NlaIII_PT
GCTGTATCAGGACGCTTACGAAATTGG

ACGCGTTTACTACCCTGCCCCGTTTATGACTAACGAGAATCTGGCGCTGTATCAGGACGCTTACGAAATTGGCGCAGAAAAGATC 425
TGCGCAAATGATGGGACGGGGCAAATACTGATTGCTCTTAGACCGCGACATAGTCCTGCGAATGCTTTAACCGCGTCTTTTCTAG

620 625 630 635 640 645
R V Y Y P A P F M T N E N L A L Y Q D A Y E I G A E K I
nrdE
Fragment proU16_NlaIII_PT

NlaIII
ATCGACACCTACGCGGAAGCGACTCGCCATGTCGATCAGGGGCTGTCGCTGACGCTTTTTTTCCCCGATACCGCCACCACTCGCG 510
TAGCTGTGGATGCGCCTTCGCTGAGCGGTACAGCTAGTCCCCGACAGCGACTGCGAAAAAAAGGGGCTATGGCGGTGGTGAGCGC

650 655 660 665 670
I D T Y A E A T R H V D Q G L S L T L F F P D T A T T R
nrdE
Fragment proU16_NlaIII_PT | Fragment proU13_NlaIII_PT

ATATCAACAAAGCGCAGATTTACGCCTGGCGCAAGGGTATCAAAACGCTCTATTACATCCGCCTGCGTCAGATGGCGCTGGAAGG 595
TATAGTTGTTTCGCGTCTAAATGCGGACCGCGTTCCCATAGTTTTGCGAGATAATGTAGGCGGACGCAGTCTACCGCGACCTTCC

675 680 685 690 695 700
D I N K A Q I Y A W R K G I K T L Y Y I R L R Q M A L E G
nrdE
Fragment proU13_NlaIII_PT

CACTGAAATTGAAGGCTGCGTCTCCTGTGCACTTTAAGGAATATCTATGAAACTCTCACGTATCAGCGCCATCAACTGGAACAAG 680
GTGACTTTAACTTCCGACGCAGAGGACACGTGAAATTCCTTATAGATACTTTGAGAGTGCATAGTCGCGGTAGTTGACCTTGTTC

705 710 715 | 1 5 10
T E I E G C V S C A L * | M K L S R I S A I N W N K
nrdE | nrdF
Fragment proU13_NlaIII_PT

nrdF_RT-qPCR_1.fwd
ATCGCCTGACCAGCAATTTC

```
ATATCTGACGATAAAGATCTGGAGGTGTGGAATCGCCTGACCAGCAATTTCTGGCTACCAGAAAAGGTGCCGCTGTCGAACGATA   765
TATAGACTGCTATTTCTAGACCTCCACACCTTAGCGGACTGGTCGTTAAAGACCGATGGTCTTTTCCACGGCGACAGCTTGCTAT
    15        20        25        30        35        40
I S D D K D L E V W N R L T S N F W L P E K V P L S N D
```

nrdF

Fragment proU13_NlaIII_PT

nrdF1

ProU13_NlaIII.pt
cacgctgca

```
TTCCTGCCTGGCAGACATTAACTGTCGTAGAACAACAACTGACGATGCGCGTTTTTACTGGCCTGACGCTGCTCGACACGCTGCA   850
AAGGACGGACCGTCTGTAATTGACAGCATCTTGTTGTTGACTGCTACGCGCAAAAATGACCGGACTGCGACGAGCTGTGCGACGT
    45        50        55        60        65        70
I P A W Q T L T V V E Q Q L T M R V F T G L T L L D T L Q
```

nrdF

Fragment proU13_NlaIII_PT

nrdF1

ATGACCGGACTGCGACGAGC
nrdF_RT-qPCR_1.rev

ProU13_NlaIII.pt
aaatgttatcgg

NlaIII

```
AAATGTTATCGGCGCGCCTTCTCTGATGCCCGATGCACTCACGCCTCATGAAGAAGCGGTATTATCGAATATCAGCTTTATGGAA   935
TTTACAATAGCCGCGCGGAAGAGACTACGGGCTACGTGAGTGCGGAGTACTTCTTCGCCATAATAGCTTATAGTCGAAATACCTT
    75        80        85        90        95
N V I G A P S L M P D A L T P H E E A V L S N I S F M E
```

nrdF

Fragment proU13_NlaIII_PT

NlaIII

```
GCGGTTCATGCCCGCTCTTACAGTTCGATTTTCTCGACGCTATGCCAGACCAAAGATGTCGATGCCGCCTACGCCTGGAGTGAAG   1020
CGCCAAGTACGGGCGAGAATGTCAAGCTAAAAGAGCTGCGATACGGTCTGGTTTCTACAGCTACGGCGGATGCGGACCTCACTTC
    100       105       110       115       120       125
A V H A R S Y S S I F S T L C Q T K D V D A A Y A W S E
```

nrdF

Fragment proU1_NlaIII_PT

```
AAAACGCACCGTTGCAGCGAAAAGCTCAGATTATTCAGCAACATTATCGCGGTGATGATCCGCTGAAAAAGAAAATCGCCAGTGT   1105
TTTTGCGTGGCAACGTCGCTTTTCGAGTCTAATAAGTCGTTGTAATAGCGCCACTACTAGGCGACTTTTTCTTTTAGCGGTCACA
    130       135       140       145       150       155
E N A P L Q R K A Q I I Q Q H Y R G D D P L K K I A S V
```

nrdF

Fragment proU1_NlaIII_PT

```
GTTTCTTGAATCTTTTTTGTTCTATTCCGGTTTCTGGCTGCCGATGTATTTTTCCAGCCGCGGAAAGCTGACCAATACCGCGGAC   1190
CAAAGAACTTAGAAAAAACAAGATAAGGCCAAAGACCGACGGCTACATAAAAAGGTCGGCGCCTTTCGACTGGTTATGGCGCCTG
    160       165       170       175       180
F L E S F L F Y S G F W L P M Y F S S R G K L T N T A D
```

nrdF

Fragment proU1_NlaIII_PT

ProU1_NlaIII_PT
gatgaagcagtccacggt

NlaIII

```
CTGATCCGTCTGATTATCCGCGATGAAGCAGTCCACGGTTACTACATAGGCTATAAATATCAGAAAAACATGGAAAAGATATCTC   1275
GACTAGGCAGACTAATAGGCGCTACTTCGTCAGGTGCCAATGATGTATCCGATATTTATAGTCTTTTTGTACCTTTTCTATAGAG
    185       190       195       200       205       210
L I R L I I R D E A V H G Y Y I G Y K Y Q K N M E K I S
```

nrdF

Fragment proU1_NlaIII_PT

Fragment proU2_NlaIII_PT

TGGGACAACGTGAAGAGTTGAAGAGTTTCGCCTTCGATTTGTTGCTGGAACTCTACGACAACGAGTTGCAATACACCGATGAGCT 1360
ACCCTGTTGCACTTCTCAACTTCTCAAAGCGGAAGCTAAACAACGACCTTGAGATGCTGTTGCTCAACGTTATGTGGCTACTCGA

215 220 225 230 235 240
L G Q R E E L K S F A F D L L L E L Y D N E L Q Y T D E L
nrdF
Fragment proU2_NlaIII_PT

GTACGCCGAAACCCCGTGGGCTGACGATGTGAAAGCGTTTCTCTGTTACAACGCCAATAAGGCTTTGATGAATCTGGGCTACGAA 1445
CATGCGGCTTTGGGGCACCCGACTGCTACACTTTCGCAAAGAGACAATGTTGCGGTTATTCCGAAACTACTTAGACCCGATGCTT

245 250 255 260 265
Y A E T P W A D D V K A F L C Y N A N K A L M N L G Y E
nrdF
Fragment proU2_NlaIII_PT

nrdF_RT-qPCR_2.fwd
CGCAGAAATGGCGGAAGTGAATC

CCGTTATTTCCCGCAGAAATGGCGGAAGTGAATCCGGCAATCCTCGCCGCGCTTTCGCCGAATGCCGATGAAAATCACGATTTCT 1530
GGCAATAAAGGGCGTCTTTACCGCCTTCACTTAGGCCGTTAGGAGCGGCGCGAAAGCGGCTTACGGCTACTTTTAGTGCTAAAGA

270 275 280 285 290 295
P L F P A E M A E V N P A I L A A L S P N A D E N H D F
nrdF
Fragment proU2_NlaIII_PT
nrdF2

TTTCCGGTTCAGGCTCCTCTTATGTGATGGGGAAAGCGGTTGAAACAGAAGATGAAGACTGGAATTTCTGAGGGTGTTATTTTCA 1615
AAAGGCCAAGTCCGAGGAGAATACACTACCCCTTTCGCCAACTTTGTCTTCTACTTCTGACCTTAAAGACTCCCACAATAAAAGT

300 305 310 315 320
F S G S G S S Y V M G K A V E T E D E D W N F *
nrdF
Fragment proU2_NlaIII_PT
Intrinsically curved region
nrdF2
GCCAAGTCCGAGGAGAATACACT
nrdF_RT-qPCR_2.rev

AAAATATCACTACCCGCAGCAGGGAAATAATTCCCGCCAAATAGCTTTTTATCACGCAAATAATTTGTGGTGATCTACACTGATA 1700
TTTTATAGTGATGGGCGTCGTCCCTTTATTAAGGGCGGTTTATCGAAAAATAGTGCGTTTATTAAACACCACTAGATGTGACTAT

NRE
Fragment proU2_NlaIII_PT
Intrinsically curved region
URE
H-NS bound region
H-NS bound region

ProU2_NlaIII_PT
cgatttgc

proU_RT-qPCR_1.fwd
CGCTATCTTTGACAAAAAAATATCAACTTTCTCG

CTCTGTTGCATTATTCGCCTGAAACCACAATATTCAGGCGTTTTTTCGCTATCTTTGACAAAAAAATATCAACTTTCTCGATTTGC 1785
GAGACAACGTAATAAGCGGACTTTGGTGTTATAAGTCCGCAAAAAAGCGATAGAAACTGTTTTTTTATAGTTGAAAGAGCTAAACG

NRE
Fragment proU2_NlaIII_PT
Intrinsically curved region
URE
H-NS bound region
proU1
σS ProU TSS
H-NS bound region
H-NS bound region

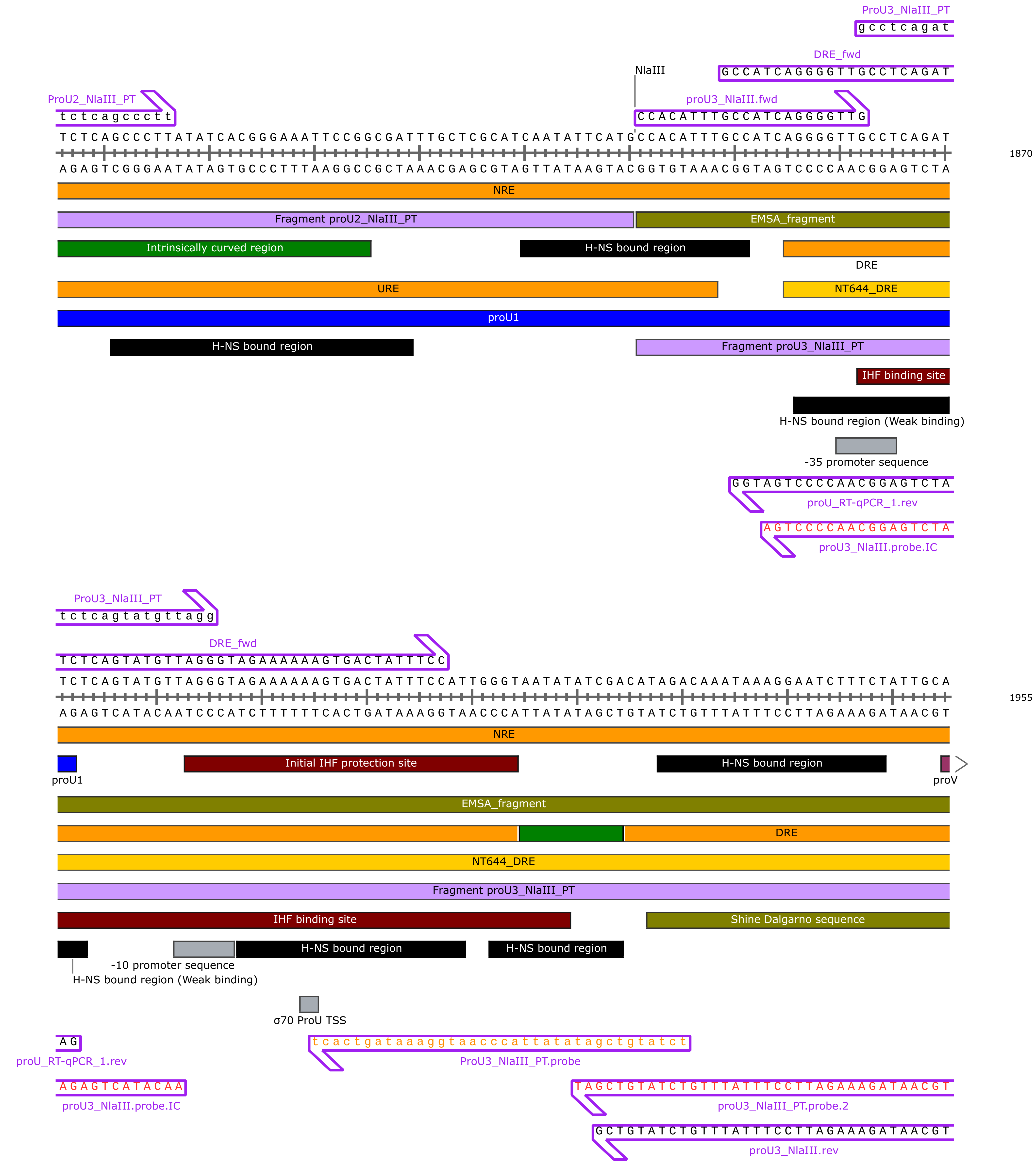

ProU3_NlaIII_PT
gcctcagat
DRE_fwd
GCCATCAGGGGTTGCCTCAGAT
NlaIII
ProU2_NlaIII_PT
tctcagccctt
proU3_NlaIII.fwd
CCACATTTGCCATCAGGGGTTG
TCTCAGCCCTTATATCACGGGAAATTCCGGCGATTTGCTCGCATCAATATTCATGCCACATTTGCCATCAGGGGTTGCCTCAGAT
1870
AGAGTCGGGAATATAGTGCCCTTTAAGGCCGCTAAACGAGCGTAGTTATAAGTACGGTGTAAACGGTAGTCCCCAACGGAGTCTA
NRE
Fragment proU2_NlaIII_PT
EMSA_fragment
Intrinsically curved region
H-NS bound region
DRE
URE
NT644_DRE
proU1
H-NS bound region
Fragment proU3_NlaIII_PT
IHF binding site
H-NS bound region (Weak binding)
-35 promoter sequence
GGTAGTCCCCAACGGAGTCTA
proU_RT-qPCR_1.rev
AGTCCCCAACGGAGTCTA
proU3_NlaIII.probe.IC
ProU3_NlaIII_PT
tctcagtatgttagg
DRE_fwd
TCTCAGTATGTTAGGGTAGAAAAAAGTGACTATTTCC
TCTCAGTATGTTAGGGTAGAAAAAAGTGACTATTTCCATTGGGTAATATATCGACATAGACAAATAAAGGAATCTTTCTATTGCA
1955
AGAGTCATACAATCCCATCTTTTTTCACTGATAAAGGTAACCCATTATATAGCTGTATCTGTTTATTTCCTTAGAAAGATAACGT
NRE
proU1
Initial IHF protection site
H-NS bound region
proV
EMSA_fragment
DRE
NT644_DRE
Fragment proU3_NlaIII_PT
IHF binding site
Shine Dalgarno sequence
H-NS bound region
H-NS bound region
-10 promoter sequence
H-NS bound region (Weak binding)
σ70 ProU TSS
AG
proU_RT-qPCR_1.rev
tcactgataaaggtaacccattatatagctgtatct
ProU3_NlaIII_PT.probe
AGAGTCATACAA
proU3_NlaIII.probe.IC
TAGCTGTATCTGTTTATTTCCTTAGAAAGATAACGT
proU3_NlaIII_PT.probe.2
GCTGTATCTGTTTATTTCCTTAGAAAGATAACGT
proU3_NlaIII.rev

NlaIII

```
TGGCAATTAAATTAGAAATTAAAAATCTTTATAAAATATTTGGCGAGCATCCACAGCGAGCGTTCAAATATATCGAACAAGGACT  2040
ACCGTTAATTTAATCTTTAATTTTTAGAAATATTTTATAAACCGCTCGTAGGTGTCGCTCGCAAGTTTATATAGCTTGTTCCTGA
```

NRE

1 5 10 15 20 25
M A I K L E I K N L Y K I F G E H P Q R A F K Y I E Q G L

proV

EMSA_fragment

DRE

NT644_DRE

Fragment proU4_NlaIII_PT

Fragment proU3_NlaIII_PT

H-NS bound region (Strong binding)

H-NS bound region

Shine Dalgarno sequence

AC
proU3_NlaIII_PT.probe.2

AC
proU3_NlaIII.rev

proU4_NlaIII_PT.2
GGGCTATCGCTTGGCGTAAAAG

ProU4_NlaIII_PT
aaactgggctatcgcttgg

```
TTCAAAAGAACAAATTCTGGAAAAAAACTGGGCTATCGCTTGGCGTAAAAGACGCCAGTCTGGCCATTGAAGAAGGCGAGATATTT  2125
AAGTTTTCTTGTTTAAGACCTTTTTTTGACCCGATAGCGAACCGCATTTTCTGCGGTCAGACCGGTAACTTCTTCCGCTCTATAAA
```

NRE

30 35 40 45 50 55
S K E Q I L E K T G L S L G V K D A S L A I E E G E I F

proV

EMSA_fragment

DRE

conserved RNase III degradation site

NT644_DRE

Fragment proU4_NlaIII_PT

H-NS bound region

NlaIII

```
GTCATCATGGGATTATCCGGCTCGGGTAAATCCACAATGGTACGCCTTCTCAATCGCCTGATTGAACCCACCCGCGGGCAAGTGC  2210
CAGTAGTACCCTAATAGGCCGAGCCCATTTAGGTGTTACCATGCGGAAGAGTTAGCGGACTAACTTGGGTGGGCGCCCGTTCACG
```

NRE

60 65 70 75 80 85
V I M G L S G S G K S T M V R L L N R L I E P T R G Q V

proV

Fragment proU5_NlaIII_PT

EMSA_fragment

Fragment proU4_NlaIII_PT

conserved RNase III degradation site

proV_RT-PCR_1.fwd
TCCGACGCCGAACTCCG

```
TGATTGATGGTGTGGATATTGCCAAAATATCCGACGCCGAACTCCGTGAGGTGCGCAGAAAAAGATTGCGATGGTCTTCCAGTC  2295
ACTAACTACCACACCTATAACGGTTTTATAGGCTGCGGCTTGAGGCACTCCACGCGTCTTTTTCTAACGCTACCAGAAGGTCAG
```

NRE

90 95 100 105 110
L I D G V D I A K I S D A E L R E V R R K K I A M V F Q S

proV

Fragment proU5_NlaIII_PT

proV1

CTTTGCCTTAATGCCGCATATGACCGTGCTGGACAATACTGCGTTCGGTATGGAATTGGCCGGAATTAATGCCGAAGAACGCCGG 2380
GAAACGGAATTACGGCGTATACTGGCACGACCTGTTATGACGCAAGCCATACCTTAACCGGCCTTAATTACGGCTTCTTGCGGCC
NRE
115 120 125 130 135 140
F A L M P H M T V L D N T A F G M E L A G I N A E E R R
proV
Fragment proU5_NlaIII_PT
proV1
GCACGACCTGTTATGACGCA
proV_RT-PCR_1.rev

GAAAAAGCCCTTGATGCACTGCGTCAGGTCGGGCTGGAAAATTATGCCCACAGCTACCCGGATGAACTCTCTGGCGGGATGCGTC 2465
CTTTTTCGGGAACTACGTGACGCAGTCCAGCCCGACCTTTTAATACGGGTGTCGATGGGCCTACTTGAGAGACCGCCCTACGCAG
NRE
145 150 155 160 165 170
E K A L D A L R Q V G L E N Y A H S Y P D E L S G G M R
proV
Fragment proU5_NlaIII_PT

AACGTGTGGGATTAGCCCGCGCGTTAGCGATTAATCCGGATATATTATTAATGGACGAAGCCTTCTCGGCGCTCGATCCATTAAT 2550
TTGCACACCCTAATCGGGCGCGCAATCGCTAATTAGGCCTATATAATAATTACCTGCTTCGGAAGAGCCGCGAGCTAGGTAATTA
NRE
175 180 185 190 195
Q R V G L A R A L A I N P D I L L M D E A F S A L D P L I
proV
Fragment proU5_NlaIII_PT

ProU5_NlaIII_PT
aattacaggcgaaacatcagc
TCGCACCGAGATGCAGGATGAGCTGGTAAAATTACAGGCGAAACATCAGCGCACCATTGTCTTTATTTCCCACGATCTTGATGAA 2635
AGCGTGGCTCTACGTCCTACTCGACCATTTTAATGTCCGCTTTGTAGTCGCGTGGTAACAGAAATAAAGGGTGCTAGAACTACTT
NRE
200 205 210 215 220 225
R T E M Q D E L V K L Q A K H Q R T I V F I S H D L D E
proV
Fragment proU5_NlaIII_PT

NlaIII
GCCATGCGTATTGGCGACCGAATTGCCATTATGCAAAATGGTGAAGTGGTACAGGTCGGCACACCGGATGAAATTCTCAATAATC 2720
CGGTACGCATAACCGCTGGCTTAACGGTAATACGTTTTACCACTTCACCATGTCCAGCCGTGTGGCCTACTTTAAGAGTTATTAG
NRE
230 235 240 245 250 255
A M R I G D R I A I M Q N G E V V Q V G T P D E I L N N
proV
Fragme... Fragment proU6_NlaIII_PT

proV_RT-PCR_2.fwd
GGCGAATGATTATGTCCGTACCTTCTTC
CGGCGAATGATTATGTCCGTACCTTCTTCCGTGGCGTTGATATTAGTCAGGTATTCAGTGCGAAAGATATTGCCCGCCGGACACC 2805
GCCGCTTACTAATACAGGCATGGAAGAAGGCACCGCAACTATAATCAGTCCATAAGTCACGCTTTCTATAACGGGCGGCCTGTGG
NRE
260 265 270 275 280
P A N D Y V R T F F R G V D I S Q V F S A K D I A R R T P
proV
Fragment proU6_NlaIII_PT
proV2

```
GAATGGCTTAATTCGTAAAACCCCTGGCTTCGGCCCACGTTCGGCACTGAAATTATTGCAGGATGAAGATCGCGAATATGGCTAC    2890
CTTACCGAATTAAGCATTTTGGGGACCGAAGCCGGGTGCAAGCCGTGACTTTAATAACGTCCTACTTCTAGCGCTTATACCGATG
NRE
 285       290       295       300       305       310
 N G L I R K T P G F G P R S A L K L L Q D E D R E Y G Y
proV
Fragment proU6_NlaIII_PT
proV2
TAAGCATTTTGGGGACCGAAGC
proV_RT-PCR_2.rev

ProU6_NlaIII_PT
tcgattcgcttaaaaccgc
GTTATCGAACGCGGTAATAAGTTTGTCGGCGCAGTCTCCATCGATTCGCTTAAAACCGCGTTAACGCAGCAGCAAGGTCTTGATG    2975
CAATAGCTTGCGCCATTATTCAAACAGCCGCGTCAGAGGTAGCTAAGCGAATTTTGGCGCAATTGCGTCGTCGTTCCAGAACTAC
NRE
 315       320       325       330       335       340
 V I E R G N K F V G A V S I D S L K T A L T Q Q Q G L D
proV
Fragment proU6_NlaIII_PT

                                                                     NlaIII
CGGCGCTGATTGATGCGCCGTTAGCAGTCGATGCACAAACGCCTCTTAGCGAGTTGCTCTCTCATGTCGGACAGGCACCCTGTGC    3060
GCCGCGACTAACTACGCGGCAATCGTCAGCTACGTGTTTGCGGAGAATCGCTCAACGAGAGAGTACAGCCTGTCCGTGGGACACG
NRE
 345       350       355       360       365
 A A L I D A P L A V D A Q T P L S E L L S H V G Q A P C A
proV
Fragment proU6_NlaIII_PT | Fragment proU7_NlaIII_PT

GGTGCCCGTGGTCGACGAGGACCAACAGTATGTCGGCATCATTTCGAAAGGAATGCTGCTGCGCGCTTTAGATCGTGAGGGGGTA    3145
CCACGGGCACCAGCTGCTCCTGGTTGTCATACAGCCGTAGTAAAGCTTTCCTTACGACGACGCGCGAAATCTAGCACTCCCCCAT
NRE
 370       375       380       385       390       395
 V P V V D E D Q Q Y V G I I S K G M L L R A L D R E G V
proV
Fragment proU7_NlaIII_PT

AATAATGGCTGATCAAAATAATCCGTGGGATACCACGCCAGCGGCGGACAGTGCCGCGCAATCCGCAGACGCCTGGGGTACACCG    3230
TTATTACCGACTAGTTTTATTAGGCACCCTATGGTGCGGTCGCCGCCTGTCACGGCGCGTTAGGCGTCTGCGGACCCCATGTGGC
 400
 N N G *
proV
 1       5       10       15       20       25
 M A D Q N N P W D T T P A A D S A A Q S A D A W G T P
proW
Fragment proU7_NlaIII_PT

ACGACTGCACCGACTGACGGCGGTGGTGCTGACTGGCTGACCAGTACGCCTGCGCCAAACGTCGAGCATTTTAATATTCTCGATC    3315
TGCTGACGTGGCTGACTGCCGCCACCACGACTGACCGACTGGTCATGCGGACGCGGTTTGCAGCTCGTAAAATTATAAGAGCTAG
 30       35       40       45       50       55
 T T A P T D G G G A D W L T S T P A P N V E H F N I L D
proW
Fragment proU7_NlaIII_PT

                                                                  proW_RT-PCR_1.fwd
                                                                  CCATTTCCGTCCCGTCTTCCA
CGTTCCATAAAACGCTGATCCCGCTCGACAGTTGGGTCACTGAAGGGATCGACTGGGTCGTTACCCATTTCCGTCCCGTCTTCCA    3400
GCAAGGTATTTTGCGACTAGGGCGAGCTGTCAACCCAGTGACTTCCCTAGCTGACCCAGCAATGGGTAAAGGCAGGGCAGAAGGT
 60       65       70       75       80
 P F H K T L I P L D S W V T E G I D W V V T H F R P V F Q
proW
Fragment proU7_NlaIII_PT
proW1
```

proW_RT-PCR_1.fwd
G

```
GGGCGTGCGCGTTCCGGTTGATTATATCCTCAACGGTTTCCAGCAATTGCTGCTGGGTATGCCCGCACCGGTGGCGATTATCGTT 3485
CCCGCACGCGCAAGGCCAACTAATATAGGAGTTGCCAAAGGTCGTTAACGACGACCCATACGGGCGTGGCCACCGCTAATAGCAA
```

85 90 95 100 105 110
G V R V P V D Y I L N G F Q Q L L L G M P A P V A I I V

proW

Fragment proU7_NlaIII_PT

proW1

CTAATAGCAA
proW_RT-PCR_1.rev

```
TTCGCTCTCATCGCCTGGCAGATTTCCGGGGTCGGAATGGGTGTGGCGACGCTGGTTTCGCTGATTGCCATCGGCGCAATCGGTG 3570
AAGCGAGAGTAGCGGACCGTCTAAAGGCCCCAGCCTTACCCACACCGCTGCGACCAAAGCGACTAACGGTAGCCGCGTTAGCCAC
```

115 120 125 130 135 140
F A L I A W Q I S G V G M G V A T L V S L I A I G A I G

proW

Fragment proU7_NlaIII_PT

proW1

Hairpin encoding sequence?

Termination site

AAGCGAGAGTAGCGG
proW_RT-PCR_1.rev

ProU7_NlaIII_PT
GGATATG

```
CCTGGTCGCAGGCAATGGTGACTCTGGCGCTGGTGTTAACCGCCCTGCTGTTCTGTATCGTCATCGGTTTGCCGTTGGGGATATG 3655
GGACCAGCGTCCGTTACCACTGAGACCGCGACCACAATTGGCGGGACGACAAGACATAGCAGTAGCCAAACGGCAACCCCTATAC
```

145 150 155 160 165
A W S Q A M V T L A L V L T A L L F C I V I G L P L G I W

proW

Fragment proU7_NlaIII_PT

rG4 site

rG4 site

rG4 site

ProU7_NlaIII_PT
GCTGGCGAGAAG

NlaIII

```
GCTGGCGAGAAGTCCGCGAGCGGCGAAAATTATTCGTCCACTGCTTGATGCCATGCAGACCACGCCAGCGTTTGTTTATCTGGTG 3740
CGACCGCTCTTCAGGCGCTCGCCGCTTTTAATAAGCAGGTGACGAACTACGGTACGTCTGGTGCGGTCGCAAACAAATAGACCAC
```

170 175 180 185 190 195
L A R S P R A A K I I R P L L D A M Q T T P A F V Y L V

proW

Fragment proU7_NlaIII_PT

NlaIII

```
CCAATCGTCATGCTATTTGGTATCGGTAACGTGCCGGGCGTGGTGGTGACGATCATCTTTGCTCTGCCGCCGATTATCCGTCTGA 3825
GGTTAGCAGTACGATAAACCATAGCCATTGCACGGCCCGCACCACCACTGCTAGTAGAAACGAGACGGCGGCTAATAGGCAGACT
```

200 205 210 215 220 225
P I V M L F G I G N V P G V V V T I I F A L P P I I R L

proW

Fragment proU8_NlaIII_PT

proW_RT-PCR_2.fwd
GCCAGATGCTGTTCAA

```
CCATTCTGGGGATTAACCAGGTTCCGGCGGATCTGATTGAAGCCTCGCGCTCATTCGGTGCCAGCCCGCGCCAGATGCTGTTCAA 3910
GGTAAGACCCCTAATTGGTCCAAGGCCGCCTAGACTAACTTCGGAGCGCGAGTAAGCCACGGTCGGGCGCGGTCTACGACAAGTT
```

230 235 240 245 250
T I L G I N Q V P A D L I E A S R S F G A S P R Q M L F K

proW

Fragment proU8_NlaIII_PT

proW2

proW_RT-PCR_2.fwd
AGTTCAGTTAC
AGTTCAGTTACCGCTGGCGATGCCGACCATTATGGCGGGCGTTAACCAGACGCTGATGCTGGCCCTTTCTATGGTGGTCATCGCC 3995
TCAAGTCAATGGCGACCGCTACGGCTGGTAATACCGCCCGCAATTGGTCTGCGACTACGACCGGGAAAGATACCACCAGTAGCGG
255 260 265 270 275 280
V Q L P L A M P T I M A G V N Q T L M L A L S M V V I A
proW
Fragment proU8_NlaIII_PT
proW2
CGGGAAAGATACCACCAGTAGCG
proW_RT-PCR_2.rev

TCGATGATTGCCGTCGGCGGGTTGGGTCAGATGGTACTTCGCGGTATCGGTCGTCTGGATATGGGGCTTGCCACCGTTGGCGGCG 4080
AGCTACTAACGGCAGCCGCCCAACCCAGTCTACCATGAAGCGCCATAGCCAGCAGACCTATACCCCGAACGGTGGCAACCGCCGC
285 290 295 300 305 310
S M I A V G G L G Q M V L R G I G R L D M G L A T V G G
proW
Fragment proU8_NlaIII_PT

TCGGGATTGTGATCCTCGCCATTATCCTCGATCGTCTGACGCAGGCCGTTGGGCGCGACTCACGCAGTCGCGGCAACCGTCGCTG 4165
AGCCCTAACACTAGGAGCGGTAATAGGAGCTAGCAGACTGCGTCCGGCAACCCGCGCTGAGTGCGTCAGCGCCGTTGGCAGCGAC
315 320 325 330 335
V G I V I L A I I L D R L T Q A V G R D S R S R G N R R W
proW
Fragment proU8_NlaIII_PT

GTACACCACTGGCCCTGTTGGTCTGCTGACCCGCCCATTCATTAAGTAACTCTGCACTTGCCCGGTGACGCCGGGCATTATCACC 4250
CATGTGGTGACCGGGACAACCAGACGACTGGGCGGGTAAGTAATTCATTGAGACGTGAACGGGCCACTGCGGCCCGTAATAGTGG
340 345 350 355
Y T T G P V G L L T R P F I K *
proW
Hairpin encoding sequence?
Termination site
Fragment proU8_NlaIII_PT

CTGCCAAAAAAAGGAATAACAATGCGACATAGCGTACTTTTTGCGACAGCGTTTGCCACGCTTATCTCTACACAAACTTTTGCTG 4335
GACGGTTTTTTTCCTTATTGTTACGCTGTATCGCATGAAAAACGCTGTCGCAAACGGTGCGAATAGAGATGTGTTTGAAAACGAC
1 5 10 15 20
M R H S V L F A T A F A T L I S T Q T F A
proX
Fragment proU8_NlaIII_PT

CCGATCTGCCGGGCAAAGGCATTACTGTTAATCCAGTTCAGAGCACCATCACTGAAGAAACCTTCCAGACGCTGCTGGTCAGTCG 4420
GGCTAGACGGCCCGTTTCCGTAATGACAATTAGGTCAAGTCTCGTGGTAGTGACTTCTTTGGAAGGTCTGCGACGACCAGTCAGC
25 30 35 40 45 50
A D L P G K G I T V N P V Q S T I T E E T F Q T L L V S R
proX
Fragment proU8_NlaIII_PT

ProU8_NlaIII_PT
gttggctacacctcgctt
TGCGCTGGAGAAATTAGGTTATACCGTCAACAAACCCAGCGAAGTAGATTACAACGTTGGCTACACCTCGCTTGCTTCCGGCGAT 4505
ACGCGACCTCTTTAATCCAATATGGCAGTTGTTTGGGTCGCTTCATCTAATGTTGCAACCGATGTGGAGCGAACGAAGGCCGCTA
55 60 65 70 75
A L E K L G Y T V N K P S E V D Y N V G Y T S L A S G D
proX
Fragment proU8_NlaIII_PT

NlaIII NlaIII
GCAACCTTCACCGCCGTGAACTGGACGCCACTGCATGACAACATGTACGAAGCTGCCGGTGGCGATAAGAAATTTTATCGTGAAG 4590
CGTTGGAAGTGGCGGCACTTGACCTGCGGTGACGTACTGTTGTACATGCTTCGACGGCCACCGCTATTCTTTAAAATAGCACTTC
80 85 90 95 100 105
A T F T A V N W T P L H D N M Y E A A G G D K K F Y R E
proX
Fragment proU8_NlaIII_PT

GGGTATTTGTTAACGGCGCGGCACAGGGTTACCTGATCGATAAGAAAACCGCCGACCAGTACAAAATCACCAACATCGCACAACT 4675
CCCATAAACAATTGCCGCGCCGTGTCCCAATGGACTAGCTATTCTTTTGGCGGCTGGTCATGTTTTAGTGGTTGTAGCGTGTTGA
110 115 120 125 130 135
G V F V N G A A Q G Y L I D K K T A D Q Y K I T N I A Q L
proX

proX_RT-PCR_1.fwd
GAAAGATCCGAAGATCGCCAAACTGTTC
GAAAGATCCGAAGATCGCCAAACTGTTCGATACCAACGGCGACGGAAAAGCGGATTTAACCGGTTGTAACCCTGGCTGGGGCTGC 4760
CTTTCTAGGCTTCTAGCGGTTTGACAAGCTATGGTTGCCGCTGCCTTTTCGCCTAAATTGGCCAACATTGGGACCGACCCCGACG
140 145 150 155 160
K D P K I A K L F D T N G D G K A D L T G C N P G W G C
proX
proX1
CGACG
proX_RT-PCR_1.rev

GAAGGTGCGATCAACCACCAGCTTGCCGCGTATGAACTGACCAACACCGTGACGCATAATCAGGGGAACTACGCAGCGATGATGG 4845
CTTCCACGCTAGTTGGTGGTCGAACGGCGCATACTTGACTGGTTGTGGCACTGCGTATTAGTCCCCTTGATGCGTCGCTACTACC
165 170 175 180 185 190
E G A I N H Q L A A Y E L T N T V T H N Q G N Y A A M M
proX
proX1
CTTCCACGCTAGTTG
proX_RT-PCR_1.rev

CCGACACCATCAGTCGCTACAAAGAGGGCAAACCGGTGTTTTATTACACCTGGACGCCGTACTGGGTGAGTAACGAACTGAAGCC 4930
GGCTGTGGTAGTCAGCGATGTTTCTCCCGTTTGGCCACAAAATAATGTGGACCTGCGGCATGACCCACTCATTGCTTGACTTCGG
195 200 205 210 215 220
A D T I S R Y K E G K P V F Y Y T W T P Y W V S N E L K P
proX

GGGCAAAGATGTCGTCTGGTTGCAGGTGCCGTTCTCCGCACTGCCGGGCGATAAAAACGCCGATACCAAACTGCCGAATGGTGCG 5015
CCCGTTTCTACAGCAGACCAACGTCCACGGCAAGAGGCGTGACGGCCCGCTATTTTTGCGGCTATGGTTTGACGGCTTACCACGC
225 230 235 240 245
G K D V V W L Q V P F S A L P G D K N A D T K L P N G A
proX

NlaIII
proX_RT-PCR_2.fwd
TATCGTTGCCAACAAAGCCTGGG
ProU9_NlaIII_PT
agcgaaactgtttg
AATTATGGCTTCCCGGTCAGCACCATGCATATCGTTGCCAACAAAGCCTGGGCCGAGAAAAACCCGGCAGCAGCGAAACTGTTTG 5100
TTAATACCGAAGGGCCAGTCGTGGTACGTATAGCAACGGTTGTTTCGGACCCGGCTCTTTTTGGGCCGTCGTCGCTTTGACAAAC
250 255 260 265 270 275
N Y G F P V S T M H I V A N K A W A E K N P A A A K L F
proX
Fragment proU9_NlaIII_PT
proX2

ProU9_NlaIII_PT
ccattatg
NlaIII
CCATTATGCAGTTGCCAGTGGCAGATATTAACGCCCAGAACGCCATTATGCATGACGGCAAAGCCTCAGAAGGCGATATTCAGGG 5185
GGTAATACGTCAACGGTCACCGTCTATAATTGCGGGTCTTGCGGTAATACGTACTGCCGTTTCGGAGTCTTCCGCTATAAGTCCC
280 285 290 295 300 305
A I M Q L P V A D I N A Q N A I M H D G K A S E G D I Q G
proX
Fragment proU9_NlaIII_PT
Fragment proU10_NlaIII_PT
proX2
GTCTATAATTGCGGGTCTTGCGGTAAT
proX_RT-PCR_2.rev

ACACGTTGATGGTTGGATCAAAGCCCACCAGCAGCAGTTCGATGGCTGGGTGAATGAGGCGCTGGCAGCGCAGAAGTAATTTTTA 5270
TGTGCAACTACCAACCTAGTTTCGGGTGGTCGTCGTCAAGCTACCGACCCACTTACTCCGCGACCGTCGCGTCTTCATTAAAAAT

310 315 320 325 330
H V D G W I K A H Q Q Q F D G W V N E A L A A Q K *
proX
Intrinsic terminator?
Fragment proU10_NlaIII_PT

ProU10_NlaIII_PT
gcggataaggcgtttttgc
NlaIII
TTCGGGCGGATAAGGCGTTTTTGCCGCATCCGCCGTTCTGTGCACAATGCCTGATGCGACGCTGGCGCGTCTTATCATGCCTACA 5355
AAGCCCGCCTATTCCGCAAAAACGGCGTAGGCGGCAAGACACGTGTTACGGACTACGCTGCGACCGCGCAGAATAGTACGGATGT
Fragment proU10_NlaIII_PT
repeat region
Intrinsic terminator?
Termination site

AACCACATCGCACATTTCAACAATCTATCTTTCATCCCATATTCATCAACATCCGCTATTATTGATTTCCAGCTTAATCATCACC 5440
TTGGTGTAGCGTGTAAAGTTGTTAGATAGAAAGTAGGGTATAAGTAGTTGTAGGCGATAATAACTAAAGGTCGAATTAGTAGTGG

NlaIII
TGATGAACAAAAATAATGACTAAACCTAATCATGAGCTTAGCCCGGCGCTGATCGTGCTGATGTCTATCGCCACCGGTCTGGCGG 5525
ACTACTTGTTTTTATTACTGATTTGGATTAGTACTCGAATCGGGCCGCGACTAGCACGACTACAGATAGCGGTGGCCAGACCGCC
1 5 10 15 20
M T K P N H E L S P A L I V L M S I A T G L A
ygaY
Fragment proU11_NlaIII_PT

ygaY_RT-qPCR_1.fwd
CTTTTCCCTTTCCGCCAGTTCG
TAGCCAGTAACTATTACGCCCAGCCATTGCTCGACACCATCGCGCGTAACTTTTCCCTTTCCGCCAGTTCGGCAGGCTTTATTGT 5610
ATCGGTCATTGATAATGCGGGTCGGTAACGAGCTGTGGTAGCGCGCATTGAAAAGGGAAAGGCGGTCAAGCCGTCCGAAATAACA
25 30 35 40 45 50
V A S N Y Y A Q P L L D T I A R N F S L S A S S A G F I V
ygaY
Fragment proU11_NlaIII_PT
ygaY1

ProU11_NlaIII_PT
CTCGGTGATATGTTTGAACGC
TACCGCCGCGCAGTTGGGCTATGCCGCAGGTCTACTGTTTCTTGTTCCCCTCGGTGATATGTTTGAACGCCGCCGCCTGATTGTC 5695
ATGGCGGCGCGTCAACCCGATACGGCGTCCAGATGACAAAGAACAAGGGGAGCCACTATACAAACTTGCGGCGGCGGACTAACAG
55 60 65 70 75 80
T A A Q L G Y A A G L L F L V P L G D M F E R R R L I V
ygaY
Fragment proU11_NlaIII_PT
ygaY1
GGACTAACAG
ygaY_RT-qPCR_1.rev

NlaIII
TCGATGACCTTACTGGCGGCATGTTGATTACCGCCAGCAGTCAGTCGCTGGCGATGATGATCCTCGGTACGGCATTAACCGGTTT 5780
AGCTACTGGAATGACCGCCGTACAACTAATGGCGGTCGTCAGTCAGCGACCGCTACTACTAGGAGCCATGCCGTAATTGGCCAAA
85 90 95 100 105
S M T L L A A C * L P P A V S R W R * * S S V R H * P V
ygaY
Fragment proU11_NlaIII_PT
Fragment proU12_NlaIII_PT
ygaY1
AGCTACTGGAATGAC
ygaY_RT-qPCR_1.rev

```
ATTCTCAGTCGTGGCACAAATTCTGGTTCCGCTGGCAGCGACGCTGGCTTCACCGGATAAACGCGGCAAAGTGGTTGGCACTATT   5865
TAAGAGTCAGCACCGTGTTTAAGACCAAGGCGACCGTCGCTGCGACCGAAGTGGCCTATTTGCGCCGTTTCACCAACCGTGATAA
  110        115        120        125        130        135
Y S Q S W H K F W F R W Q R R W L H R I N A A K W L A L L
```
ygaY

Fragment proU12_NlaIII_PT

```
ATGAGCGGTCTGCTGTTGGGGATCTTGCTGGCACGGACAGTTGCCGGATTGCTGGCGAATCTCGGCGGCTGGCGCACCGTCTTTT   5950
TACTCGCCAGACGACAACCCCTAGAACGACCGTGCCTGTCAACGGCCTAACGACCGCTTAGAGCCGCCGACCGCGTGGCAGAAAA
    140        145        150        155        160        165
* A V C C W G S C W H G Q L P D C W R I S A A G A P S F
```
ygaY

Fragment proU12_NlaIII_PT

```
GGGTTGCTTCGGTGTTAATGGCACTGATGGCGCTGGCATTATGGCGTGGTCTGCCACAAATGAAATCAGAAACCCACCTCAACTA   6035
CCCAACGAAGCCACAATTACCGTGACTACCGCGACCGTAATACCGCACCAGACGGTGTTTACTTTAGTCTTTGGGTGGAGTTGAT
      170        175        180        185        190
G L L R C * W H * W R W H Y G V V C H K * N Q K P T S T
```
ygaY

Fragment proU12_NlaIII_PT

```
CCCACAGTTGTTGGGTTCCGTTTTCAGTATGTTTATCAGCGATAAAATTCTGCGTACCCGCGCGTTGCTGGGCTGCCTGACCTTT   6120
GGGTGTCAACAACCCAAGGCAAAAGTCATACAAATAGTCGCTATTTTAAGACGCATGGGCGCGCAACGACCCGACGGACTGGAAA
  195        200        205        210        215        220
T H S C W V P F S V C L S A I K F C V P A R C W A A * P L
```
ygaY

Fragment proU12_NlaIII_PT

```
                                          ygaY_RT-qPCR_2.fwd
                                          GCCGCTCCACCTTTTAACTACAG
GCCAATTTCAGCATTCTCTGGACCTCAATGGCCTTTTTGCTTGCCGCTCCACCTTTTAACTACAGCGATGGTGTCATTGGTCTGT   6205
CGGTTAAAGTCGTAAGAGACCTGGAGTTACCGGAAAAACGAACGGCGAGGTGGAAAATTGATGTCGCTACCACAGTAACCAGACA
    225        230        235        240        245        250
P I S A F S G P Q W P F C L P L H L L T T A M V S L V C
```
ygaY

Fragment proU12_NlaIII_PT

ygaY2

```
                                                                     ProU12_NlaIII_PT
                                                                     gcaccacaccacaac
TTGGACTTGCGGGAGCTGCCGGAGCGTTGGGCGCTCGTCCGGCGGGCGGTTTTGCCGATAAGGGCAAATCGCACCACACCACAAC   6290
AACCTGAACGCCCTCGACGGCCTCGCAACCCGCGAGCAGGCCGCCCGCCAAAACGGCTATTCCCGTTTAGCGTGGTGTGGTGTTG
      255        260        265        270        275
L D L R E L P E R W A L V R R A V L P I R A N R T T P Q
```
ygaY

Fragment proU12_NlaIII_PT

ygaY2

```
                                                               TATTCCCGTTTAGCGTGGTGTGG
                                                               ygaY_RT-qPCR_2.rev
```

```
ProU12_NlaIII_PT
tttc                                NlaIII
TTTCGGTCTGCTGCTGCTATTACTTTCATGGCTGGCGATCTGGTTTGGTCACACTTCCGTACTGGCGTTGATTATCGGAATCCTG   6375
AAAGCCAGACGACGACGATAATGAAAGTACCGACCGCTAGACCAAACCAGTGTGAAGGCATGACCGCAACTAATAGCCTTAGGAC
  280        285        290        295        300        305
L S V C C C Y Y F H G W R S G L V T L P Y W R * L S E S W
```
ygaY

Fragment proU12_NlaIII_PT | Fragment proU14_NlaIII_PT

ProU14_NlaIII_PT
GTGCATATCACTAACCAGACGGTAATTTATCG

GTGCTGGATCTCACCGTGCAGGGCGTGCATATCACTAACCAGACGGTAATTTATCGGATACATCCTGATGCGCGCAATCGCCTGA 6460
CACGACCTAGAGTGGCACGTCCCGCACGTATAGTGATTGGTCTGCCATTAAATAGCCTATGTAGGACTACGCGCGTTAGCGGACT

310 315 320 325 330 335
C W I S P C R A C I S L T R R * F I G Y I L M R A I A *
ygaY
Fragment proU14_NlaIII_PT

NlaIII NlaIII
CCGCAGGTTACATGACTAGCTACTTTATTGGCGGTGCCGCCGGTTCGCTAATTTCAGCCTCAGCCTGGCAACATGGCGGTTGGGC 6545
GGCGTCCAATGTACTGATCGATGAAATAACCGCCACGGCGGCCAAGCGATTAAAGTCGGAGTCGGACCGTTGTACCGCCAACCCG

340 345 350 355 360
P Q V T * L A T L L A V P P V R * F Q P Q P G N M A V G
ygaY
Fragment proU14_NlaIII_PT
Fragment proU15_NlaIII_PT

TGGCGTTTGTCTGGCTGGCGCGACGATTGCCCTGGTTAACTTACTGGTCTGGTGGCGAGGTTTTCATCGTCAGGAAGCCGCAAAT 6630
ACCGCAAACAGACCGACCGCGCTGCTAACGGGACCAATTGAATGACCAGACCACCGCTCCAAAAGTAGCAGTCCTTCGGCGTTTA

365 370 375 380 385 390
L A F V W L A R R L P W L T Y W S G G E V F I V R K P Q I
ygaY
Fragment proU15_NlaIII_PT

TAAGCAATCAGCAACCGTTTTCGTGGCTTTACACTTATAAGGGTGTTAAGAAGCCCATCAGTCTGATAAGGTTAAGATATTCATT 6715
ATTCGTTAGTCGTTGGCAAAAGCACCGAAATGTGAATATTCCCACAATTCTTCGGGTAGTCAGACTATTCCAATTCTATAAGTAA

K Q S A T V F V A L H L *
(in frame with ygaY)
ygaY
Fragment proU15_NlaIII_PT

ProU15_NlaIII_PT
CAATCGTTAAGCGTACACTCTATGGAAAGC

CAGTCTATTTATAATATTAACAATCGTTAAGCGTACACTCTATGGAAAGCCCTACTCCACAGCCTGCTCCTGGTTCGGCGACCTT 6800
GTCAGATAAATATTATAATTGTTAGCAATTCGCATGTGAGATACCTTTCGGGATGAGGTGTCGGACGAGGACCAAGCCGCTGGAA

1 5 10 15
M E S P T P Q P A P G S A T F
ygaZ
Fragment proU15_NlaIII_PT

NlaIII
CATGGAAGGATGCAAAGACAGTTTACCGATTGTTATTAGTTATATTCCGGTGGCCTTTGCGTTCGGTCTGAATGCGACCCGTCTG 6885
GTACCTTCCTACGTTTCTGTCAAATGGCTAACAATAATCAATATAAGGCCACCGGAAACGCAAGCCAGACTTACGCTGGGCAGAC

20 25 30 35 40
M E G C K D S L P I V I S Y I P V A F A F G L N A T R L
ygaZ
Fragment proU15_NlaIII_PT

GGATTCTCTCCTCTCGAAAGCGTTTTTTTCTCCTGCATCATTTATGCAGGCGCGAGCCAGTTCGTCATTACCGCGATGCTGGCAG 6970
CCTAAGAGAGGAGAGCTTTCGCAAAAAAAGAGGACGTAGTAAATACGTCCGCGCTCGGTCAAGCAGTAATGGCGCTACGACCGTC

45 50 55 60 65 70
G F S P L E S V F F S C I I Y A G A S Q F V I T A M L A
ygaZ

NlaIII NlaIII
CCGGGAGTAGTTTGTGGATTGCTGCACTGACCGTCATGGCAATGGATGTTCGCCATGTGTTGTATGGCCCGTCACTGCGTAGCCG 7055
GGCCCTCATCAAACACCTAACGACGTGACTGGCAGTACCGTTACCTACAAGCGGTACACAACATACCGGGCAGTGACGCATCGGC

75 80 85 90 95 100
A G S S L W I A A L T V M A M D V R H V L Y G P S L R S R
ygaZ

End (7069)
TATTATTCAGCGTC 3′
7069
ATAATAAGTCGCAG 5′

I I Q R
ygaZ

	Feature	Location	Size		⇄	Type
✓	**nrdE**	1 .. 632	632 bp	■	→	CDS

/codon_start = 1
/transl_table = 11 (Bacterial, Archaeal and Plant Plastid)
/gene = nrdE
/gene_synonym = ECK2669; JW2650
/locus_tag = b2675
/product = ribonucleoside-diphosphate reductase 2, alpha subunit
/function = enzyme; Central intermediary metabolism: Nucleotide interconversions
/EC_number = 1.17.4.1
/db_xref = GI: 16130589
/db_xref = ASAP: ABE-0008806
/db_xref = UniProtKB/Swiss-Prot: P39452
/db_xref = EcoGene: EG20257
/db_xref = GeneID: 947155
/protein_id = NP_417161.1
/translation = LRTSMLLARERGETFAGFKQSRYASGEYFSQYLQGNWQPKTAKVGELFTRSGITLPTREMWAQLRDDVMRYGIYNQNLQAVPPTGSISYINHATSSIHPIVAKVEIRKEGKTGRVYYPAPFMTNENLA LYQDAYEIGAEKIIDTYAEATRHVDQGLSLTLFFPDTATTRDINKAQIYAWRKGIKTLYYIRLRQMALEGTEIEGCVSCAL*
209 amino acids = 23,8 kDa

✓	**Fragment proU17_NlaIII_PT**	1 .. 279	279 bp	■	⊢⊣	misc_feature
✓	**Fragment proU16_NlaIII_PT**	280 .. 456	177 bp	■	⊢⊣	misc_feature
✓	**Fragment proU13_NlaIII_PT**	457 .. 900	444 bp	■	⊢⊣	misc_feature
✓	**nrdF**	642 .. 1601	960 bp	■	→	CDS

/product = ribonucleoside-diphosphate reductase 2, beta subunit, ferritin-like protein
/translation = MKLSRISAINWNKISDDKDLEVWNRLTSNFWLPEKVPLSNDIPAWQTLTVVEQQLTMRVFTGLTLLDTLQNVIGAPSLMPDALTPHEEAVLSNISFMEAVHARSYSSIFSTLCQTKDVDAAYAWSEEN APLQRKAQIIQQHYRGDDPLKKKIASVFLESFLFYSGFWLPMYFSSRGKLTNTADLIRLIIRDEAVHGYYIGYKYQKNMEKISLGQREELKSFAFDLLLELYDNELQYTDELYAETPWADDVKAFLCY NANKALMNLGYEPLFPAEMAEVNPAILAALSPNADENHDFFSGSGSSYVMGKAVETEDEDWNF*
319 amino acids = 36,4 kDa

✓	**nrdF1**	712 .. 840	129 bp	■	⊢⊣	misc_feature
✓	**Fragment proU1_NlaIII_PT**	946 .. 1262	317 bp	■	⊢⊣	misc_feature
✓	**Fragment proU2_NlaIII_PT**	1263 .. 1840	578 bp	■	⊢⊣	misc_feature
✓	**nrdF2**	1457 .. 1557	101 bp	■	⊢⊣	misc_feature
✓	**Intrinsically curved region**	1536 .. 1815	280 bp	■	⊢⊣	misc_feature

/note = This region is predicted to be a curved DNA sequence. *In vitro* studies have shown that H-NS exhibits a preferential binding to this site [1]. *In vivo* studies do not corroborate this observation [2].

[1] Tanaka, K. I., Muramatsu, S., Yamada, H., & Mizuno, T. (1991). Systematic characterization of curved DNA segments randomly cloned from Escherichia coli and their functional significance. *Molecular and General Genetics MGG*, *226*(3), 367-376.

[2] Lucht, J.M., Dersch, P., Kempf, B. and Bremer, E., 1994. Interactions of the nucleoid-associated DNA-binding protein H-NS with the regulatory region of the osmotically controlled proU operon of Escherichia coli. Journal of Biological Chemistry, 269(9), pp.6578-6578.

✓	**NRE**	1643 .. 3075	1433 bp	■	⊢⊣	misc_feature

/note = Dattananda,C.S.,Rajkumari,K. and Gowrishankar,J. (1991) Multiple mechanisms contribute toosmotic inducibility of proU operon expression in Escherichia coli:Demonstration of two osmoresponsive promoters and of a negative regulatoryelement within the first structural gene. *J. Bacteriol.*,10.1128/jb. 173.23.7481-7490.1991.

✓	**URE**	1666 .. 1848	183 bp	■	⊢⊣	regulatory

/regulatory_class = silencer

✓	**H-NS bound region**	1666 .. 1685	20 bp	■	⊢⊣	misc_feature

/note = In vitro DNase-I protection assay. Fig. 6 in [1]

[1] Lucht, J.M., Dersch, P., Kempf, B. and Bremer, E., 1994. Interactions of the nucleoid-associated DNA-binding protein H-NS with the regulatory region of the osmotically controlled proU operon of Escherichia coli. *Journal of Biological Chemistry*, *269*(9), pp.6578-6578.

✓	**H-NS bound region**	1697 .. 1718	22 bp	■	⊢⊣	misc_feature

/note = In vitro DNase-I protection assay. Fig. 6 in [1]

[1] Lucht, J.M., Dersch, P., Kempf, B. and Bremer, E., 1994. Interactions of the nucleoid-associated DNA-binding protein H-NS with the regulatory region of the osmotically controlled proU operon of Escherichia coli. Journal of Biological Chemistry, 269(9), pp.6578-6578.

✓	**σS ProU TSS**	1704 .. 1705	2 bp	■	⊢⊣	misc_feature

/note = σS ProU TSS between the two bases [1].

[1] Dattananda, C. S., Rajkumari, K., & Gowrishankar, J. (1991). Multiple mechanisms contribute to osmotic inducibility of proU operon expression in Escherichia coli: demonstration of two osmoresponsive promoters and of a negative regulatory element within the first structural gene. *Journal of bacteriology*, *173*(23), 7481-7490.

✓	**H-NS bound region**	1721 .. 1749	29 bp	■	⊢⊣	misc_feature

/note = In vitro DNase-I protection assay. Fig. 6 in [1]

[1] Lucht, J.M., Dersch, P., Kempf, B. and Bremer, E., 1994. Interactions of the nucleoid-associated DNA-binding protein H-NS with the regulatory region of the osmotically controlled proU operon of Escherichia coli. Journal of Biological Chemistry, 269(9), pp.6578-6578.

✓	**proU1**	1747 .. 1872	126 bp	■	⊢⊣	misc_feature

/note = RT-qPCR proU1 amplicon

✓	**H-NS bound region**	1755 .. 1784	30 bp	■	⊢⊣	misc_feature

/note = In vitro DNase-I protection assay. Fig. 6 in [1]

[1] Lucht, J.M., Dersch, P., Kempf, B. and Bremer, E., 1994. Interactions of the nucleoid-associated DNA-binding protein H-NS with the regulatory region of the osmotically controlled proU operon of Escherichia coli. Journal of Biological Chemistry, 269(9), pp.6578-6578.

	Feature	Location	Size	Type
✓	**H-NS bound region**	1791 .. 1819	29 bp	misc_feature

/note = In vitro DNase-I protection assay. Fig. 6 in [1]

[1] Lucht, J.M., Dersch, P., Kempf, B. and Bremer, E., 1994. Interactions of the nucleoid-associated DNA-binding protein H-NS with the regulatory region of the osmotically controlled proU operon of Escherichia coli. Journal of Biological Chemistry, 269(9), pp.6578-6578.

	Feature	Location	Size	Type
✓	**H-NS bound region**	1830 .. 1851	22 bp	misc_feature

/note = In vitro DNase-I protection assay. Fig. 6 in [1]

[1] Lucht, J.M., Dersch, P., Kempf, B. and Bremer, E., 1994. Interactions of the nucleoid-associated DNA-binding protein H-NS with the regulatory region of the osmotically controlled proU operon of Escherichia coli. Journal of Biological Chemistry, 269(9), pp.6578-6578.

	Feature	Location	Size	Type
✓	**EMSA_fragment**	1841 .. 2134	294 bp	misc_feature
✓	**Fragment proU3_NlaIII_PT**	1841 .. 1957	117 bp	misc_feature
✓	**NT644_DRE**	1855 .. 2071	217 bp	misc_feature

/note = DRE Sequence in NT644:

AGGGGTTGCCTCAGATTCTCAGTATGTTAGGGTAGAAGCAAGTGACGCTTTCCATTGGGTAGTACATCGACATAGACAAATAAAGGAATCTTTCTATTGCATGGCAATCAAACTCGAGATCAAGAAC
CTCTACAAGATCTTCGGCGAGCATCCACAGCGAGCGTTCAAGTACATCGAGCAGGGGCTCTCGAAGGAGCAGATCCTGGAGAAGACGGGG

	Feature	Location	Size	Type
✓	**DRE**	1855 .. 2071	217 bp	regulatory

▶ 5 segments

/note = H-NS nucleation sites (Green) described in [1].

[1] Bouffartigues, E., Buckle, M., Badaut, C., Travers, A., & Rimsky, S. (2007). H-NS cooperative binding to high-affinity sites in a regulatory element results in transcriptional silencing. *Nature structural & molecular biology*, *14*(5), 441-448.

/regulatory_class = silencer

	Feature	Location	Size	Type
✓	**H-NS bound region (Weak binding)**	1856 .. 1873	18 bp	misc_feature

/note = In vitro DNase-I protection assay. Fig. 6 in [1]

[1] Lucht, J.M., Dersch, P., Kempf, B. and Bremer, E., 1994. Interactions of the nucleoid-associated DNA-binding protein H-NS with the regulatory region of the osmotically controlled proU operon of Escherichia coli. Journal of Biological Chemistry, 269(9), pp.6578-6578.

	Feature	Location	Size	Type
✓	**-35 promoter sequence**	1860 .. 1865	6 bp	misc_feature

/note = Determined from Fig. 6 in [1]

[1] Lucht, J.M., Dersch, P., Kempf, B. and Bremer, E., 1994. Interactions of the nucleoid-associated DNA-binding protein H-NS with the regulatory region of the osmotically controlled proU operon of Escherichia coli. Journal of Biological Chemistry, 269(9), pp.6578-6578.

	Feature	Location	Size	Type
✓	**IHF binding site**	1862 .. 1919	58 bp	misc_feature

/note = Khodr,A., Fairweather,V.,Bouffartigues,E. and Rimsky,S. (2015) IHF is a trans-acting factor implicatedin the regulation of the proU P2 promoter. *FEMS Microbiol. Lett.*, 10.1093/femsle/fnu049.

	Feature	Location	Size	Type
✓	**-10 promoter sequence**	1882 .. 1887	6 bp	misc_feature

/note = Determined from Fig. 6 in [1]

[1] Lucht, J.M., Dersch, P., Kempf, B. and Bremer, E., 1994. Interactions of the nucleoid-associated DNA-binding protein H-NS with the regulatory region of the osmotically controlled proU operon of Escherichia coli. Journal of Biological Chemistry, 269(9), pp.6578-6578.

	Feature	Location	Size	Type
✓	**Initial IHF protection site**	1883 .. 1914	32 bp	misc_feature

/note = Khodr,A., Fairweather,V.,Bouffartigues,E. and Rimsky,S. (2015) IHF is a trans-acting factor implicatedin the regulation of the proU P2 promoter. *FEMS Microbiol. Lett.*, 10.1093/femsle/fnu049.

	Feature	Location	Size	Type
✓	**H-NS bound region**	1888 .. 1909	22 bp	misc_feature

/note = In vitro DNase-I protection assay. Fig. 6 in [1]

[1] Lucht, J.M., Dersch, P., Kempf, B. and Bremer, E., 1994. Interactions of the nucleoid-associated DNA-binding protein H-NS with the regulatory region of the osmotically controlled proU operon of Escherichia coli. Journal of Biological Chemistry, 269(9), pp.6578-6578.

	Feature	Location	Size	Type
✓	**σ70 ProU TSS**	1894 .. 1895	2 bp	misc_feature

/note = σ70 proU TSS (between the two bases); determined from Fig. 6 in [1]

[1] Lucht, J.M., Dersch, P., Kempf, B. and Bremer, E., 1994. Interactions of the nucleoid-associated DNA-binding protein H-NS with the regulatory region of the osmotically controlled proU operon of Escherichia coli. Journal of Biological Chemistry, 269(9), pp.6578-6578.

	Feature	Location	Size	Type
✓	**H-NS bound region**	1912 .. 1924	13 bp	misc_feature

/note = In vitro DNase-I protection assay. Fig. 6 in [1]

[1] Lucht, J.M., Dersch, P., Kempf, B. and Bremer, E., 1994. Interactions of the nucleoid-associated DNA-binding protein H-NS with the regulatory region of the osmotically controlled proU operon of Escherichia coli. Journal of Biological Chemistry, 269(9), pp.6578-6578.

	Feature	Location	Size	Type
✓	**Shine Dalgarno sequence**	1927 .. 1957	31 bp	misc_feature

/note = http://regulondb.ccg.unam.mx/gene?term=ECK120000762&organism=ECK12&format=jsp&type=gene

	Feature	Location	Size	Type
✓	**H-NS bound region**	1928 .. 1949	22 bp	misc_feature

/note = In vitro DNase-I protection assay. Fig. 6 in [1]

[1] Lucht, J.M., Dersch, P., Kempf, B. and Bremer, E., 1994. Interactions of the nucleoid-associated DNA-binding protein H-NS with the regulatory region of the osmotically controlled proU operon of Escherichia coli. Journal of Biological Chemistry, 269(9), pp.6578-6578.

	Feature	Location	Size		⇄	Type
✓	**proV**	1955 .. 3157	1203 bp	■	→	CDS

/transl_table = 11 (Bacterial, Archaeal and Plant Plastid)
/gene = proV
/gene_synonym = ECK2671; JW2652; osrA; proU
/locus_tag = b2677
/product = glycine betaine transporter subunit
/function = transport; Transport of small molecules: Amino acids, amines
/note = ATP-binding component of transport system for glycine, betaine and proline
/db_xref = GI: 16130591
/db_xref = ASAP: ABE-0008814
/db_xref = UniProtKB/Swiss-Prot: P14175
/db_xref = EcoGene: EG10771
/db_xref = GeneID: 947148
/protein_id = NP_417163.1
/translation = MAIKLEIKNLYKIFGEHPQRAFKYIEQGLSKEQILEKTGLSLGVKDASLAIEEGEIFVIMGLSGSGKSTMVRLLNRLIEPTRGQVLIDGVDIAKISDAELREVRRKKIAMVFQSFALMPHMTVLDNTA
FGMELAGINAEERREKALDALRQVGLENYAHSYPDELSGGMRQRVGLARALAINPDILLMDEAFSALDPLIRTEMQDELVKLQAKHQRTIVFISHDLDEAMRIGDRIAIMQNGEVVQVGTPDEILNNP
ANDYVRTFFRGVDISQVFSAKDIARRTPNGLIRKTPGFGPRSALKLLQDEDREYGYVIERGNKFVGAVSIDSLKTALTQQQGLDAALIDAPLAVDAQTPLSELLSHVGQAPCAVPVVDEDQQYVGIIS
KGMLLRALDREGVNNG*
400 amino acids = 44,2 kDa

✓	**Fragment proU4_NlaIII_PT**	1958 .. 2134	177 bp	■	⊢⊣	misc_feature
✓	**H-NS bound region (Strong binding)**	1958 .. 2003	46 bp	■	⊢⊣	misc_feature

/note = In vitro DNase-I protection assay. Fig. 6 in [1]

[1] Lucht, J.M., Dersch, P., Kempf, B. and Bremer, E., 1994. Interactions of the nucleoid-associated DNA-binding protein H-NS with the regulatory region of the osmotically controlled proU operon of Escherichia coli. Journal of Biological Chemistry, 269(9), pp.6578-6578.

✓	**H-NS bound region**	2014 .. 2035	22 bp	■	⊢⊣	misc_feature

/note = In vitro DNase-I protection assay. Fig. 6 in [1]

[1] Lucht, J.M., Dersch, P., Kempf, B. and Bremer, E., 1994. Interactions of the nucleoid-associated DNA-binding protein H-NS with the regulatory region of the osmotically controlled proU operon of Escherichia coli. Journal of Biological Chemistry, 269(9), pp.6578-6578.

✓	**H-NS bound region**	2043 .. 2075	33 bp	■	⊢⊣	misc_feature

/note = In vitro DNase-I protection assay. Fig. 6 in [1]

[1] Lucht, J.M., Dersch, P., Kempf, B. and Bremer, E., 1994. Interactions of the nucleoid-associated DNA-binding protein H-NS with the regulatory region of the osmotically controlled proU operon of Escherichia coli. Journal of Biological Chemistry, 269(9), pp.6578-6578.

✓	**conserved RNase III degradation site**	2100 .. 2189	90 bp	■	⊢⊣	misc_feature
✓	**Fragment proU5_NlaIII_PT**	2135 .. 2641	507 bp	■	⊢⊣	misc_feature
✓	**proV1**	2240 .. 2339	100 bp	■	⊢⊣	misc_feature

/note = proV1 RT-qPCR amplicon

✓	**Fragment proU6_NlaIII_PT**	2642 .. 3041	400 bp	■	⊢⊣	misc_feature
✓	**proV2**	2722 .. 2837	116 bp	■	⊢⊣	misc_feature

/note = proV2 RT-qPCR amplicon

✓	**Fragment proU7_NlaIII_PT**	3042 .. 3710	669 bp	■	⊢⊣	misc_feature
✓	**proW**	3150 .. 4214	1065 bp	■	→	CDS

/transl_table = 11 (Bacterial, Archaeal and Plant Plastid)
/gene = proW
/gene_synonym = ECK2672; JW2653; osrA; proU
/locus_tag = b2678
/product = glycine betaine transporter subunit
/function = transport; Transport of small molecules: Amino acids, amines
/note = high-affinity transport system for glycine betaine and proline; membrane component of ABC superfamily
/db_xref = GI: 16130592
/db_xref = ASAP: ABE-0008816
/db_xref = UniProtKB/Swiss-Prot: P14176
/db_xref = EcoGene: EG10772
/db_xref = GeneID: 947145
/protein_id = NP_417164.1
/translation = MADQNNPWDTTPAADSAAQSADAWGTPTTAPTDGGGADWLTSTPAPNVEHFNILDPFHKTLIPLDSWVTEGIDWVVTHFRPVFQGVRVPVDYILNGFQQLLLGMPAPVAIIVFALIAWQISGVGMGVA
TLVSLIAIGAIGAWSQAMVTLALVLTALLFCIVIGLPLGIWLARSPRAAKIIRPLLDAMQTTPAFVYLVPIVMLFGIGNVPGVVVTIIFALPPIIRLTILGINQVPADLIEASRSFGASPRQMLFKVQ
LPLAMPTIMAGVNQTLMLALSMVVIASMIAVGGLGQMVLRGIGRLDMGLATVGGVGIVILAIILDRLTQAVGRDSRSRGNRRWYTTGPVGLLTRPFIK*
354 amino acids = 37,6 kDa

✓	**proW1**	3380 .. 3500	121 bp	■	⊢⊣	misc_feature

/note = proW1 RT-qPCR amplicon

✓	**Hairpin encoding sequence?**	3509 .. 3522	14 bp	■	⊢⊣	misc_feature

/note = Stem (5 bp) -- Loop (4 bp) -- Stem (5 bp)

✓	**Termination site**	3536 .. 3536	1 bp	■	⊢⊣	misc_feature

/note = According to Term-Seq experiments, this is the base at which most transcription terminates

(David C. Grainger)

Feature		Location	Size			Type
✓	**rG4 site**	3571 .. 3620	50 bp	■	⊢⊣	misc_feature

/note = Class: 2 quartet
Reverse Transcriptase Stalling: 0.276786
Strand: +

Reference: Shao, X., Zhang, W., Umar, M.I., Wong, H.Y., Seng, Z., Xie, Y., Zhang, Y., Yang, L., Kwok, C.K. and Deng, X., 2020. RNA G-Quadruplex structures mediate gene regulation in bacteria. *MBio*, *11*(1).

✓	**rG4 site**	3575 .. 3624	50 bp	■	⊢⊣	misc_feature

/note = Class: 2 quartet
Reverse Transcriptase Stalling: 0.003488
Strand: +

Reference: Shao, X., Zhang, W., Umar, M.I., Wong, H.Y., Seng, Z., Xie, Y., Zhang, Y., Yang, L., Kwok, C.K. and Deng, X., 2020. RNA G-Quadruplex structures mediate gene regulation in bacteria. *MBio*, *11*(1).

✓	**rG4 site**	3579 .. 3628	50 bp	■	⊢⊣	misc_feature

/note = Class: 2 quartet
Reverse Transcriptase Stalling: 0.002725
Strand: +

Reference: Shao, X., Zhang, W., Umar, M.I., Wong, H.Y., Seng, Z., Xie, Y., Zhang, Y., Yang, L., Kwok, C.K. and Deng, X., 2020. RNA G-Quadruplex structures mediate gene regulation in bacteria. *MBio*, *11*(1).

✓	**Fragment proU8_NlaIII_PT**	3753 .. 4542	790 bp	■	⊢⊣	misc_feature
✓	**proW2**	3895 .. 3994	100 bp	■	⊢⊣	misc_feature

/note = proW2 RT-qPCR amplicon

✓	**Hairpin encoding sequence?**	4224 .. 4242	19 bp	■	⊢⊣	misc_feature

/note = Stem (7 bp) -- Loop (5 bp) -- Stem (7 bp)

✓	**Termination site**	4247 .. 4247	1 bp	■	⊢⊣	misc_feature

/note = Base where most transcription terminates

✓	**proX**	4272 .. 5264	993 bp	■	→	CDS

/transl_table = 11 (Bacterial, Archaeal and Plant Plastid)
/gene = proX
/gene_synonym = ECK2673; JW2654; osrA; proU
/locus_tag = b2679
/product = glycine betaine transporter subunit
/function = transport; Osmotic adaptation
/note = high-affinity transport system for glycine betaine and proline; periplasmic-binding component of ABC superfamily
/db_xref = GI: 16130593
/db_xref = ASAP: ABE-0008818
/db_xref = UniProtKB/Swiss-Prot: P0AFM2
/db_xref = EcoGene: EG10773
/db_xref = GeneID: 947165
/protein_id = NP_417165.1
/translation = MRHSVLFATAFATLISTQTFAADLPGKGITVNPVQSTITEETFQTLLVSRALEKLGYTVNKPSEVDYNVGYTSLASGDATFTAVNWTPLHDNMYEAAGGDKKFYREGVFVNGAAQGYLIDKKTADQYK
ITNIAQLKDPKIAKLFDTNGDGKADLTGCNPGWGCEGAINHQLAAYELTNTVTHNQGNYAAMMADTISRYKEGKPVFYYTWTPYWVSNELKPGKDVVWLQVPFSALPGDKNADTKLPNGANYGFPVST
MHIVANKAWAEKNPAAAKLFAIMQLPVADINAQNAIMHDGKASEGDIQGHVDGWIKAHQQQFDGWVNEALAAQK*
330 amino acids = 36,0 kDa

✓	**proX1**	4676 .. 4775	100 bp	■	⊢⊣	misc_feature

/note = proX1 RT-qPCR amplicon

✓	**Fragment proU9_NlaIII_PT**	5043 .. 5154	112 bp	■	⊢⊣	misc_feature
✓	**proX2**	5045 .. 5148	104 bp	■	⊢⊣	misc_feature

/note = proX2 RT-qPCR amplicon

✓	**Fragment proU10_NlaIII_PT**	5155 .. 5349	195 bp	■	⊢⊣	misc_feature
✓	**Intrinsic terminator?**	5265 .. 5307	43 bp	■	⊢⊣	misc_feature

/note = If this is the intrinsic terminator, a hairpin sequence is expected to be detected here. But, there is nothing obvious. Perhaps a Rho dependent terminator sequence?

✓	**repeat region**	5271 .. 5354	84 bp	■	⊢⊣	repeat_region

/note = REP193 (repetitive extragenic palindromic) element; contains 2 REP sequences

✓	**Termination site**	5308 .. 5308	1 bp	■	⊢⊣	misc_feature

/note = Base where most transcription terminates

	Feature	Location	Size		⇄	Type
✓	**ygaY**	5456 .. 6633	1178 bp	■	→	CDS

/transl_table = 11 (Bacterial, Archaeal and Plant Plastid)
/gene = ygaY
/gene_synonym = ECK2674; JW2655; ygaX
/locus_tag = b2681
/note = predicted transporter (pseudogene);putative transport; Not classified; putative transport protein
/db_xref = ASAP: ABE-0008822
/db_xref = ASAP: ABE-0008824
/db_xref = UniProtKB/Swiss-Prot: P76628
/db_xref = EcoGene: EG13527
/db_xref = GeneID: 2847696
/translation = MTKPNHELSPALIVLMSIATGLAVASNYYAQPLLDTIARNFSLSASSAGFIVTAAQLGYAAGLLFLVPLGDMFERRRLIVSMTLLAAC*LPPAVSRWR**SSVRH*PVYSQSWHKFWFRWQRRWLHRI
NAAKWLALL*AVCCWGSCWHGQLPDCWRISAAGAPSFGLLRC*WH*WRWHYGVVCHK*NQKPTSTTHSCWVPFSVCLSAIKFCVPARCWAA*PLPISAFSGPQWPFCLPLHLLTTAMVSLVCLDLREL
PERWALVRRAVLPIRANRTTPQLSVCCCYYFHGWRSGLVTLPYWR*LSESWCWISPCRACISLTRR*FIGYILMRAIA*PQVT*LATLLAVPPVR*FQPQPGNMAVGLAFVWLARRLPWLTYWSGGEV
FIVRKPQI
392 codons (14 internal stop codons)

	Feature	Location	Size		⇄	Type
✓	**Fragment proU11_NlaIII_PT**	5475 .. 5718	244 bp	■	⊢⊣	misc_feature
✓	**ygaY1**	5575 .. 5710	136 bp	■	⊢⊣	misc_feature

/note = ygaY1 RT-qPCR amplicon

	Feature	Location	Size		⇄	Type
✓	**Fragment proU12_NlaIII_PT**	5719 .. 6320	602 bp	■	⊢⊣	misc_feature
✓	**ygaY2**	6163 .. 6285	123 bp	■	⊢⊣	misc_feature

/note = ygaY2 RT-qPCR amplicon

	Feature	Location	Size		⇄	Type
✓	**Fragment proU14_NlaIII_PT**	6321 .. 6474	154 bp	■	⊢⊣	misc_feature
✓	**Fragment proU15_NlaIII_PT**	6536 .. 6804	269 bp	■	⊢⊣	misc_feature
✓	**ygaZ**	6757 .. 7069	313 bp	■	→	CDS

/transl_table = 11 (Bacterial, Archaeal and Plant Plastid)
/gene = ygaZ
/gene_synonym = ECK2676; JW2657
/locus_tag = b2682
/product = probable L-valine exporter, norvaline resistance
/db_xref = GI: 16130594
/db_xref = ASAP: ABE-0008829
/db_xref = UniProtKB/Swiss-Prot: P76630
/db_xref = EcoGene: EG13528
/db_xref = GeneID: 945093
/protein_id = NP_417167.1
/translation = MESPTPQPAPGSATFMEGCKDSLPIVISYIPVAFAFGLNATRLGFSPLESVFFSCIIYAGASQFVITAMLAAGSSLWIAALTVMAMDVRHVLYGPSLRSRIIQR
104 amino acids = 11,1 kDa

Primer	Length	Binding Sites	⇄	Tm	Date Added
**ProU17_NlaIII_PT**	28-mer	210 .. 237	→	61°C	
**ProU16_NlaIII_PT**	27-mer	386 .. 412	→	61°C	
**nrdF_RT-qPCR_1.fwd**	20-mer	712 .. 731	→	58°C	
**nrdF_RT-qPCR_1.rev**	20-mer	821 .. 840	←	64°C	
**ProU13_NlaIII.pt**	21-mer	842 .. 862	→	57°C	
**ProU1_NlaIII_PT**	18-mer	1212 .. 1229	→	57°C	
**nrdF_RT-qPCR_2.fwd**	23-mer	1457 .. 1479	→	61°C	
**nrdF_RT-qPCR_2.rev**	23-mer	1535 .. 1557	←	60°C	
**proU_RT-qPCR_1.fwd**	33-mer	1747 .. 1779	→	60°C	
**ProU2_NlaIII_PT**	19-mer	1778 .. 1796	→	58°C	
**proU3_NlaIII.fwd**	22-mer	1841 .. 1862	→	61°C	
**DRE_fwd**	59-mer	1849 .. 1907	→	70°C	17 Jan 2021
**proU_RT-qPCR_1.rev**	23-mer	1850 .. 1872	←	61°C	
**proU3_NlaIII.probe.IC**	30-mer	1853 .. 1882	←	64°C	
**ProU3_NlaIII_PT**	24-mer	1862 .. 1885	→	57°C	
**ProU3_NlaIII_PT.probe**	36-mer	1895 .. 1930	←	60°C	
**proU3_NlaIII_PT.probe.2**	38-mer	1920 .. 1957	←	60°C	
**proU3_NlaIII.rev**	36-mer	1922 .. 1957	←	60°C	
**ProU4_NlaIII_PT**	19-mer	2064 .. 2082	→	58°C	

✓ **ProU17_NlaIII_PT** — 28-mer — 210 .. 237 → — 61°C
/sequence = CGCTACGGCATATACAACCAGAATCTTC
46% GC / 8501,6 Da

✓ **ProU16_NlaIII_PT** — 27-mer — 386 .. 412 → — 61°C
/sequence = GCTGTATCAGGACGCTTACGAAATTGG
48% GC / 8339,5 Da

✓ **nrdF_RT-qPCR_1.fwd** — 20-mer — 712 .. 731 → — 58°C
/sequence = ATCGCCTGACCAGCAATTTC
50% GC / 6037,0 Da

✓ **nrdF_RT-qPCR_1.rev** — 20-mer — 821 .. 840 ← — 64°C
/sequence = CGAGCAGCGTCAGGCCAGTA
65% GC / 6152,0 Da

✓ **ProU13_NlaIII.pt** — 21-mer — 842 .. 862 → — 57°C
/sequence = cacgctgcaaaatgttatcgg
48% GC / 6430,3 Da

✓ **ProU1_NlaIII_PT** — 18-mer — 1212 .. 1229 → — 57°C
/sequence = gatgaagcagtccacggt
56% GC / 5548,7 Da

✓ **nrdF_RT-qPCR_2.fwd** — 23-mer — 1457 .. 1479 → — 61°C
/sequence = CGCAGAAATGGCGGAAGTGAATC
52% GC / 7146,7 Da

✓ **nrdF_RT-qPCR_2.rev** — 23-mer — 1535 .. 1557 ← — 60°C
/sequence = TCACATAAGAGGAGCCTGAACCG
52% GC / 7066,7 Da

✓ **proU_RT-qPCR_1.fwd** — 33-mer — 1747 .. 1779 → — 60°C
/sequence = CGCTATCTTTGACAAAAAATATCAACTTTCTCG
33% GC / 10.030,6 Da
/note = The proU operon can be expressed from two Transcription Start Sites: σ70 proU TSS and σS proU TSS.
The proU_RT-qPCR_1 primer pair is positioned between these promoter sites. This way, it can be used to quantify the relative contribution of each promoter to the expression of the operon

✓ **ProU2_NlaIII_PT** — 19-mer — 1778 .. 1796 → — 58°C
/sequence = cgatttgctctcagccctt
53% GC / 5705,8 Da

✓ **proU3_NlaIII.fwd** — 22-mer — 1841 .. 1862 → — 61°C
/sequence = CCACATTTGCCATCAGGGGTTG
55% GC / 6726,4 Da

✓ **DRE_fwd** — 59-mer — 1849 .. 1907 → — 70°C — 17 Jan 2021
/sequence = GCCATCAGGGGTTGCCTCAGATTCTCAGTATGTTAGGGTAGAAAAAAGTGACTATTTCC
44% GC / 18.239,9 Da

✓ **proU_RT-qPCR_1.rev** — 23-mer — 1850 .. 1872 ← — 61°C
/sequence = GAATCTGAGGCAACCCCTGATGG
57% GC / 7073,7 Da
/note = The proU operon can be expressed from two Transcription Start Sites: σ70 proU TSS and σS proU TSS.
The proU_RT-qPCR_1 primer pair is positioned between these promoter sites. This way, it can be used to quantify the relative contribution of each promoter to the expression of the operon

✓ **proU3_NlaIII.probe.IC** — 30-mer — 1853 .. 1882 ← — 64°C
/sequence = AACATACTGAGAATCTGAGGCAACCCCTGA
47% GC / 9193,1 Da

✓ **ProU3_NlaIII_PT** — 24-mer — 1862 .. 1885 → — 57°C
/sequence = gcctcagattctcagtatgttagg
46% GC / 7358,9 Da

✓ **ProU3_NlaIII_PT.probe** — 36-mer — 1895 .. 1930 ← — 60°C
/sequence = tctatgtcgatatattacccaatggaaatagtcact
33% GC / 11.017,3 Da

✓ **proU3_NlaIII_PT.probe.2** — 38-mer — 1920 .. 1957 ← — 60°C
/sequence = CATGCAATAGAAAGATTCCTTTATTTGTCTATGTCGAT
32% GC / 11.656,7 Da

✓ **proU3_NlaIII.rev** — 36-mer — 1922 .. 1957 ← — 60°C
/sequence = CATGCAATAGAAAGATTCCTTTATTTGTCTATGTCG
33% GC / 11.039,3 Da

✓ **ProU4_NlaIII_PT** — 19-mer — 2064 .. 2082 → — 58°C
/sequence = aaactgggctatcgcttgg
53% GC / 5843,9 Da

	Primer	Length		Binding Sites	⇄	Tm	Date Added
✓	**proU4_NlaIII_PT.2**	22-mer		2069 .. 2090	→	61°C	
	/sequence = GGGCTATCGCTTGGCGTAAAAG<br>55% GC / 6815,5 Da						
✓	**proV_RT-PCR_1.fwd**	17-mer		2240 .. 2256	→	63°C	
	/sequence = TCCGACGCCGAACTCCG<br>71% GC / 5116,4 Da						
✓	**proV_RT-PCR_1.rev**	20-mer		2320 .. 2339	←	60°C	
	/sequence = ACGCAGTATTGTCCAGCACG<br>55% GC / 6102,0 Da						
✓	**ProU5_NlaIII_PT**	21-mer		2580 .. 2600	→	56°C	
	/sequence = aattacaggcgaaacatcagc<br>43% GC / 6432,3 Da						
✓	**proV_RT-PCR_2.fwd**	28-mer		2722 .. 2749	→	61°C	
	/sequence = GGCGAATGATTATGTCCGTACCTTCTTC<br>46% GC / 8545,6 Da						
✓	**proV_RT-PCR_2.rev**	22-mer		2816 .. 2837	←	60°C	
	/sequence = CGAAGCCAGGGGTTTTACGAAT<br>50% GC / 6799,5 Da						
✓	**ProU6_NlaIII_PT**	19-mer		2931 .. 2949	→	56°C	
	/sequence = tcgattcgcttaaaaccgc<br>47% GC / 5747,8 Da						
✓	**proW_RT-PCR_1.fwd**	22-mer		3380 .. 3401	→	61°C	
	/sequence = CCATTTCCGTCCCGTCTTCCAG<br>59% GC / 6573,3 Da						
✓	**proW_RT-PCR_1.rev**	25-mer		3476 .. 3500	←	59°C	
	/sequence = GGCGATGAGAGCGAAAACGATAATC<br>48% GC / 7773,1 Da						
✓	**ProU7_NlaIII_PT**	19-mer		3649 .. 3667	→	57°C	
	/sequence = GGATATGGCTGGCGAGAAG<br>58% GC / 5957,9 Da						
✓	**proW_RT-PCR_2.fwd**	27-mer		3895 .. 3921	→	60°C	
	/sequence = GCCAGATGCTGTTCAAAGTTCAGTTAC<br>44% GC / 8274,5 Da						
✓	**proW_RT-PCR_2.rev**	23-mer		3972 .. 3994	←	62°C	
	/sequence = GCGATGACCACCATAGAAAGGGC<br>57% GC / 7091,7 Da						
✓	**ProU8_NlaIII_PT**	18-mer		4476 .. 4493	→	57°C	
	/sequence = gttggctacacctcgctt<br>56% GC / 5441,6 Da						
✓	**proX_RT-PCR_1.fwd**	28-mer		4676 .. 4703	→	62°C	
	/sequence = GAAAGATCCGAAGATCGCCAAACTGTTC<br>46% GC / 8590,7 Da						
✓	**proX_RT-PCR_1.rev**	20-mer		4756 .. 4775	←	61°C	
	/sequence = GTTGATCGCACCTTCGCAGC<br>60% GC / 6069,0 Da						
✓	**proX_RT-PCR_2.fwd**	23-mer		5045 .. 5067	→	62°C	
	/sequence = TATCGTTGCCAACAAAGCCTGGG<br>52% GC / 7048,6 Da						
✓	**ProU9_NlaIII_PT**	22-mer		5087 .. 5108	→	57°C	
	/sequence = agcgaaactgtttgccattatg<br>41% GC / 6749,5 Da						
✓	**proX_RT-PCR_2.rev**	27-mer		5122 .. 5148	←	61°C	
	/sequence = TAATGGCGTTCTGGGCGTTAATATCTG<br>44% GC / 8336,5 Da						
✓	**ProU10_NlaIII_PT**	19-mer		5276 .. 5294	→	57°C	
	/sequence = gcggataaggcgtttttgc<br>53% GC / 5874,9 Da						
✓	**ygaY_RT-qPCR_1.fwd**	22-mer		5575 .. 5596	→	60°C	
	/sequence = CTTTTCCCTTTCCGCCAGTTCG<br>55% GC / 6579,3 Da						
✓	**ProU11_NlaIII_PT**	21-mer		5660 .. 5680	→	56°C	
	/sequence = CTCGGTGATATGTTTGAACGC<br>48% GC / 6452,3 Da						

	Primer	Length		Binding Sites	⇄	Tm	Date Added
✓	**ygaY_RT-qPCR_1.rev**	25-mer		5686 .. 5710	←	58°C	
	/sequence = CAGTAAGGTCATCGAGACAATCAGG<br>48% GC / 7724,1 Da						
✓	**ygaY_RT-qPCR_2.fwd**	23-mer		6163 .. 6185	→	61°C	
	/sequence = GCCGCTCCACCTTTTAACTACAG<br>52% GC / 6919,5 Da						
✓	**ygaY_RT-qPCR_2.rev**	23-mer		6263 .. 6285	←	63°C	
	/sequence = GGTGTGGTGCGATTTGCCCTTAT<br>52% GC / 7092,7 Da						
✓	**ProU12_NlaIII_PT**	19-mer		6276 .. 6294	→	56°C	
	/sequence = gcaccacaccacaactttc<br>53% GC / 5661,7 Da						
✓	**ProU14_NlaIII_PT**	32-mer		6400 .. 6431	→	60°C	
	/sequence = GTGCATATCACTAACCAGACGGTAATTTATCG<br>41% GC / 9807,5 Da						
✓	**ProU15_NlaIII_PT**	30-mer		6736 .. 6765	→	61°C	
	/sequence = CAATCGTTAAGCGTACACTCTATGGAAAGC<br>43% GC / 9199,1 Da						
