# Supplementary material for: The environmentally-regulated interplay between local three-dimensional chromatin organisation and transcription of proVWX in E. coli
Source: Nat Commun. 2023 Nov 17;14:7478. doi: 10.1038/s41467-023-43322-y (PMC10656529; doi:10.1038/s41467-023-43322-y)
Supplement: Supplementary file 11 — Reporting Summary [file 41467_2023_43322_MOESM11_ESM.pdf]

nature portfolio

Corresponding author(s): Remus T. Dame

Last updated by author(s): Sep 29, 2023

Reporting Summary

Nature Portfolio wishes to improve the reproducibility of the work that we publish. This form provides structure for consistency and transparency in reporting. For further information on Nature Portfolio policies, see our Editorial Policies and the Editorial Policy Checklist.

Statistics

For all statistical analyses, confirm that the following items are present in the figure legend, table legend, main text, or Methods section.

n/a	Confirmed	
☐	☒	The exact sample size (*n*) for each experimental group/condition, given as a discrete number and unit of measurement
☐	☒	A statement on whether measurements were taken from distinct samples or whether the same sample was measured repeatedly
☒	☐	The statistical test(s) used AND whether they are one- or two-sided<br>*Only common tests should be described solely by name; describe more complex techniques in the Methods section.*
☒	☐	A description of all covariates tested
☐	☒	A description of any assumptions or corrections, such as tests of normality and adjustment for multiple comparisons
☐	☒	A full description of the statistical parameters including central tendency (e.g. means) or other basic estimates (e.g. regression coefficient) AND variation (e.g. standard deviation) or associated estimates of uncertainty (e.g. confidence intervals)
☒	☐	For null hypothesis testing, the test statistic (e.g. *F*, *t*, *r*) with confidence intervals, effect sizes, degrees of freedom and *P* value noted<br>*Give P values as exact values whenever suitable.*
☒	☐	For Bayesian analysis, information on the choice of priors and Markov chain Monte Carlo settings
☒	☐	For hierarchical and complex designs, identification of the appropriate level for tests and full reporting of outcomes
☒	☐	Estimates of effect sizes (e.g. Cohen's *d*, Pearson's *r*), indicating how they were calculated

Our web collection on statistics for biologists contains articles on many of the points above.

Software and code

Policy information about availability of computer code

Data collection

All qPCR data was collected using the Bio-Rad CFX Manager™ (RRID:SCR_017251) Version 3.1 software. Bio-Rad CFX Manager™ (RRID:SCR_017251) Version 3.1 is an experiment setup and data analysis software for all CFX Real-Time PCR Detection Systems (Bio-Rad Laboratories, Inc., Hercules, California, U.S.A.)

Next-generation sequencing data was collected by the Utrecht Sequencing Facility (USEQ). USEQ is subsidized by the University Medical Center Utrecht and The Netherlands X-omics Initiative.

Sanger sequencing data was collected by BaseClear B.V., Leiden, The Netherlands.

Concentration measurements collected using the NanoDrop spectrophotometer were collected using the NanoDrop 2000/2000c software Version 1.6 (Thermo Fisher Scientific Inc.).

Concentration measurements collected using the Qubit fluorometer were collected using the software installed on the device (Thermo Fisher Scientific Inc.).

Agarose gels and polyacrylamide gels were imaged using GelDoc™ XR+ (Bio-Rad) with the Image Lab Version 6.0 software (Bio-Rad).

Data analysis	All qPCR data were analysed with Microsoft Excel (Microsoft 365). Graphs were prepared using Igor Pro 7 Version 7.05.<br><br>Hi-C data analysis were carried out as described in Hofmann, Andreas, and Dieter W. Heermann. "Processing and analysis of Hi-C data on bacteria." Bacterial Chromatin: Methods and Protocols (2018): 19-31.

For manuscripts utilizing custom algorithms or software that are central to the research but not yet described in published literature, software must be made available to editors and reviewers. We strongly encourage code deposition in a community repository (e.g. GitHub). See the Nature Portfolio guidelines for submitting code & software for further information.

Data

Policy information about availability of data

All manuscripts must include a data availability statement. This statement should provide the following information, where applicable:

- Accession codes, unique identifiers, or web links for publicly available datasets
- A description of any restrictions on data availability
- For clinical datasets or third party data, please ensure that the statement adheres to our policy

All data generated in this study have been deposited in the 4TU Repository (https://doi.org/10.4121/21065275). Hi-C data are also available from the NCBI GEO repository under accession number GSE214511 (https://www.ncbi.nlm.nih.gov/geo/query/acc.cgi?acc=GSE214511). The RT-qPCR and 3C-qPCR data generated in this study are also provided in the Supplementary Information files. Source data are provided as a Source Data file.

E. coli reference genomes U00096.2 (https://www.ebi.ac.uk/ena/browser/view/U00096) and NC_000913.3 (https://www.ncbi.nlm.nih.gov/nuccore/556503834) were used in this study.

Research involving human participants, their data, or biological material

Policy information about studies with human participants or human data. See also policy information about sex, gender (identity/presentation), and sexual orientation and race, ethnicity and racism.

Reporting on sex and gender	We have not performed research involving human participants, their data, or biological material.
Reporting on race, ethnicity, or other socially relevant groupings	We have not performed research involving human participants, their data, or biological material.
Population characteristics	We have not performed research involving human participants, their data, or biological material.
Recruitment	We have not performed research involving human participants, their data, or biological material.
Ethics oversight	We have not performed research involving human participants, their data, or biological material.

Note that full information on the approval of the study protocol must also be provided in the manuscript.

Field-specific reporting

Please select the one below that is the best fit for your research. If you are not sure, read the appropriate sections before making your selection.

☒ Life sciences ☐ Behavioural & social sciences ☐ Ecological, evolutionary & environmental sciences

For a reference copy of the document with all sections, see nature.com/documents/nr-reporting-summary-flat.pdf

Life sciences study design

All studies must disclose on these points even when the disclosure is negative.

Sample size	qPCR experiments were performed for four biological replicates. Each replicate was tested three times. The community-accepted standard for the number of biological replicates in qPCR experiments is at least three. Four biological replicates were selected to be beyond this margin. Each biological replicate is generally tested three times to ensure quantitation accuracy. It also allows pipetting errors, amplification failures, and outliers to be identified.<br><br>Hi-C experiments were performed for one culture. Biological replicates were not performed. The community accepted standard for the number of biological replicates in ChIP-Seq, RNA-Seq, 3C-Seq, and Hi-C experiments is at least two. We used the Hi-C experiments as a preliminary test to motivate our subsequent experiments. Hence, single biological samples were sufficient to indicate to us how the study should be pursued. We have placed this data in the supplementary information and referenced it in the main text as a motivation for the experiments we present in the main text.
Data exclusions	For qPCR experiments: Reactions with unreliable Cq values due to pipetting errors owing to the manual set-up of the experiment (identified as amplification outliers with either extremely high or extremely low Cq values compared to other technical replicates), or evaporation from improperly sealed wells (observed as a decrease in the reaction volume in the wells after the experiment was completed), were eliminated from analysis.

For the intercalator-based RT-qPCR experiment, melt curves were also used to check the specificity of amplification, and hence, the validity of the experiment. Wells that showed more than one melt curve peak indicating the presence of more than one amplification product, and wells that showed melt curves with a melting peak that differed by more than 0.5oC from the positive control sample indicating the amplification of a wrong product were eliminated.

Data exclusion criteria were defined before the experiment was performed.

All results (before data exclusion) have been provided in the supplementary data files. The data files also mark which data was excluded from the analysis and why.

Replication	qPCR experiments were performed for four biological replicates. Each replicate was tested three times. The comparability of the results from all four biological replicates was considered to indicate reproducibility of the findings.<br><br>RT-qPCR data are represented for two separate internal controls. rpoD is used as the internal control in the manuscript file. Corresponding data with hcaT as the internal control are presented in the supplementary figures. The comparability of the RT-qPCR results with two separate internal controls was considered to verify the reproducibility of the findings.
Randomization	For every strain used in this study, cultures grown in in a medium with [NaCl] = 0.08 M were considered 'low salt' samples. Cultures grown in the presence of 0.3 M NaCl were considered 'high salt' samples. 'Low salt' samples at OD600=1 for which the culture medium was supplemented with NaCl to a final concentration of 0.3 M NaCl for 10 minutes were considered to be 'hyperosmotic shock' samples.<br><br>Randomisation was included our study since pre-determined conditions were chosen for samples to be taken from.
Blinding	Blinding was not necessary during this study since qPCR data was collected using a software: Bio-Rad CFX Manager™ (RRID:SCR_017251) Version 3.1 . Hi-C data was collected using an Illumina NextSeq 500 platform.

Reporting for specific materials, systems and methods

We require information from authors about some types of materials, experimental systems and methods used in many studies. Here, indicate whether each material, system or method listed is relevant to your study. If you are not sure if a list item applies to your research, read the appropriate section before selecting a response.

Materials & experimental systems

n/a	Involved in the study
☒	☐ Antibodies
☒	☐ Eukaryotic cell lines
☒	☐ Palaeontology and archaeology
☒	☐ Animals and other organisms
☒	☐ Clinical data
☒	☐ Dual use research of concern
☒	☐ Plants

Methods

n/a	Involved in the study
☒	☐ ChIP-seq
☒	☐ Flow cytometry
☒	☐ MRI-based neuroimaging
